# Supplementary material for: Unlocking the function promiscuity of old yellow enzyme to catalyze asymmetric Morita-Baylis-Hillman reaction
Source: Nat Commun. 2024 Jul 9;15:5737. doi: 10.1038/s41467-024-50141-2 (PMC11233575; doi:10.1038/s41467-024-50141-2)
Supplement: Supplementary file 1 — Supplementary Information [file 41467_2024_50141_MOESM1_ESM.pdf]

Supplementary Information for

Unlocking the function promiscuity of old yellow enzyme to catalyze asymmetric Morita-Baylis-Hillman reaction

Lei Wang[1,2,3], Yaoyun Wu[1,2,3], Jun Hu[1], Dejing Yin[2], Wanqing Wei[2,3], Jian Wen[1], Xiulai Chen[2,3], Cong Gao[2,3], Yiwen Zhou[1], Jia Liu[2,3], Guipeng Hu[1], Xiaomin Li[2,3], Jing Wu[1], Zhi Zhou[1], Liming Liu[2,3], Wei Song[1*]

[1]School of Life Sciences and Health Engineering, Jiangnan University, Wuxi 214122, China;

[2]School of Biotechnology, Jiangnan University, Wuxi 214122, China;

[3]Key Laboratory of Industrial Biotechnology of Ministry of Education, Jiangnan University, Wuxi 214122, China.

*Corresponding author: School of Life Sciences and Health Engineering, Jiangnan University, 1800 Lihu Road, Wuxi, 214122, China.

E-mail: weisong@jiangnan.edu.cn (Wei Song).

Table of contents

Supplementary Figures 2
Supplementary Tables 53
Supplementary Note Genetic constructions and optimized sequences 54

Supplementary Figures

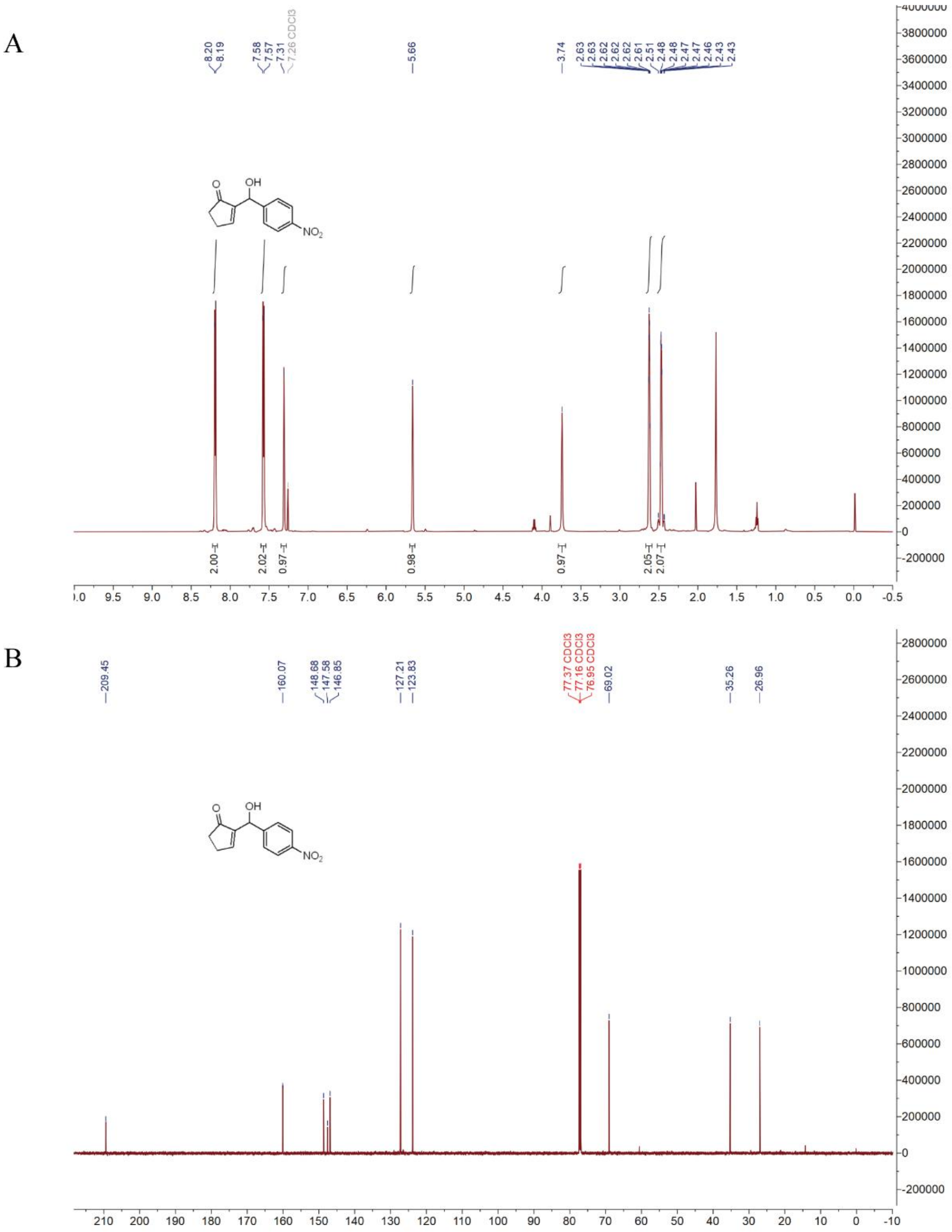

Supplementary Figure 1. NMR results of new products **3**. (**A**) ^{1}H-NMR spectra of **3**. (B) ^{13}C-NMR spectra of **3**.

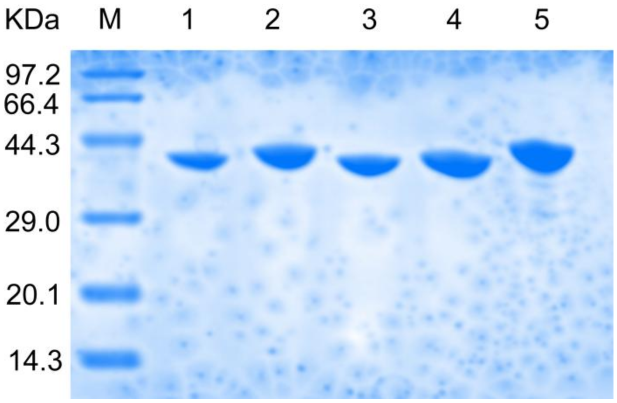

Supplementary Figure 2. SDS-PAGE analysis of different old yellow enzymes. Lane M: protein molecular weight marker. Lane 1: SDS-PAGE analysis of *Gk*OYE、Lane 2: SDS-PAGE analysis of GluER、Lane 3: SDS-PAGE analysis of XenA、Lane 4: SDS-PAGE analysis of NemA、Lane 5: SDS-PAGE analysis of MR. Three times the experiment was repeated with similar results. Source data are provided as a Source Data file.

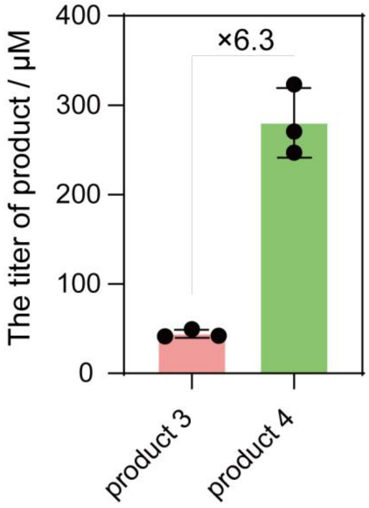

Supplementary Figure 3. Detection of the titer of product **3** and product **4** in the reaction system. n=3 independent biological experiments. Data are presented as mean values ± SD. Source data are provided as a Source Data file.

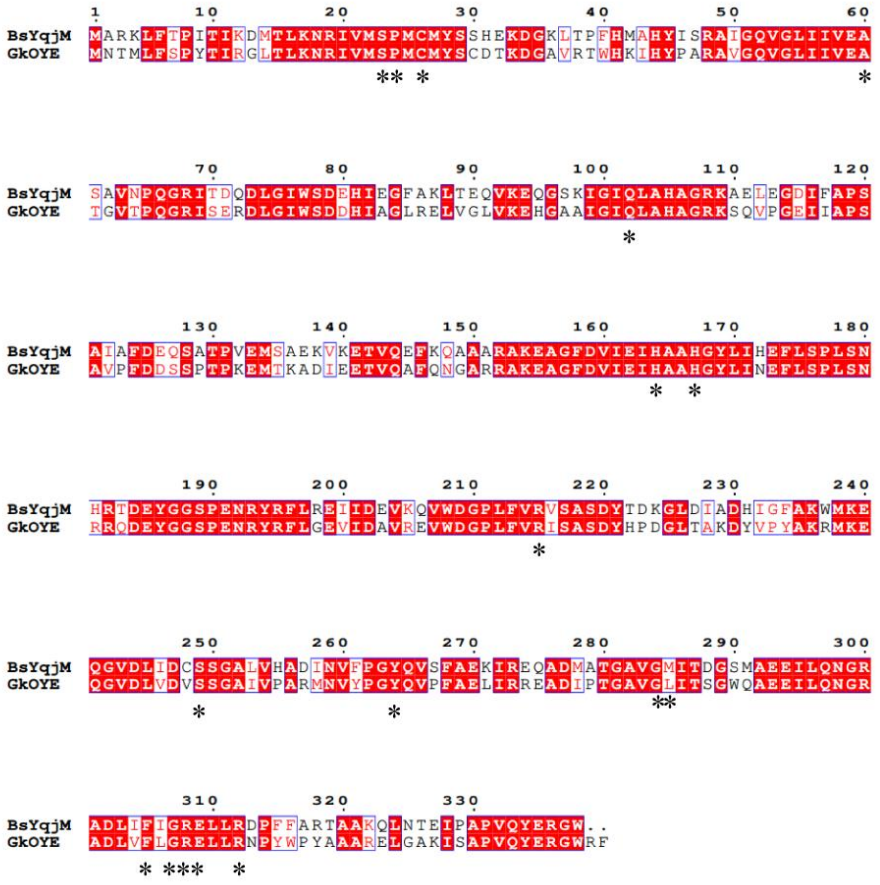

Supplementary Figure 4. The sequence alignment results of *Bs*YqjM and *Gk*OYE.

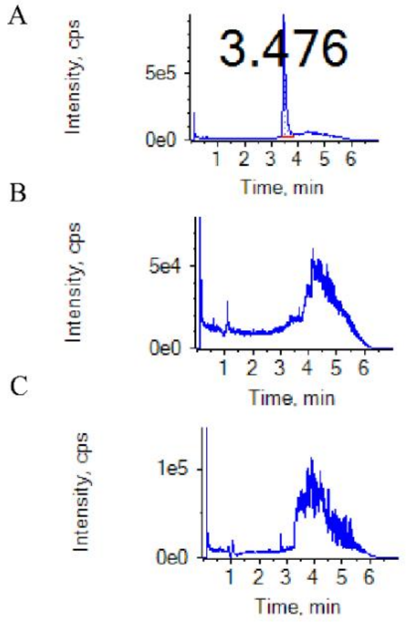

Supplementary Figure 5. Detection of FMN in boiled protein supernatant by LC-MS. (A) Standard sample of FMN, concentration 0.1ppm. (B) Blank control. (C) Samples.

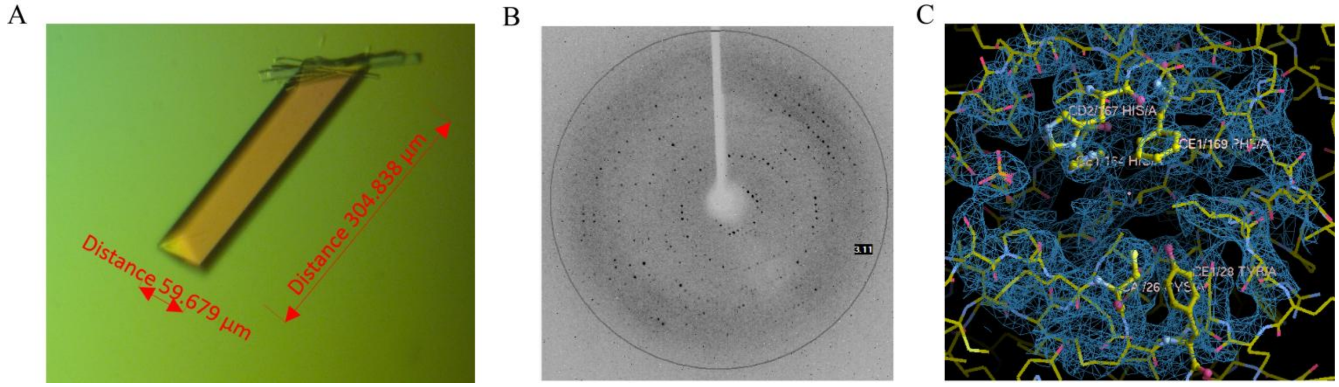

Supplementary Figure 6. The crystallization of *apo-Gk*OYE.8. (A) The crystal of *apo-Gk*OYE.8 under initial conditions; (B) The diffraction pattern of the crystal; (C) Electron density maps (2Fo-Fc) is contoured at 1.0 σ in blue mesh.

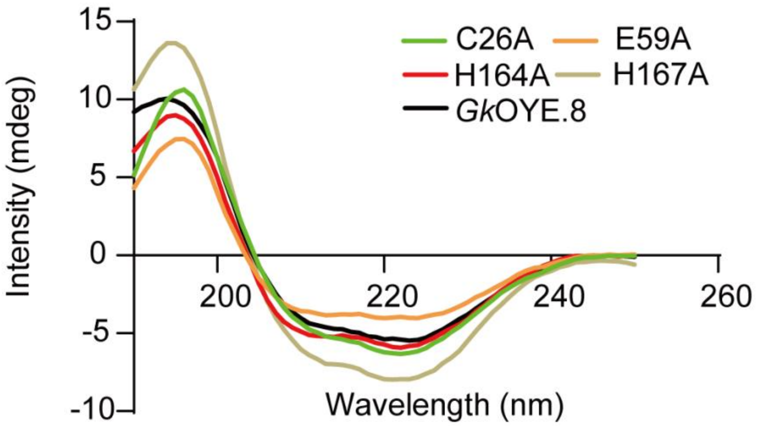

	Helix	Strand	Turns	Unordered
**C26A**	**26.1**	**16.6**	**17.4**	**40.3**
**E59A**	**26.8**	**15.3**	**15.4**	**40.0**
**H164A**	**30.6**	**20.3**	**20.3**	**29.3**
**H167A**	**26.2**	**17**	**17.6**	**40.4**
***Gk*OYE.8**	**26**	**16.5**	**17.4**	**40.3**

Supplementary Figure 7.The secondary structure of the key mutant protein was verified by circular dichroism scans. Three times the experiment was repeated with similar results.

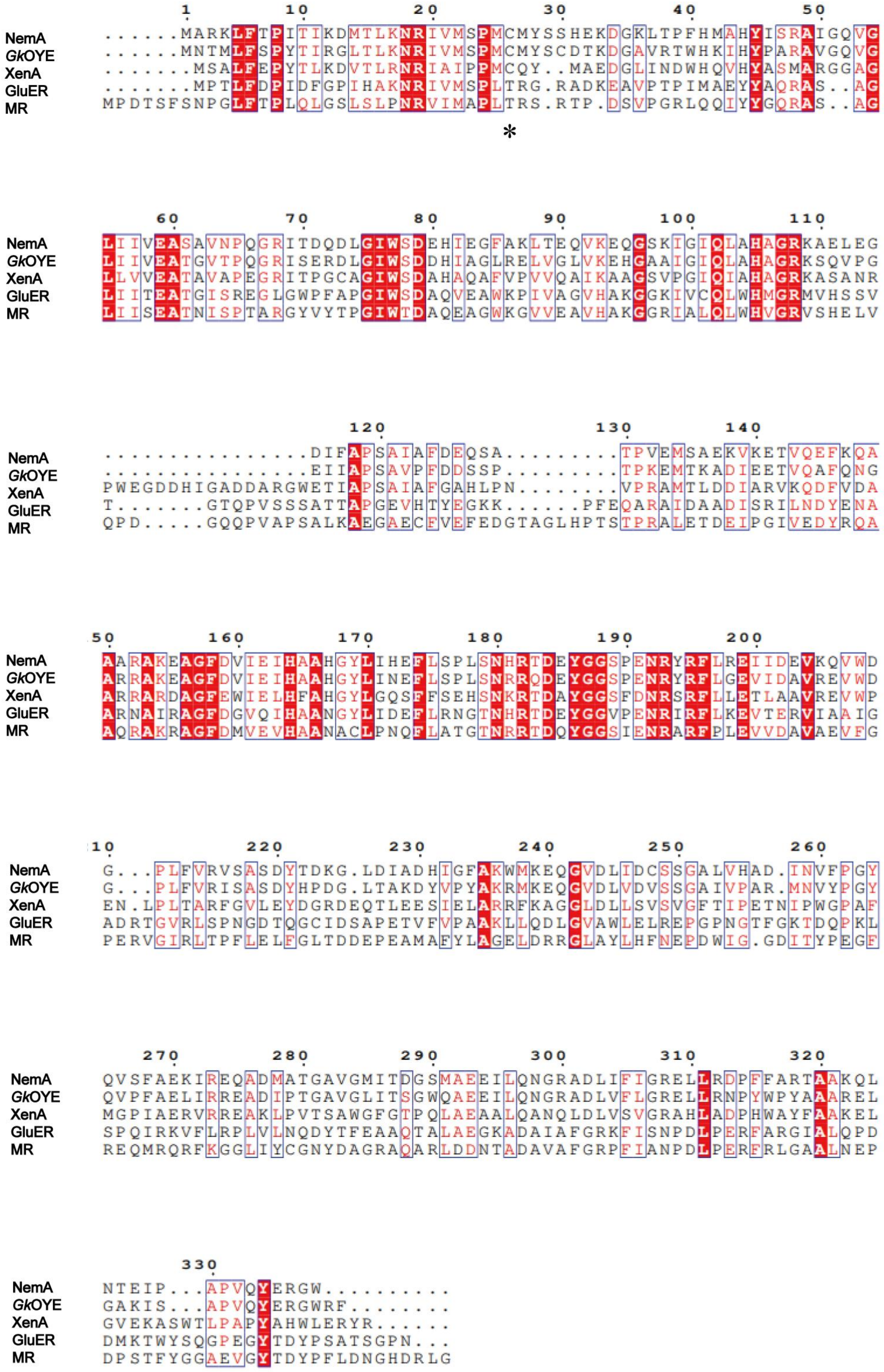

Supplementary Figure 8. The sequence alignment results of different old yellow enzymes.

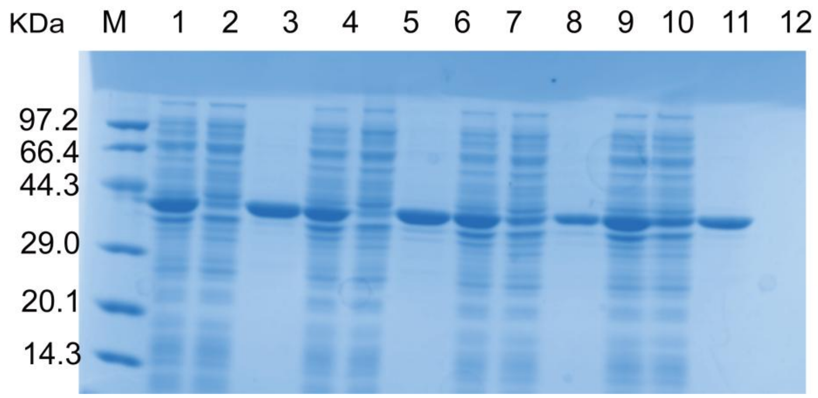

Supplementary Figure 9. SDS-PAGE analysis of the expression of representative mutants of E59 residues. Lane M: protein molecular weight marker; Lane 1-3: SDS-PAGE analysis of *E.coli* (*Gk*OYE^{E59A}), (Lane 1: Whole cell disruption solution; Lane 2: Supernatant; Lane 3: Precipitation); Lane 4-6: SDS-PAGE analysis of *E.coli* (*Gk*OYE^{E59W}), (Lane 4: Whole cell disruption solution; Lane 5: Supernatant; Lane 6: Precipitation); Lane 7-9: SDS-PAGE analysis of *E.coli* (*Gk*OYE^{E59R}), (Lane 7: Whole cell disruption solution; Lane 8: Supernatant; Lane 9: Precipitation); Lane 10-12: SDS-PAGE analysis of *E.coli* (*Gk*OYE^{E59M}), (Lane 10: Whole cell disruption solution; Lane 11: Supernatant; Lane 12: Precipitation). Three times the experiment was repeated with similar results. Source data are provided as a Source Data file.

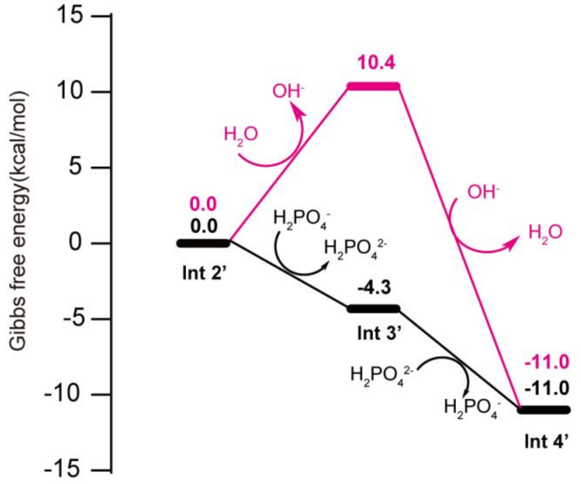

Supplementary Figure 10. DFT calculations were conducted to compare the energies of the two proton transfer modes. The black line represents the proton transfer involving phosphate anion, while the rose line indicates a water-mediated proton transfer. The results indicated that mode of phosphate anion is more exothermic than water, thus confirming the phosphate anion may accelerate the proton transfer.

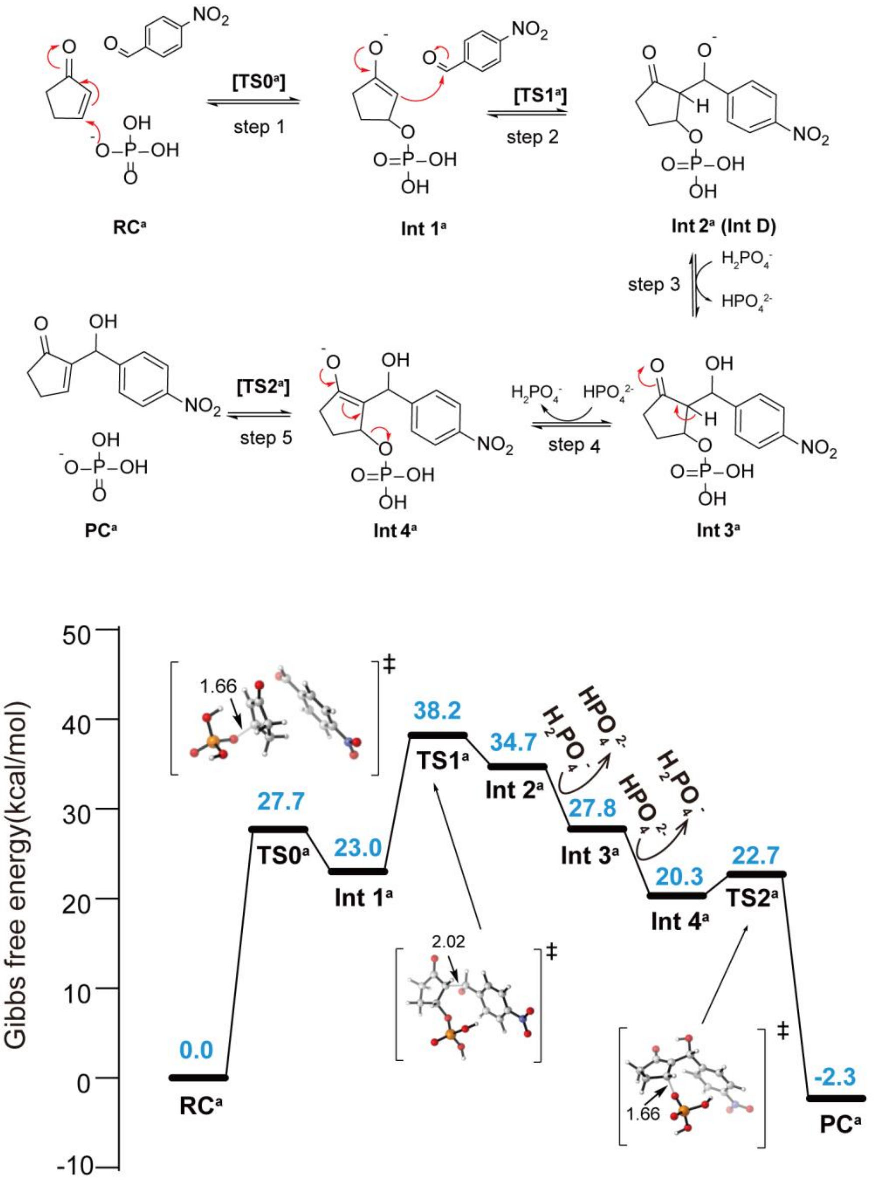

Supplementary Figure 11. The proposed mechanism and Gibbs free energy profile of MBH reaction without enzyme calculated by DFT. The putative mechanism of MBH reaction consists of 5 steps. The step 1: Michael addition. $H_2PO_4^-$ as a nucleophile attacks substrate **1**. The step 2: Aldol reaction. The step 3 and 4: Proton transfer. The step 5: Elimination. In the background reaction without enzyme, the transition state [**TS0**] and [**TS1**] have the higher energy barriers (27.7 kcal/mol and 38.2 kcal/mol, respectively). Both of these steps also have a higher energy barrierS than the theozyme model. So it can be concluded that residues C26 and E59 improve the catalytic efficiency of the reaction by reducing the energy barriers of these two higher energy barrier steps.

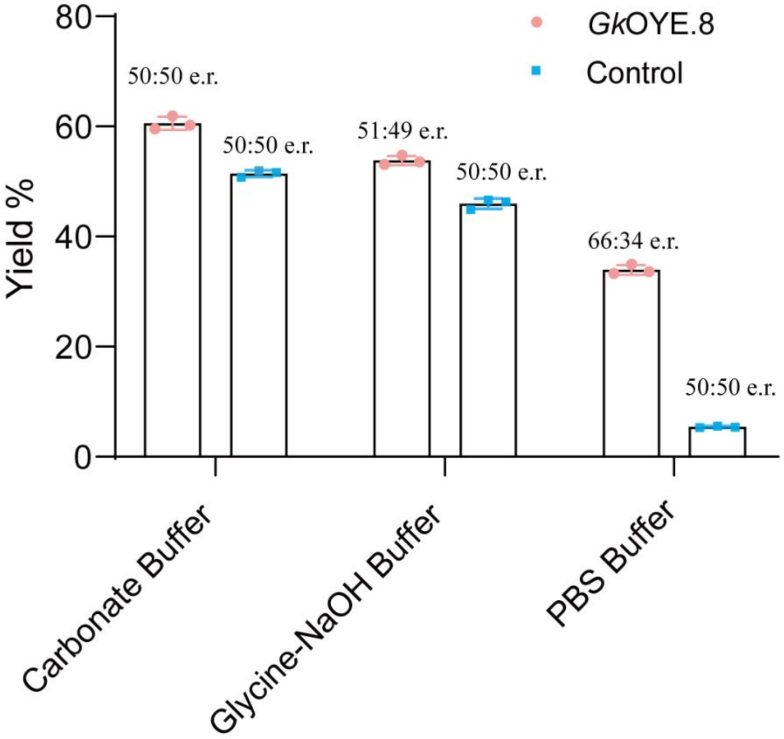

Supplementary Figure 12. The effect of different buffers at pH=10 on the MBH reaction was investigated. The reactions were carried out using 5 mM 1, 1 mM 2, and 100 μM purified protein in different buffer (pH 10.0) with 3% methanol (MeOH) as a co-solvent. Control: Background reaction without enzyme. n=3 independent biological experiments. Data are presented as mean values ± SD. Source data are provided as a Source Data file.

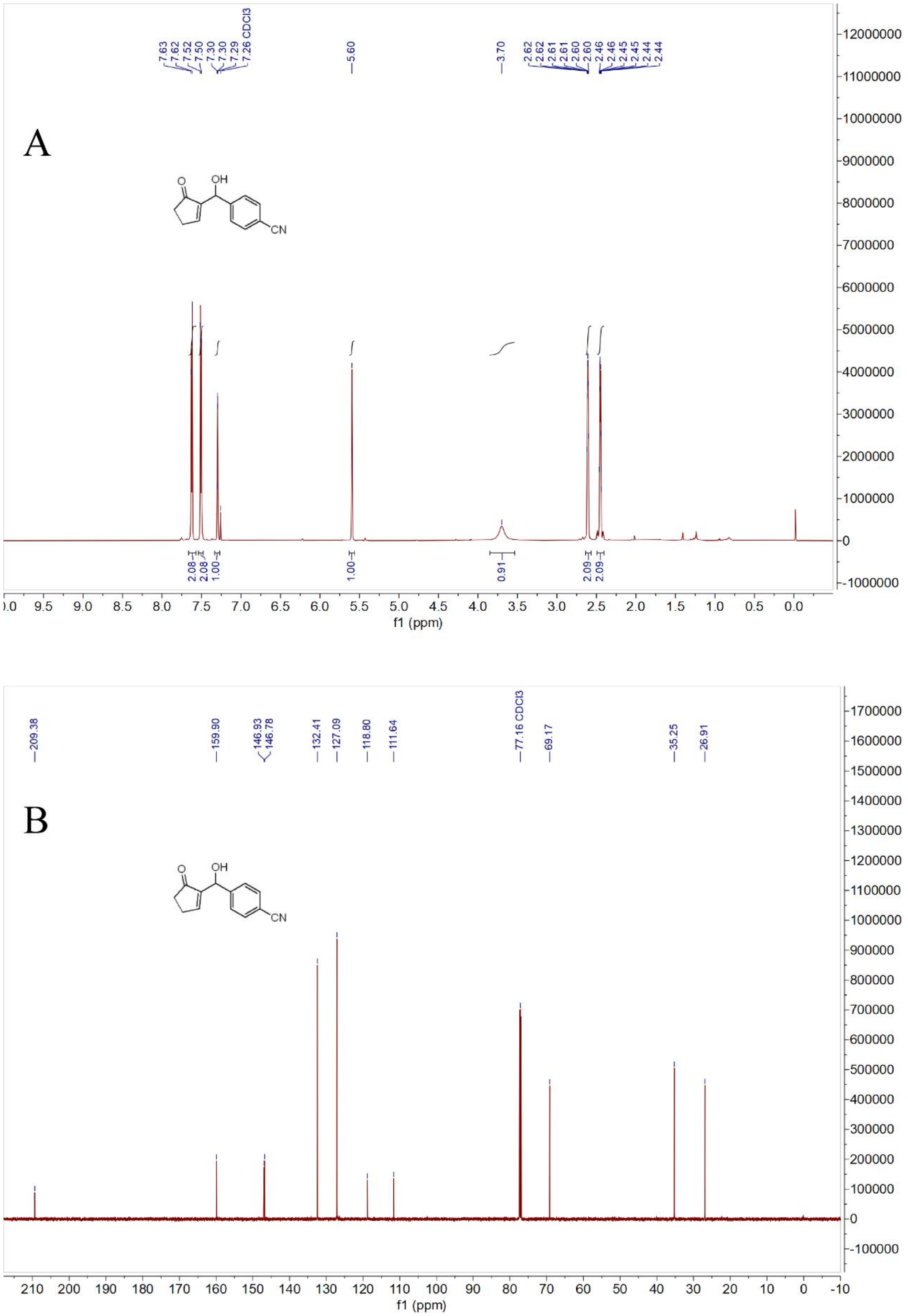

Supplementary Figure 13. NMR spectra of **3a**. (A) ^{1}H-NMR spectra of **3a**. (B) ^{13}C-NMR spectra of **3a**.

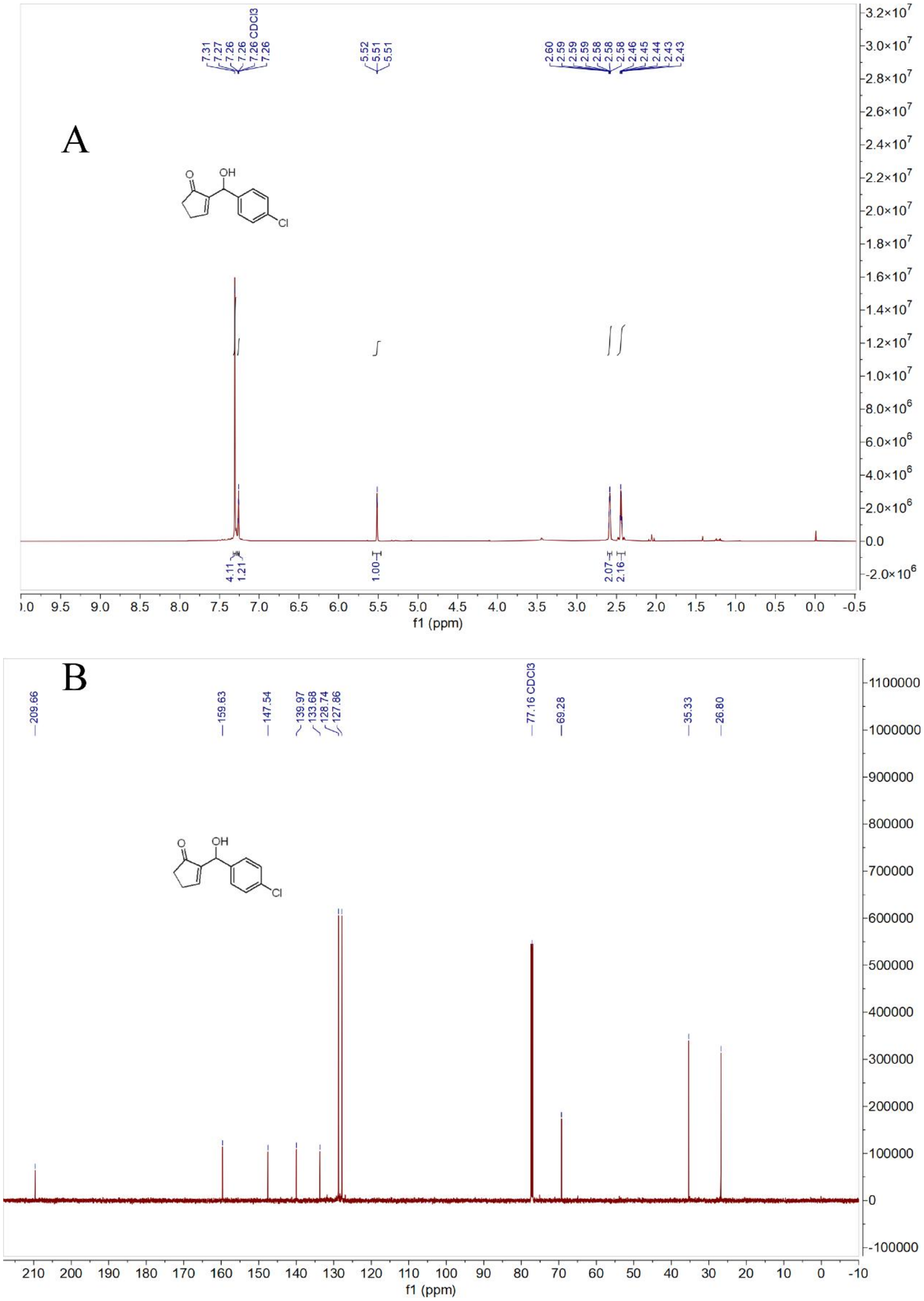

Supplementary Figure 14. NMR spectra of **3b**. (A) ^{1}H-NMR spectra of **3b**. (B) ^{13}C-NMR spectra of **3b**.

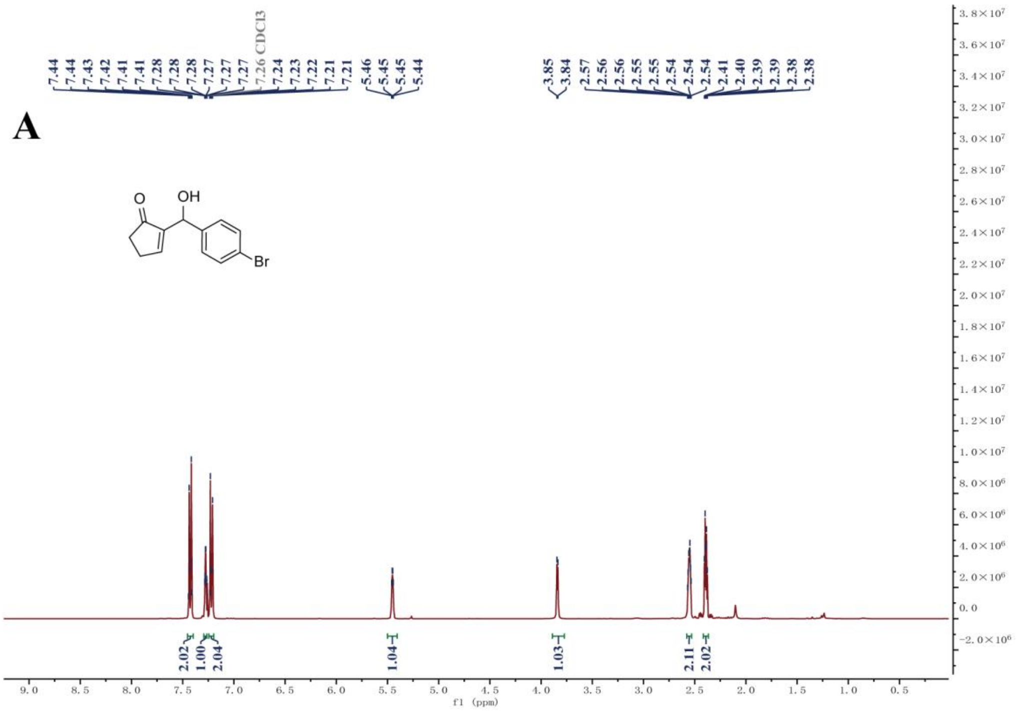

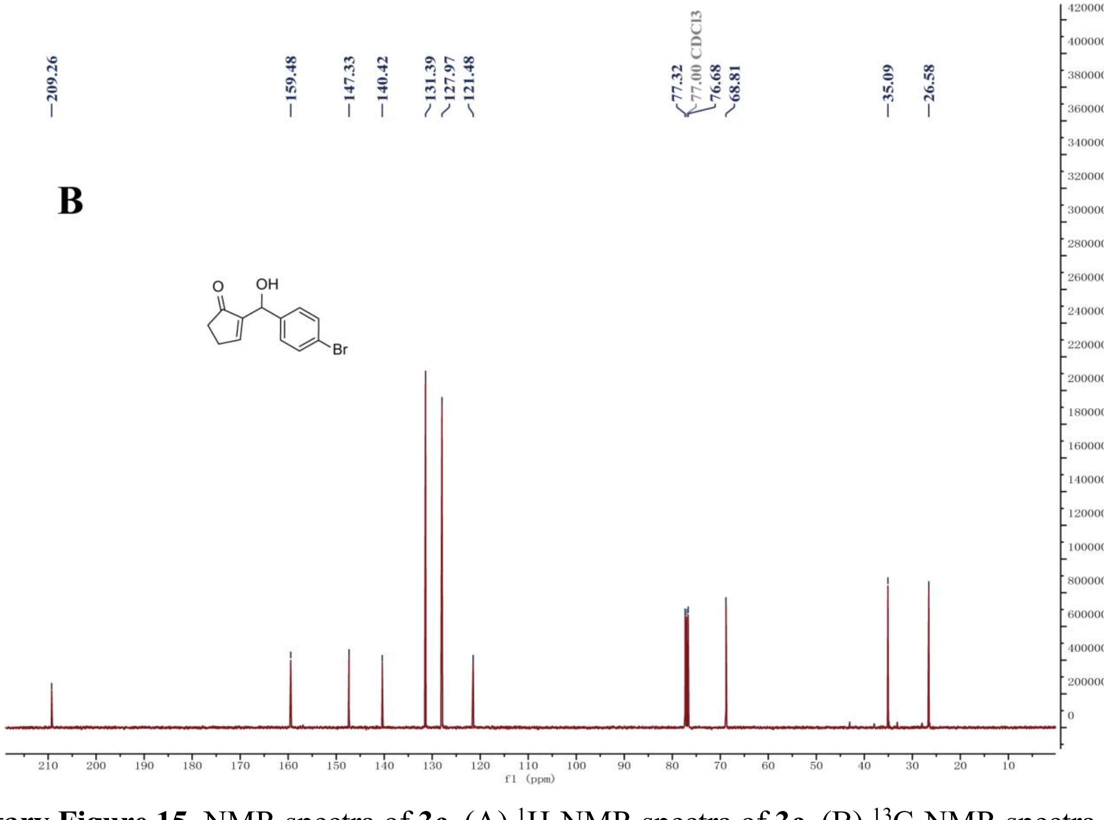

Supplementary Figure 15. NMR spectra of **3c**. (A) ^{1}H-NMR spectra of **3c**. (B) ^{13}C-NMR spectra of **3c**.

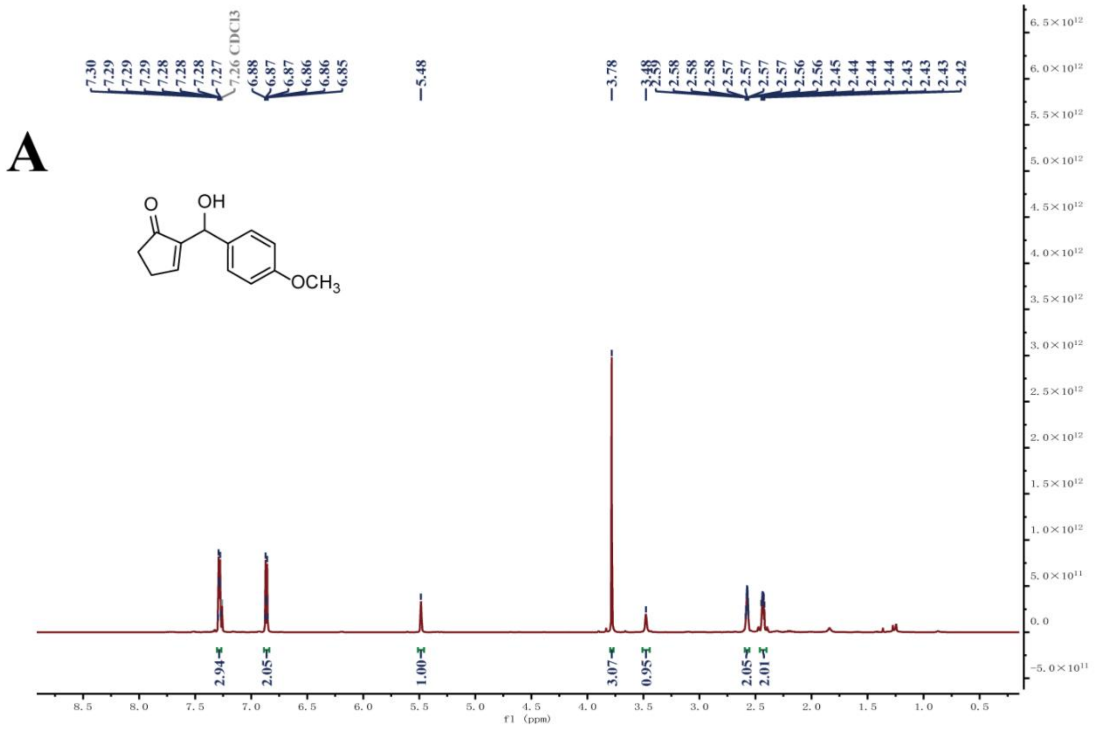

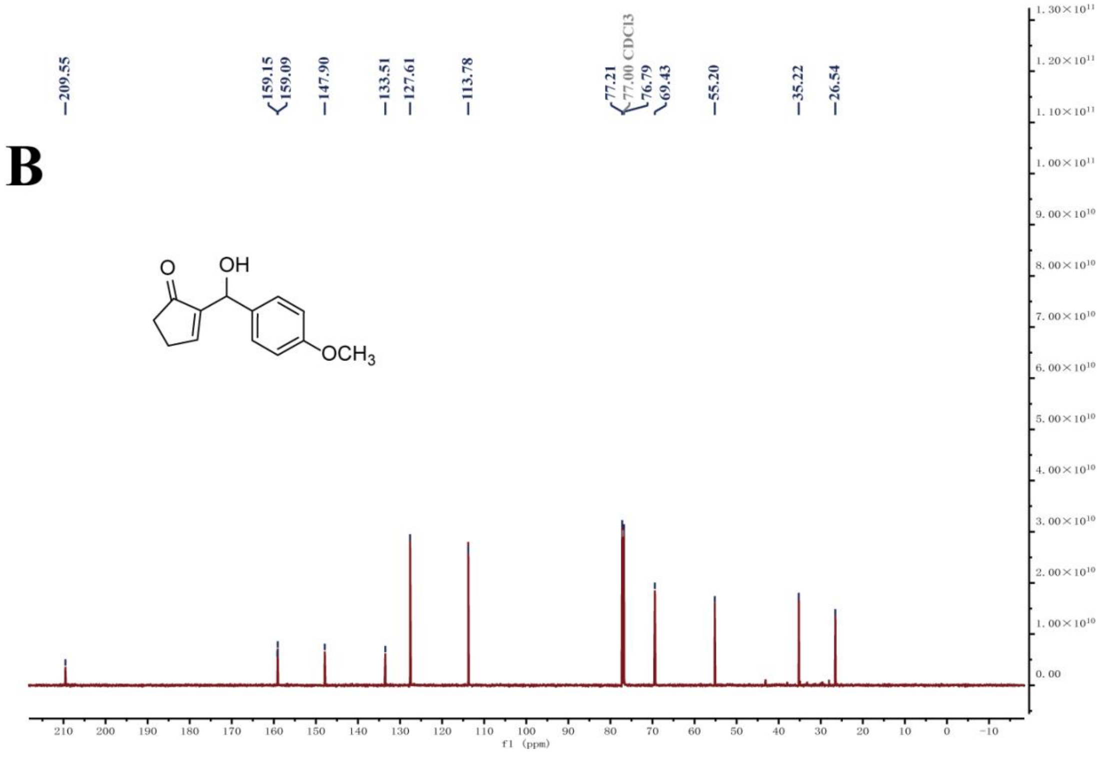

Supplementary Figure 16. NMR spectra of **3d**. (A) ^{1}H-NMR spectra of **3d**. (B) ^{13}C-NMR spectra of **3d**.

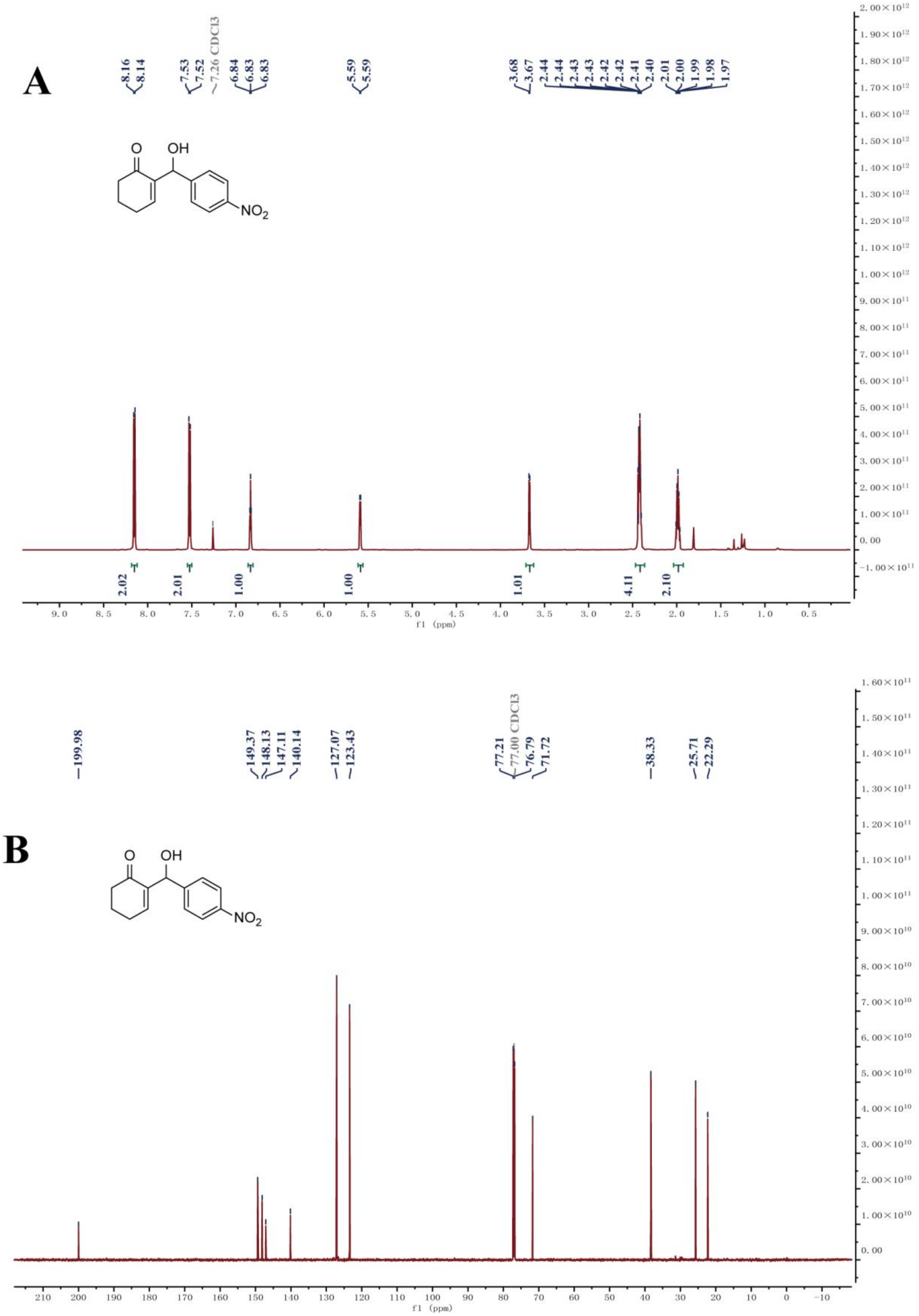

Supplementary Figure 17. NMR spectra of **3e**. (A) ^{1}H-NMR spectra of **3e**. (B) ^{13}C-NMR spectra of **3e**.

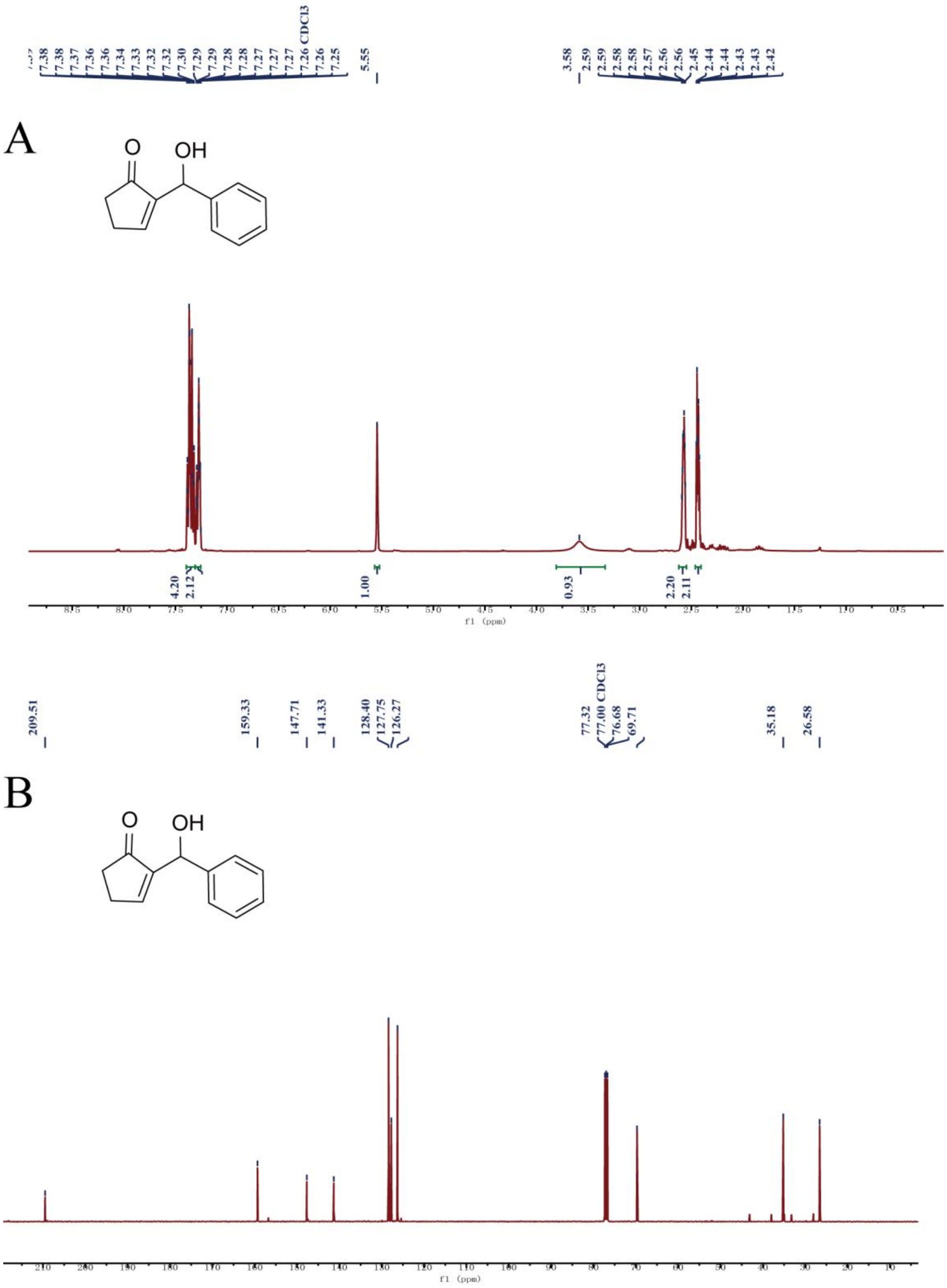

Supplementary Figure 18. NMR spectra of **3f**. (A) ^{1}H-NMR spectra of **3f**. (B) ^{13}C-NMR spectra of **3f**.

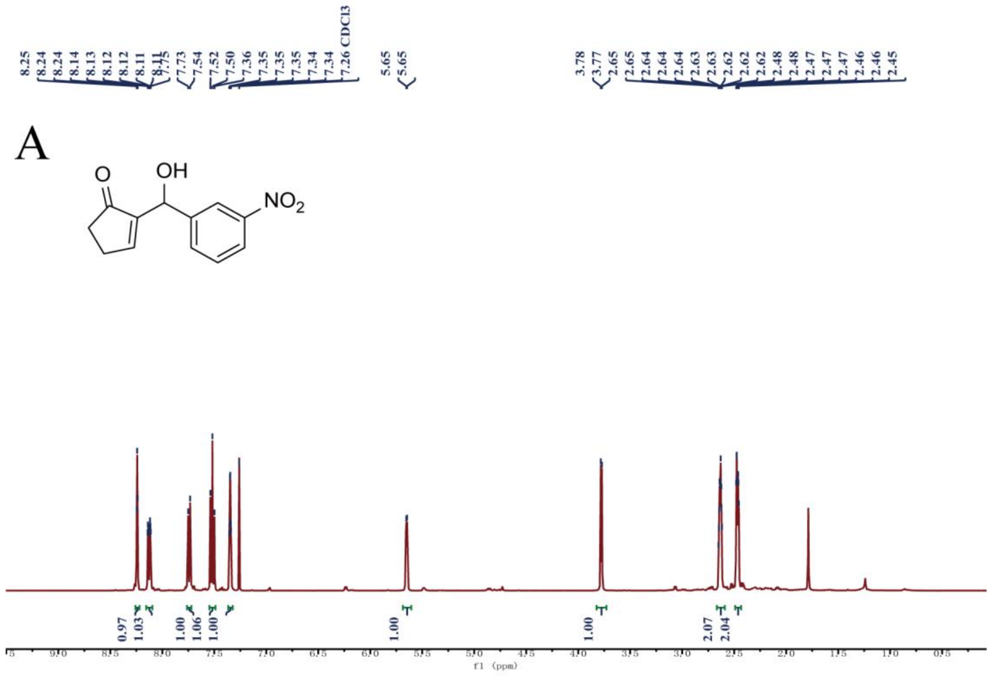

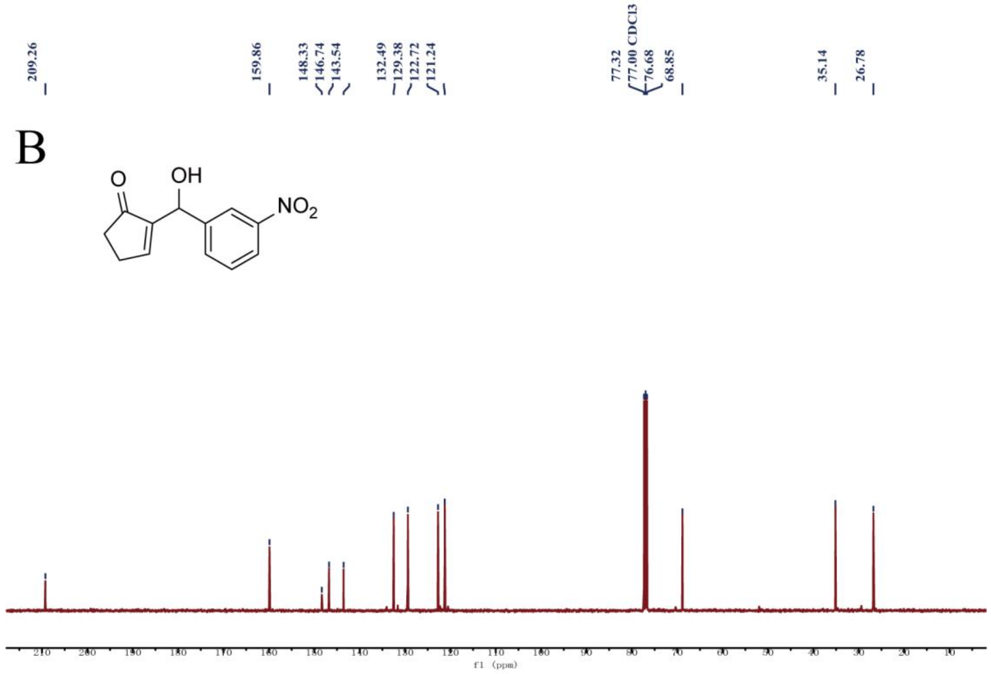

Supplementary Figure 19. NMR spectra of **3j**. (A) ^{1}H-NMR spectra of **3j**. (B) ^{13}C-NMR spectra of **3j**.

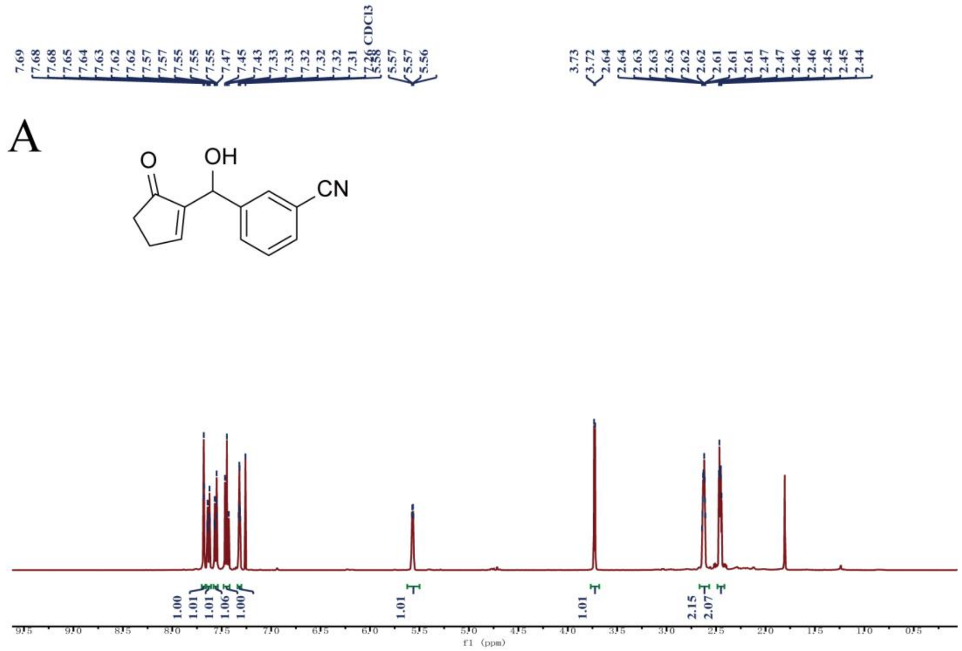

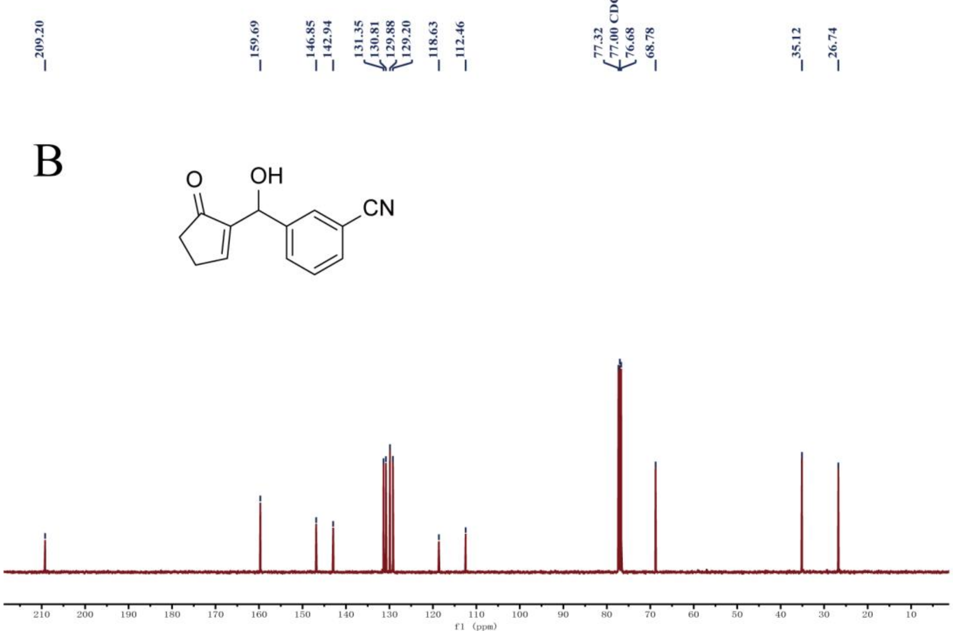

Supplementary Figure 20. NMR spectra of **3h**. (A) ^{1}H-NMR spectra of **3h**. (B) ^{13}C-NMR spectra of **3h**.

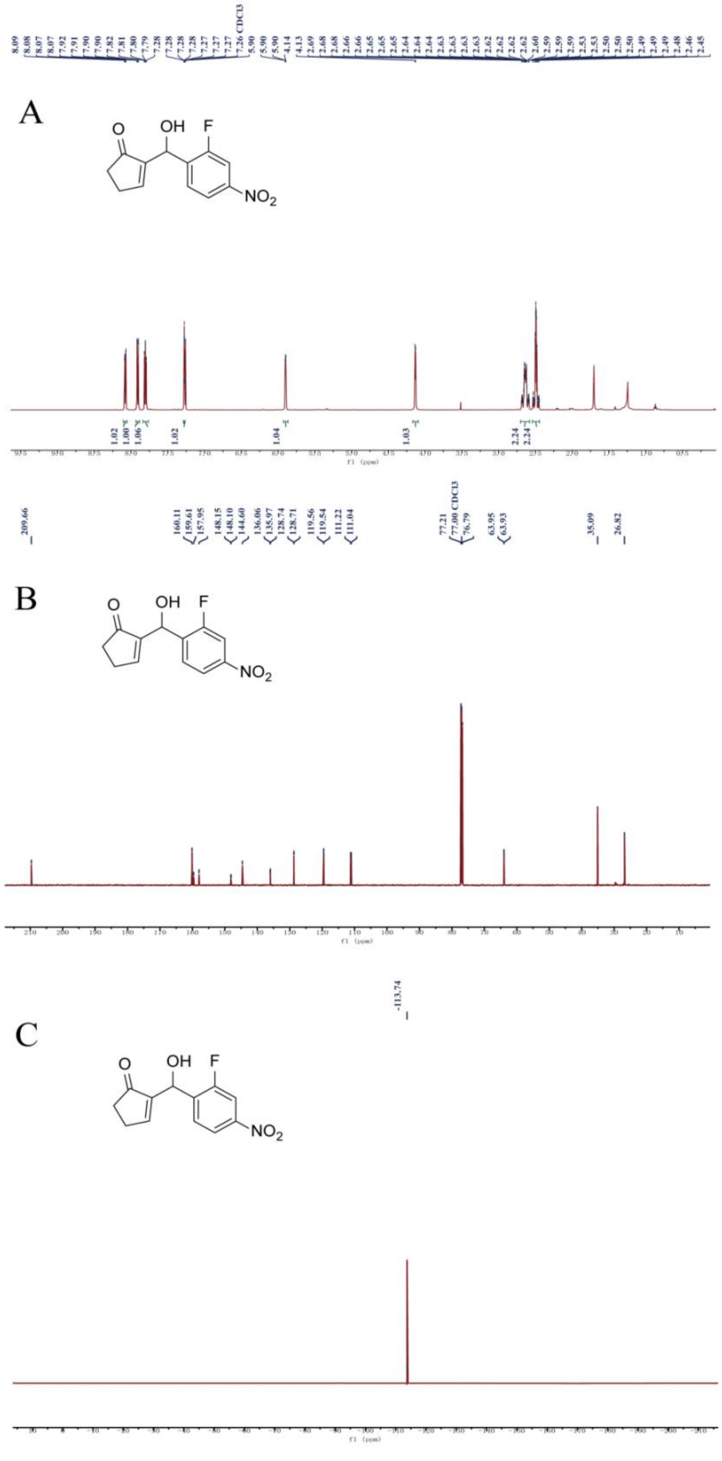

Supplementary Figure 21. NMR spectra of **3i**. (A) ^{1}H-NMR spectra of **3i**. (B) ^{13}C-NMR spectra of **3i**. (C) ^{19}F-NMR spectra of **3i**.

A

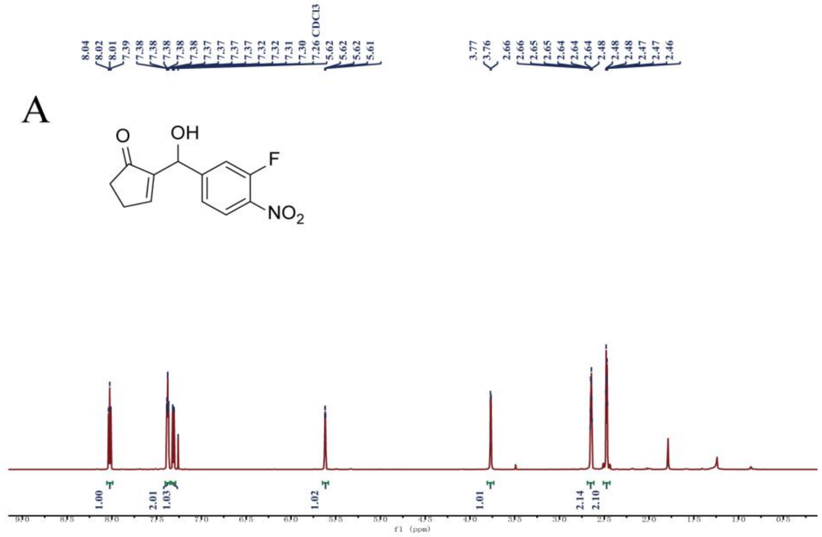

B

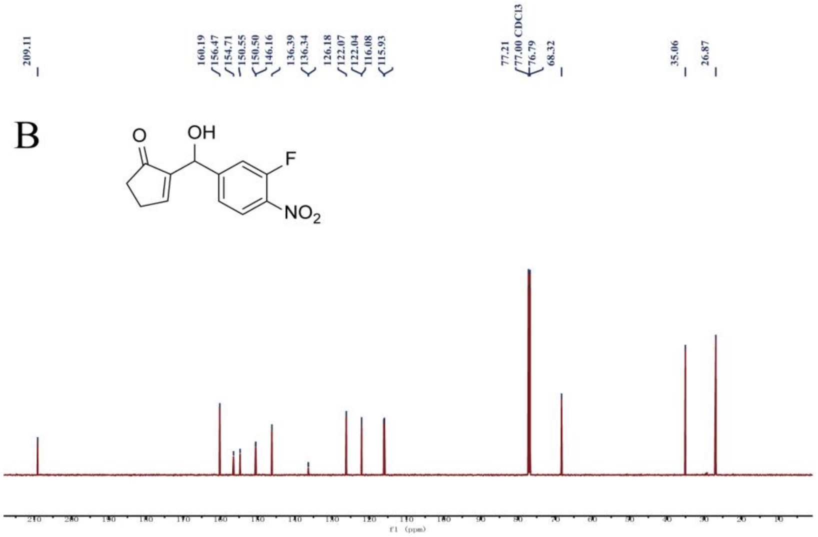

C

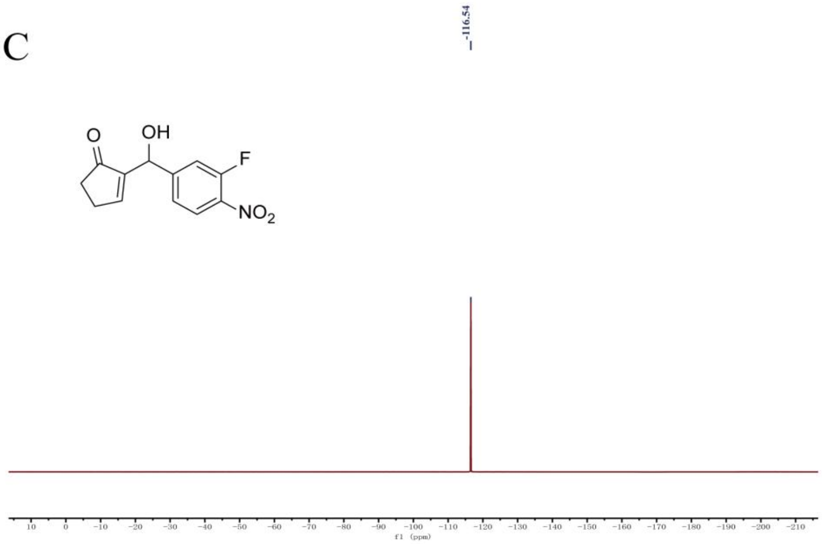

Supplementary Figure 22. NMR spectra of **3g**. (A) ^{1}H-NMR spectra of **3g**. (B) ^{13}C-NMR spectra of **3g**. (C) ^{19}F-NMR spectra of **3g**.

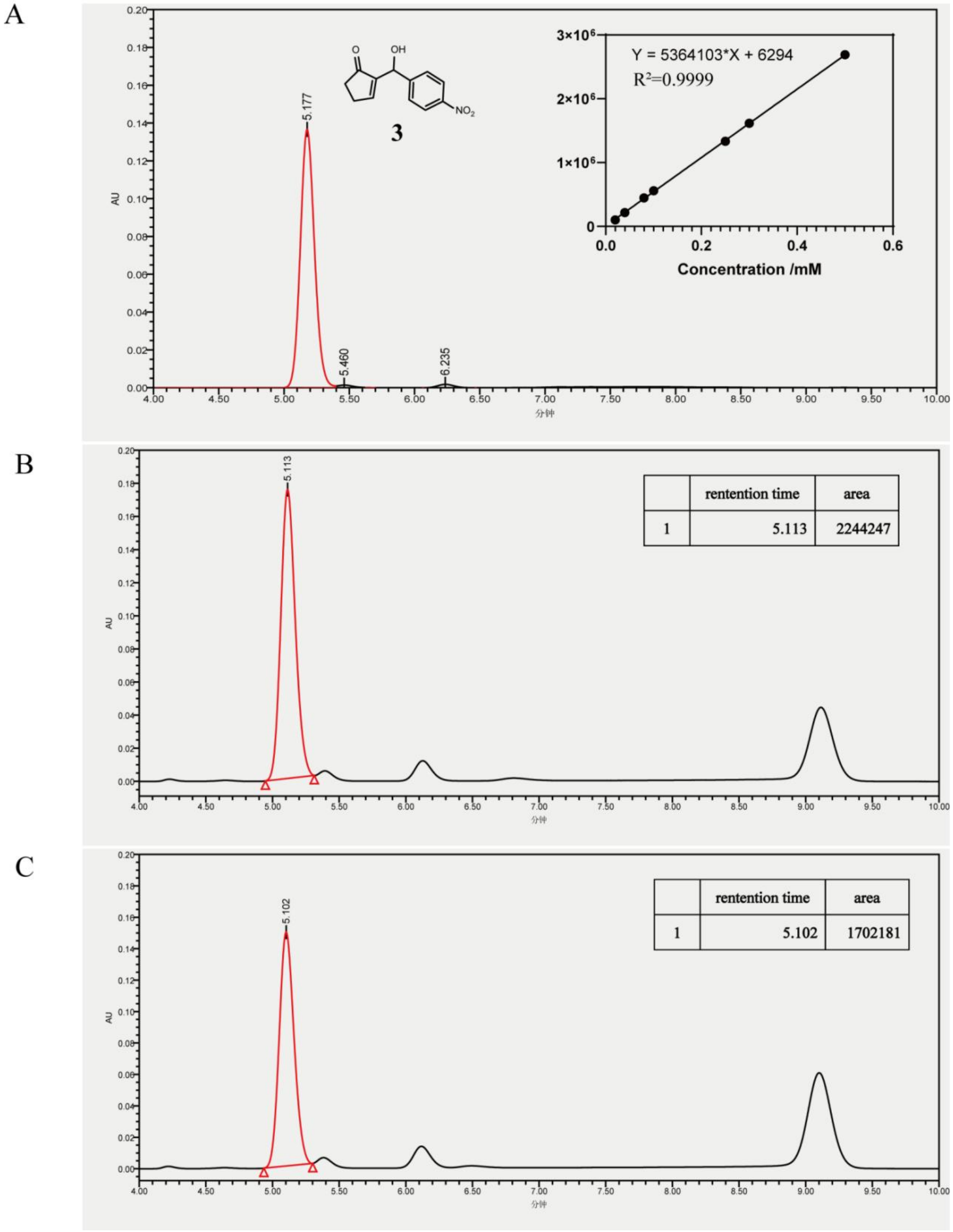

Supplementary Figure 23. HPLC chromatograms of adducts **3**. (A) The standard of **3**. (B) Sample from biotransformation by *Gk*OYE.11. (C) Sample from biotransformation by *Gk*OYE.13.

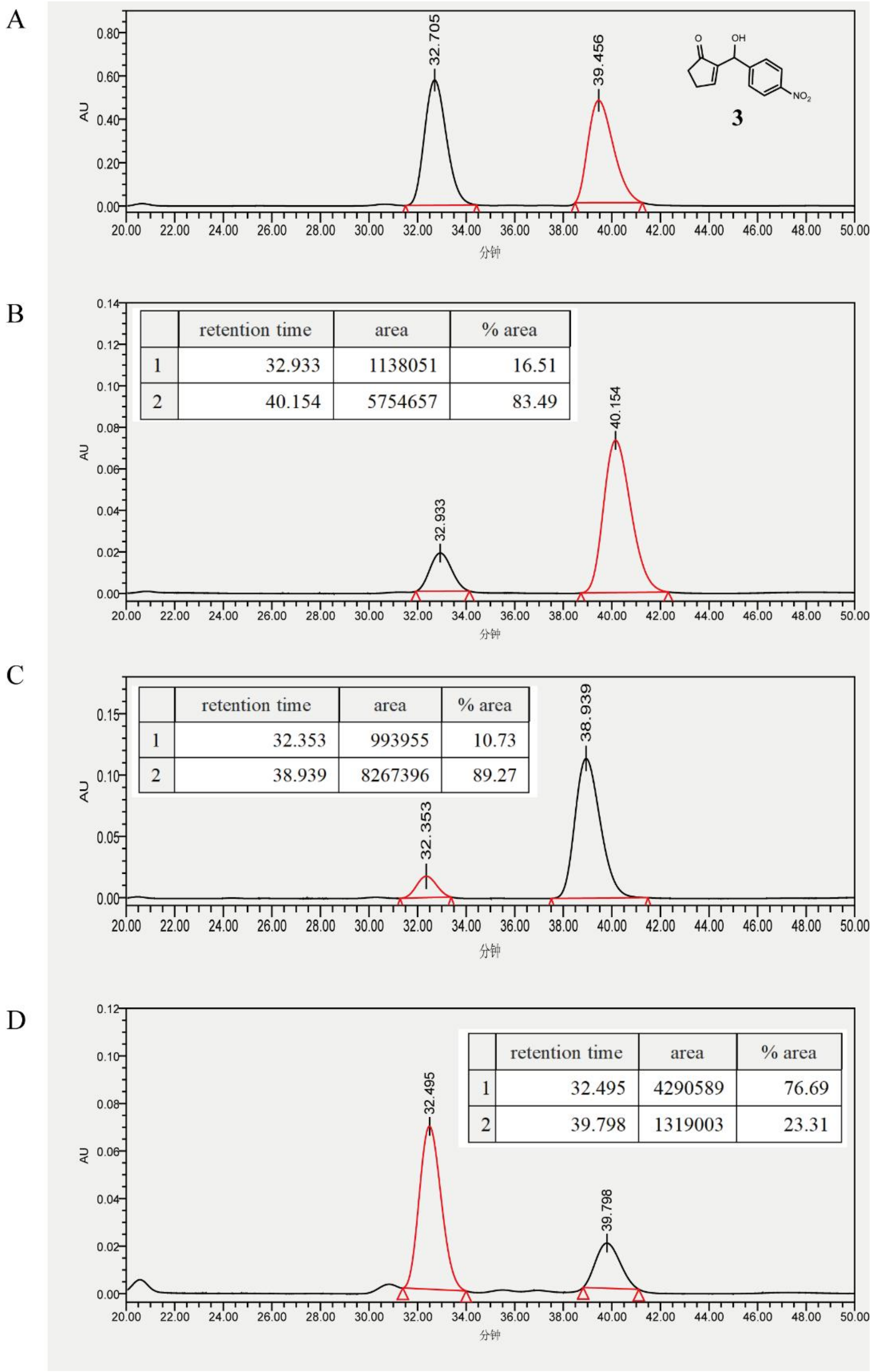

Supplementary Figure 24. Chiral HPLC chromatograms of adducts **3**. (A) The standard of **3**. (B) Sample from biotransformation by BH32.14. (C) Sample from biotransformation by *Gk*OYE.11. (D) Sample from biotransformation by *Gk*OYE.13.

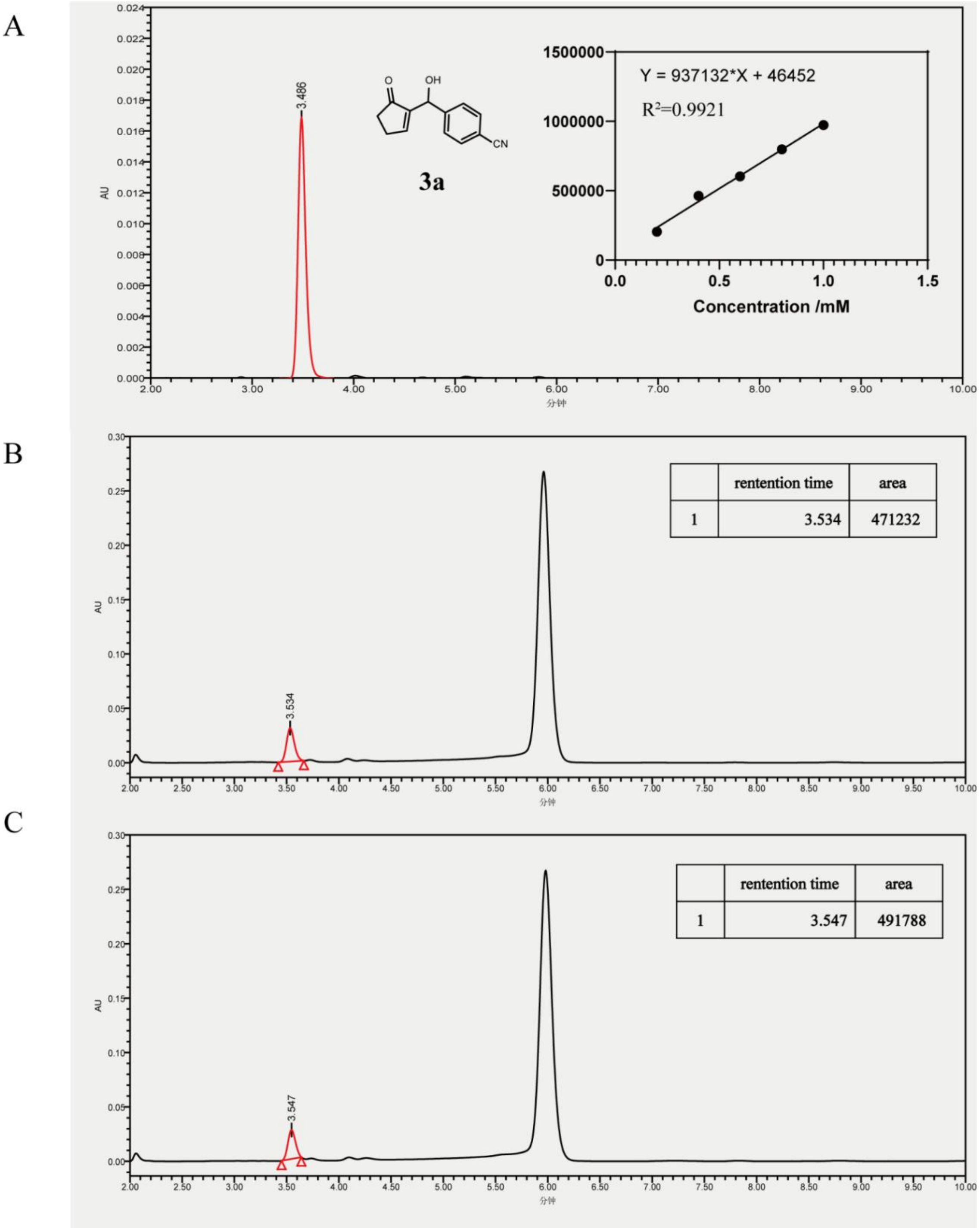

Supplementary Figure 25. HPLC chromatograms of adducts **3a**. (A) The standard of **3a**. (B) Sample from biotransformation by *Gk*OYE.11. (C) Sample from biotransformation by *Gk*OYE.13.

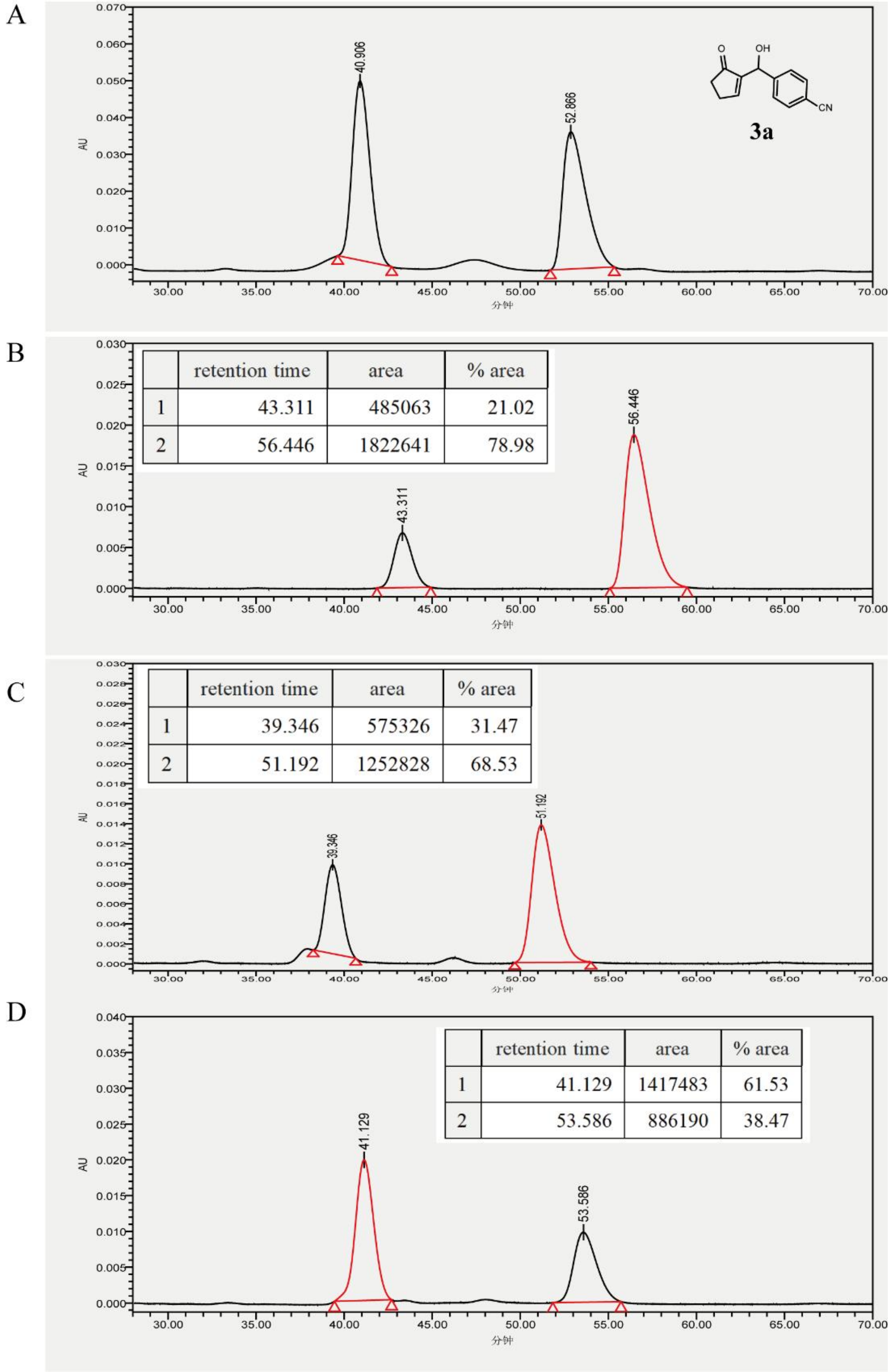

B

	retention time	area	% area
1	43.311	485063	21.02
2	56.446	1822641	78.98

C

	retention time	area	% area
1	39.346	575326	31.47
2	51.192	1252828	68.53

D

	retention time	area	% area
1	41.129	1417483	61.53
2	53.586	886190	38.47

Supplementary Figure 26. Chiral HPLC chromatograms of adducts **3a**. (A) The standard of **3a**. (B) Sample from biotransformation by BH32.14. (C) Sample from biotransformation by *Gk*OYE.11. (D) Sample from biotransformation by *Gk*OYE.13.

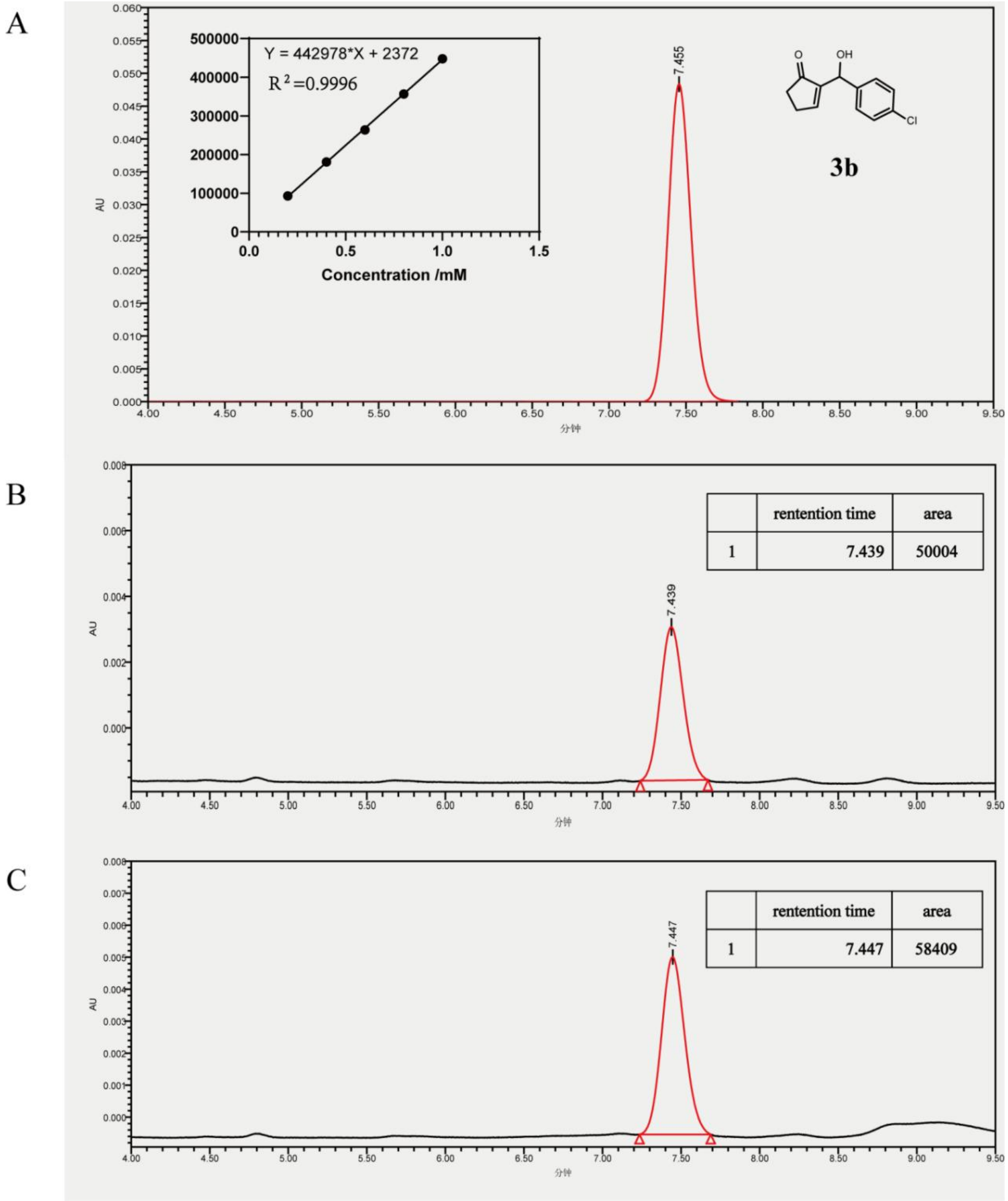

Supplementary Figure 27. HPLC chromatograms of adducts **3b**. (A) The standard of **3b**. (B) Sample from biotransformation by *Gk*OYE.11. (C) Sample from biotransformation by *Gk*OYE.13.

A

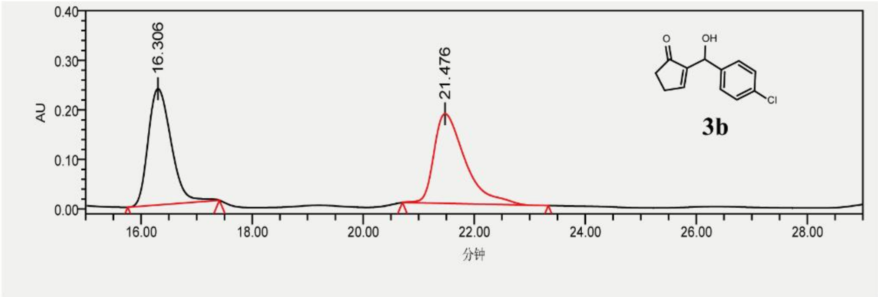

B

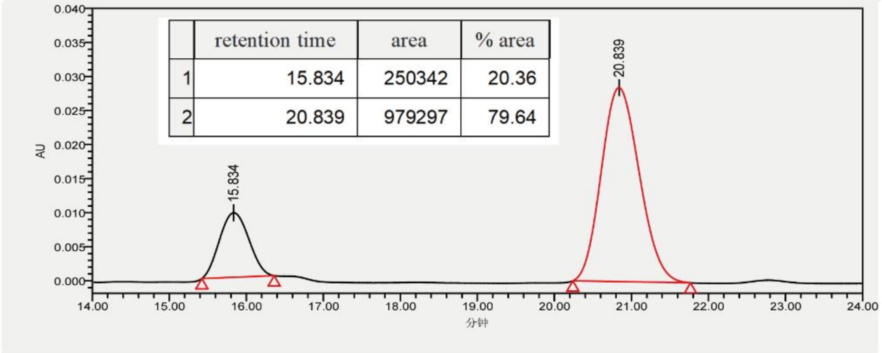

	retention time	area	% area
1	15.834	250342	20.36
2	20.839	979297	79.64

C

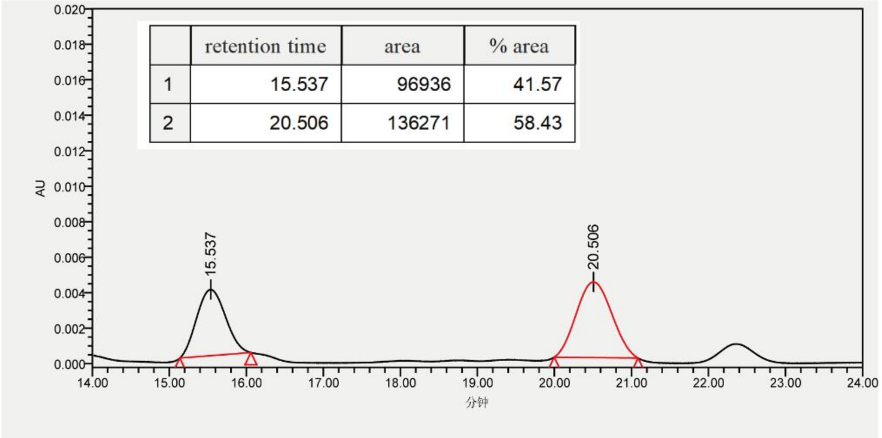

	retention time	area	% area
1	15.537	96936	41.57
2	20.506	136271	58.43

D

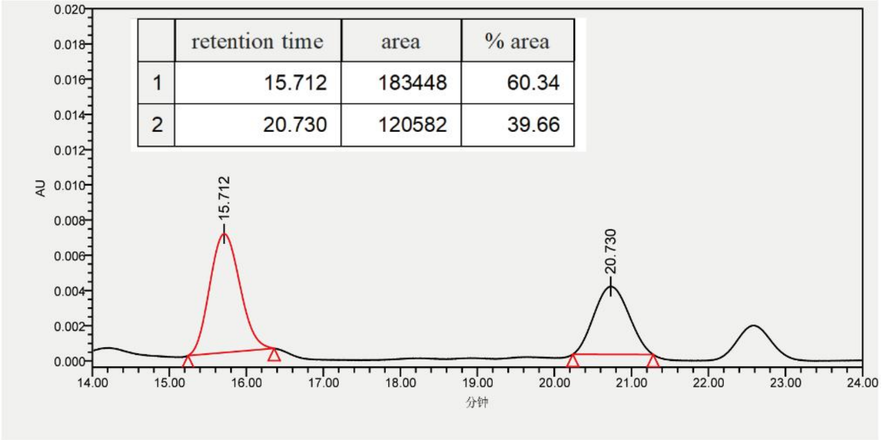

	retention time	area	% area
1	15.712	183448	60.34
2	20.730	120582	39.66

Supplementary Figure 28. Chiral HPLC chromatograms of adducts **3b**. (A) The standard of **3b**. (B) Sample from biotransformation by BH32.14. (C) Sample from biotransformation by *Gk*OYE.11. (D) Sample from biotransformation by *Gk*OYE.13.

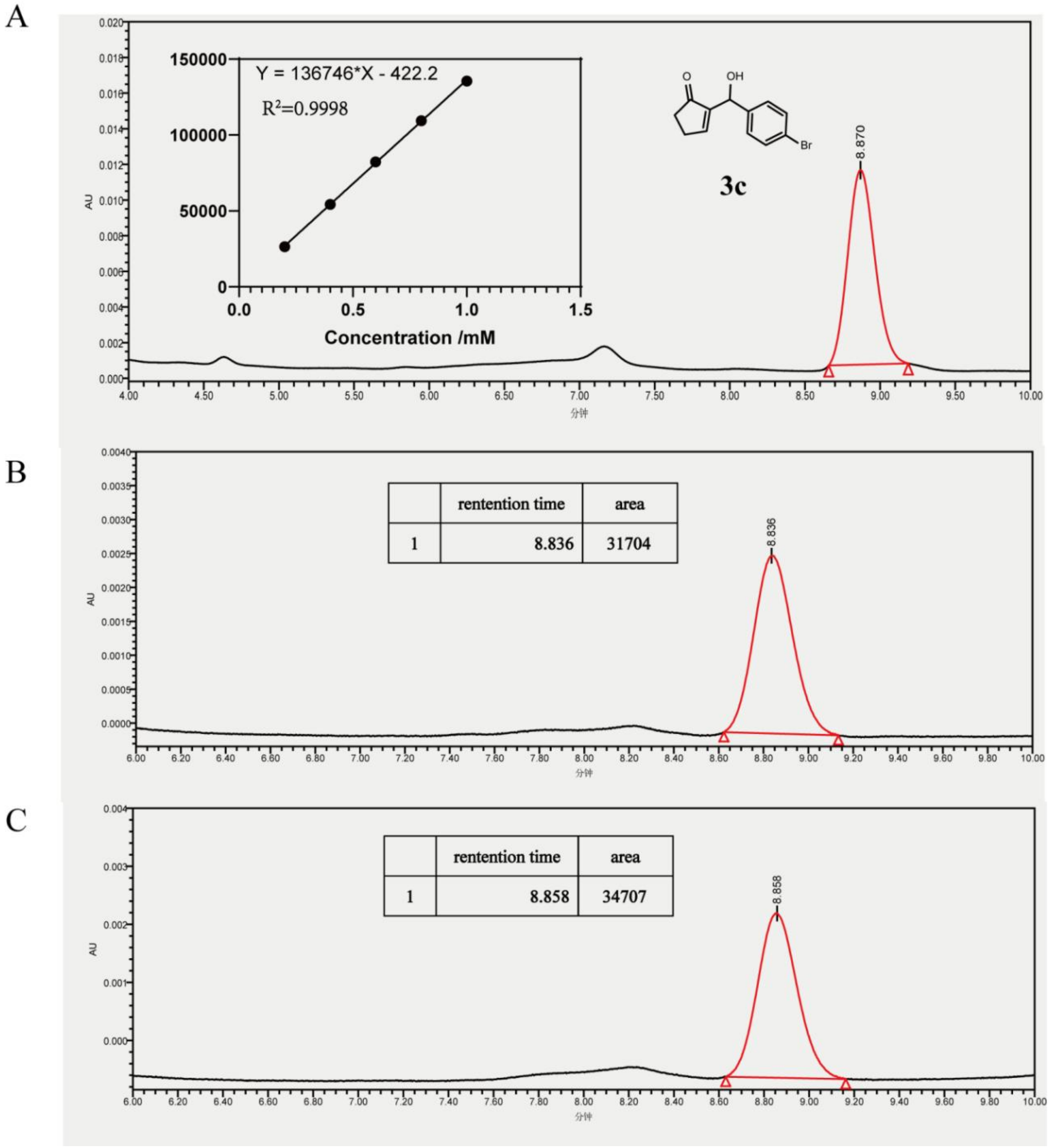

Supplementary Figure 29. HPLC chromatograms of adducts **3c**. (A) The standard of **3c**. (B) Sample from biotransformation by *Gk*OYE.11. (C) Sample from biotransformation by *Gk*OYE.13.

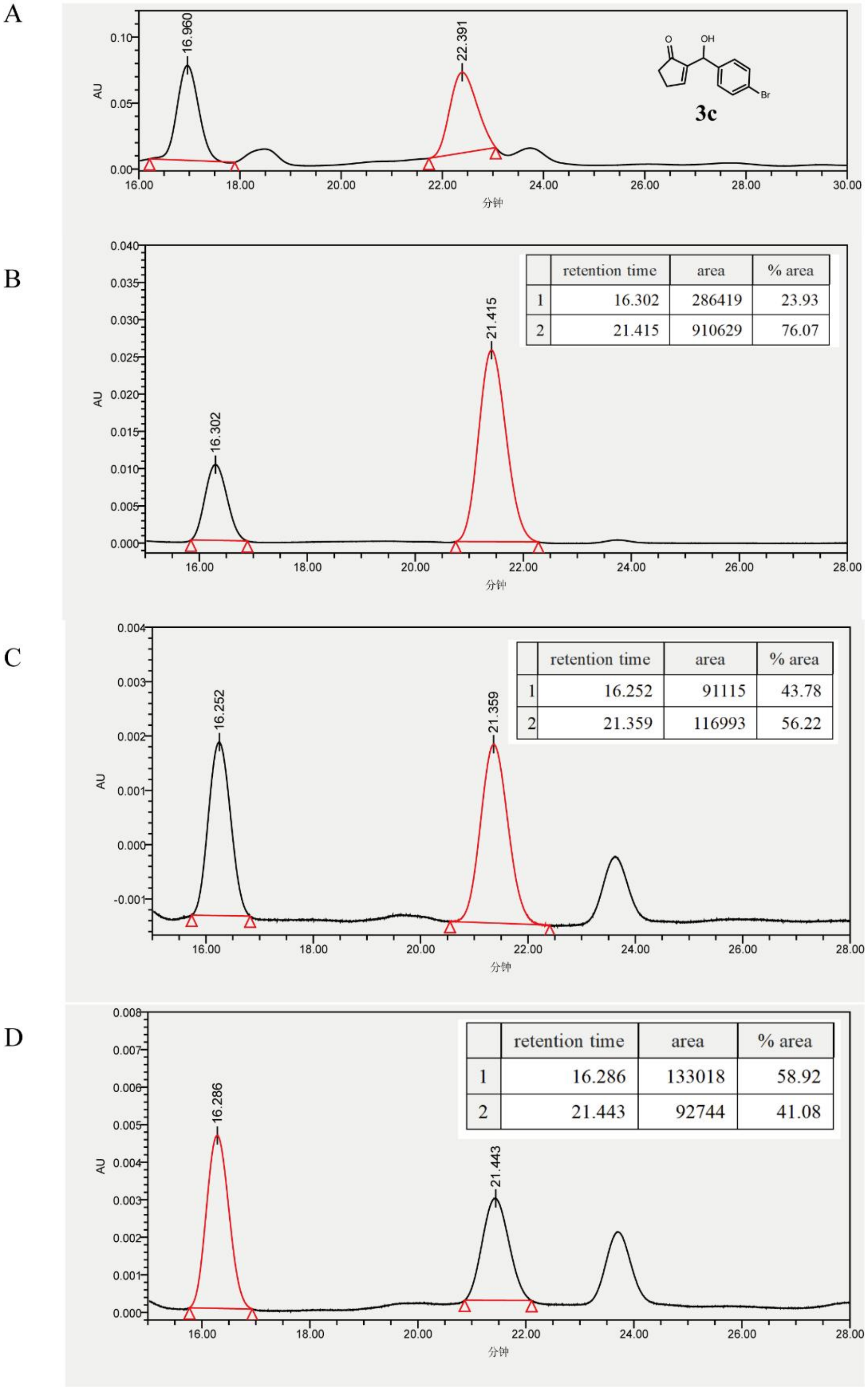

Supplementary Figure 30. Chiral HPLC chromatograms of adducts **3c**. (A) The standard of **3c**. (B) Sample from biotransformation by BH32.14. (C) Sample from biotransformation by *Gk*OYE.11. (D) Sample from biotransformation by *Gk*OYE.13.

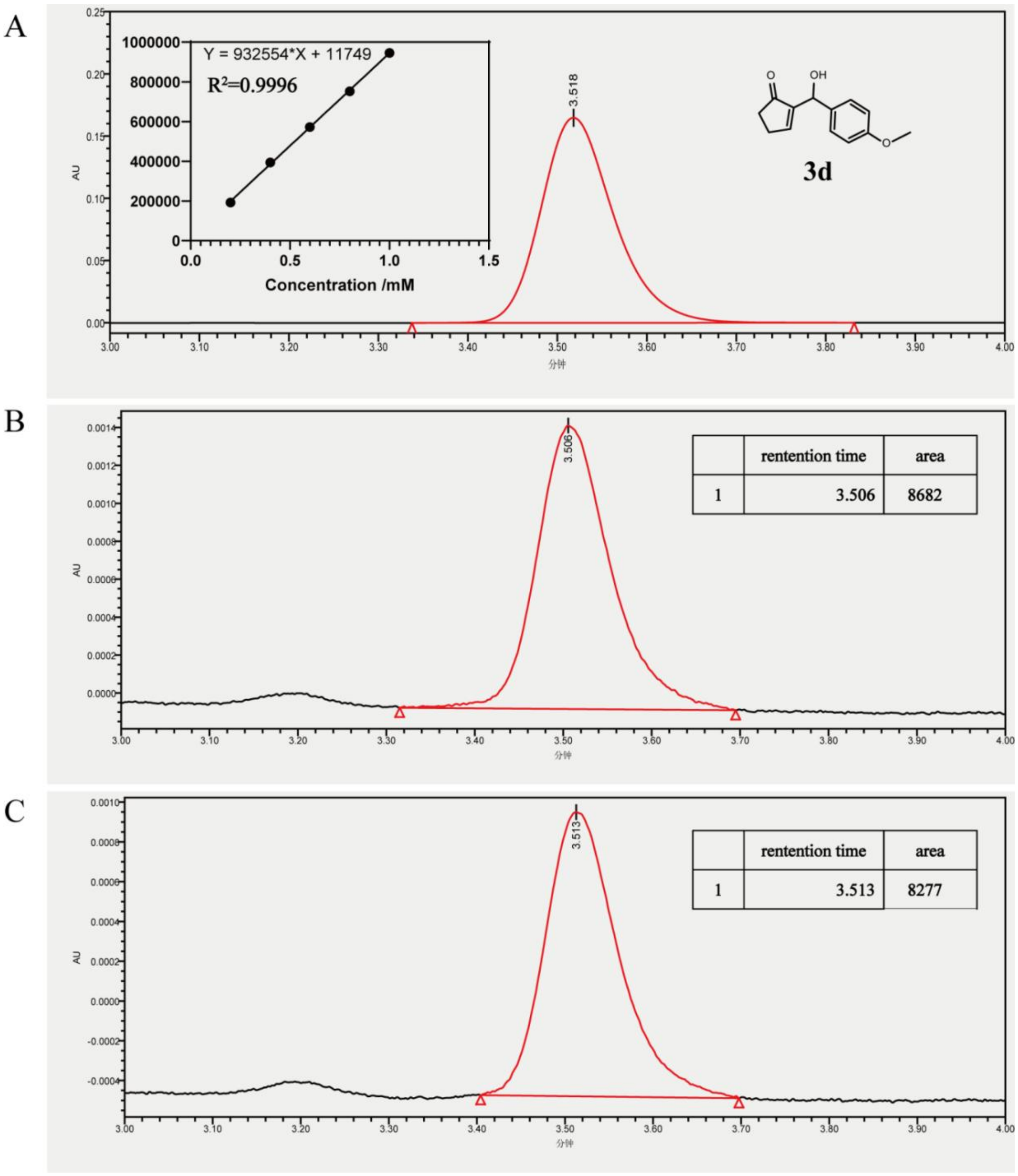

Supplementary Figure 31. HPLC chromatograms of adducts **3d**. (A) The standard of **3d**. (B) Sample from biotransformation by *Gk*OYE.11. (C) Sample from biotransformation by *Gk*OYE.13.

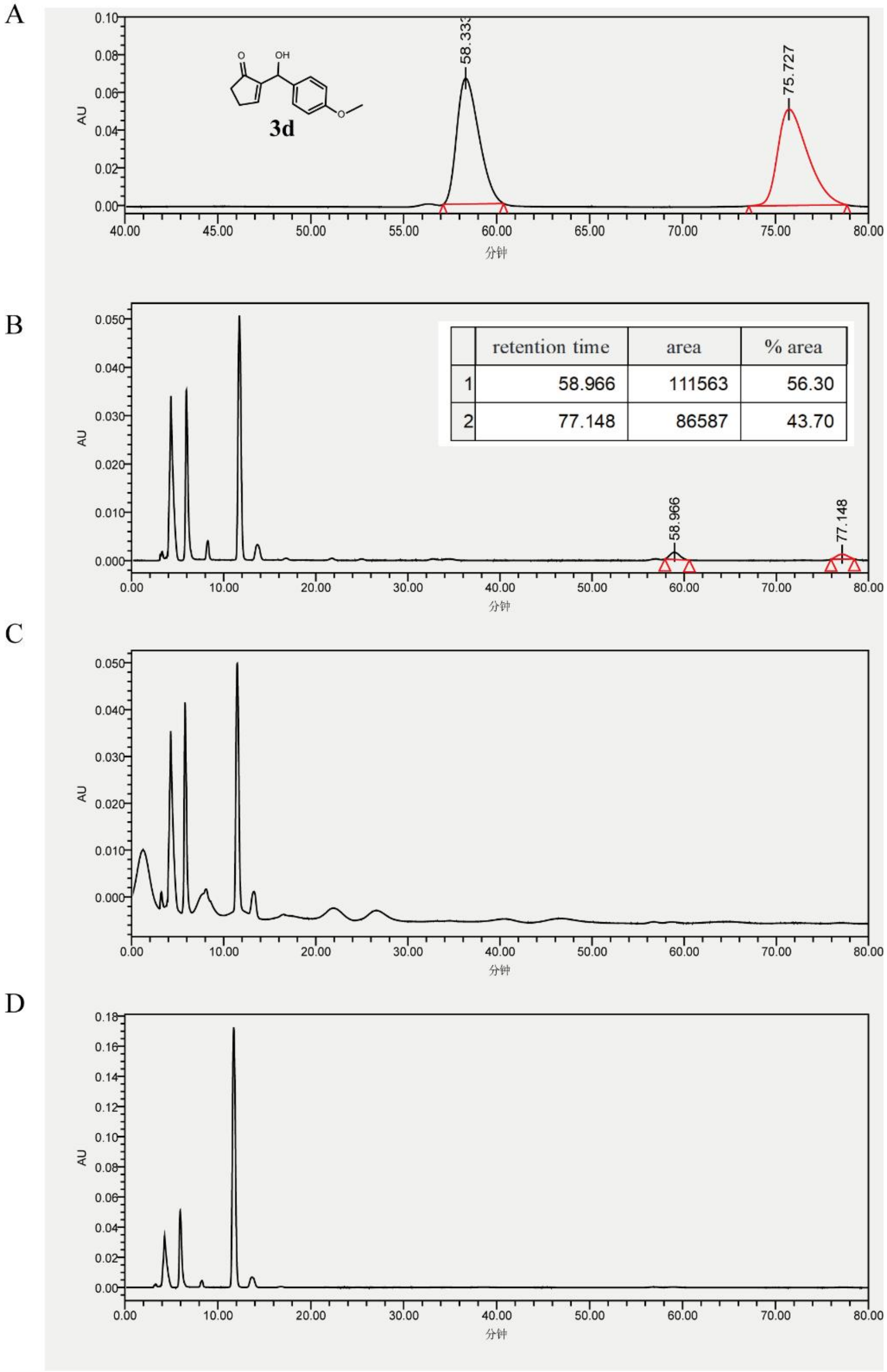

Supplementary Figure 32. Chiral HPLC chromatograms of adducts **3d**. (A) The standard of **3d**. (B) Sample from biotransformation by BH32.14. (C) Sample from biotransformation by *Gk*OYE.11. (D) Sample from biotransformation by *Gk*OYE.13.

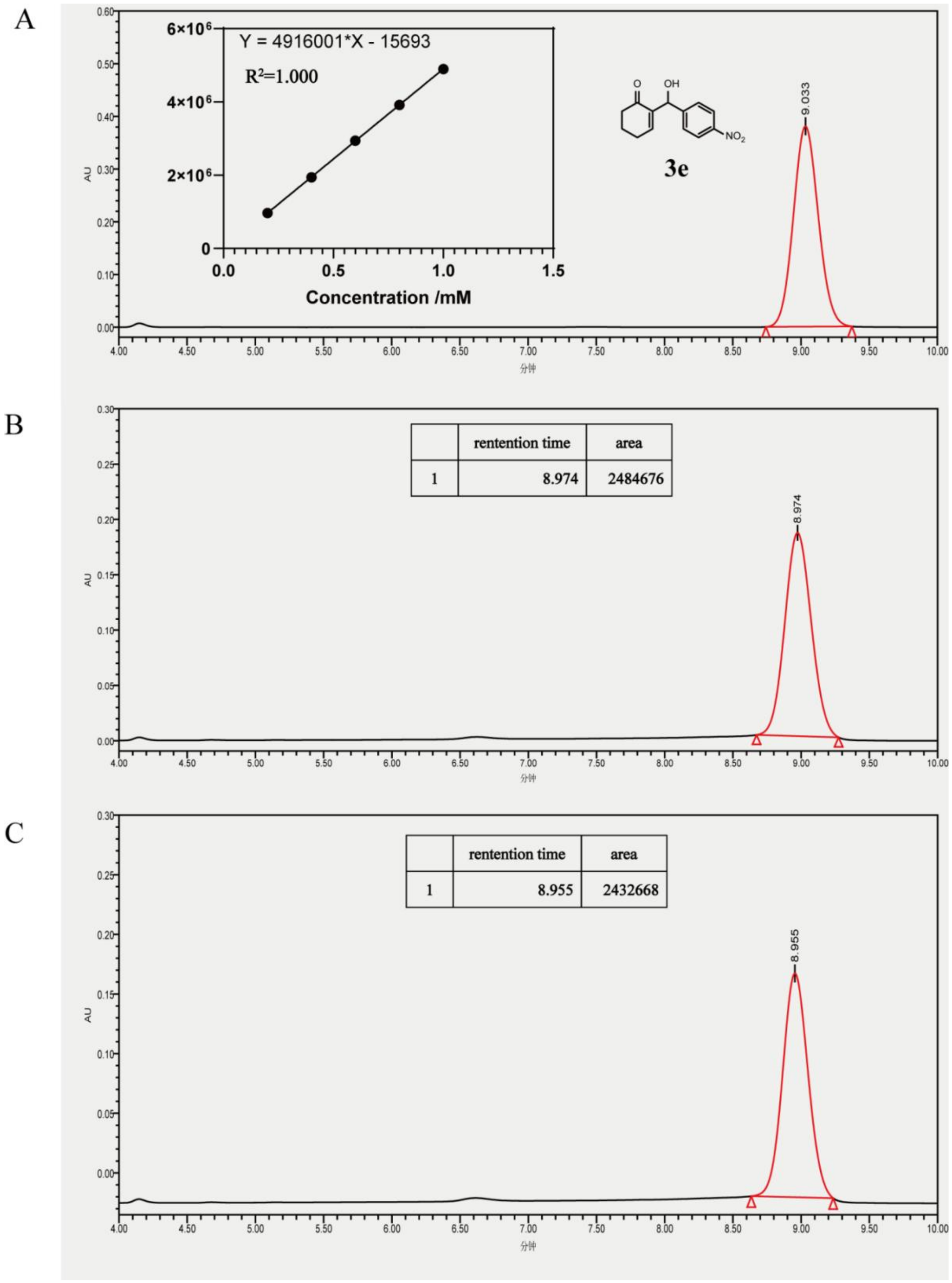

Supplementary Figure 33. HPLC chromatograms of adducts **3e**. (A) The standard of **3e**. (B) Sample from biotransformation by *Gk*OYE.11. (C) Sample from biotransformation by *Gk*OYE.13.

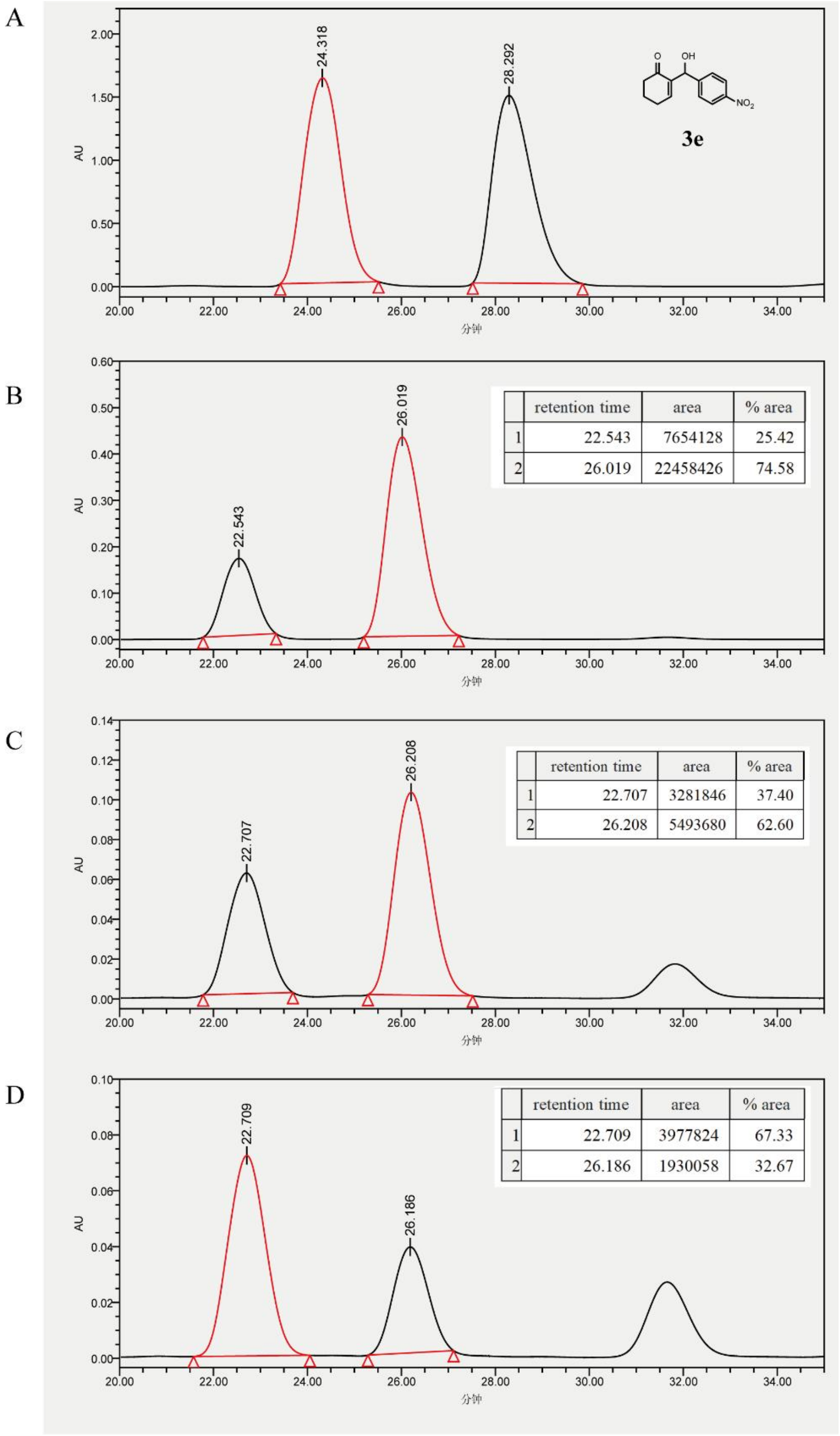

Supplementary Figure 34. Chiral HPLC chromatograms of adducts **3e**. (A) The standard of **3e**. (B) Sample from biotransformation by BH32.14. (C) Sample from biotransformation by *Gk*OYE.11. (D) Sample from biotransformation by *Gk*OYE.13.

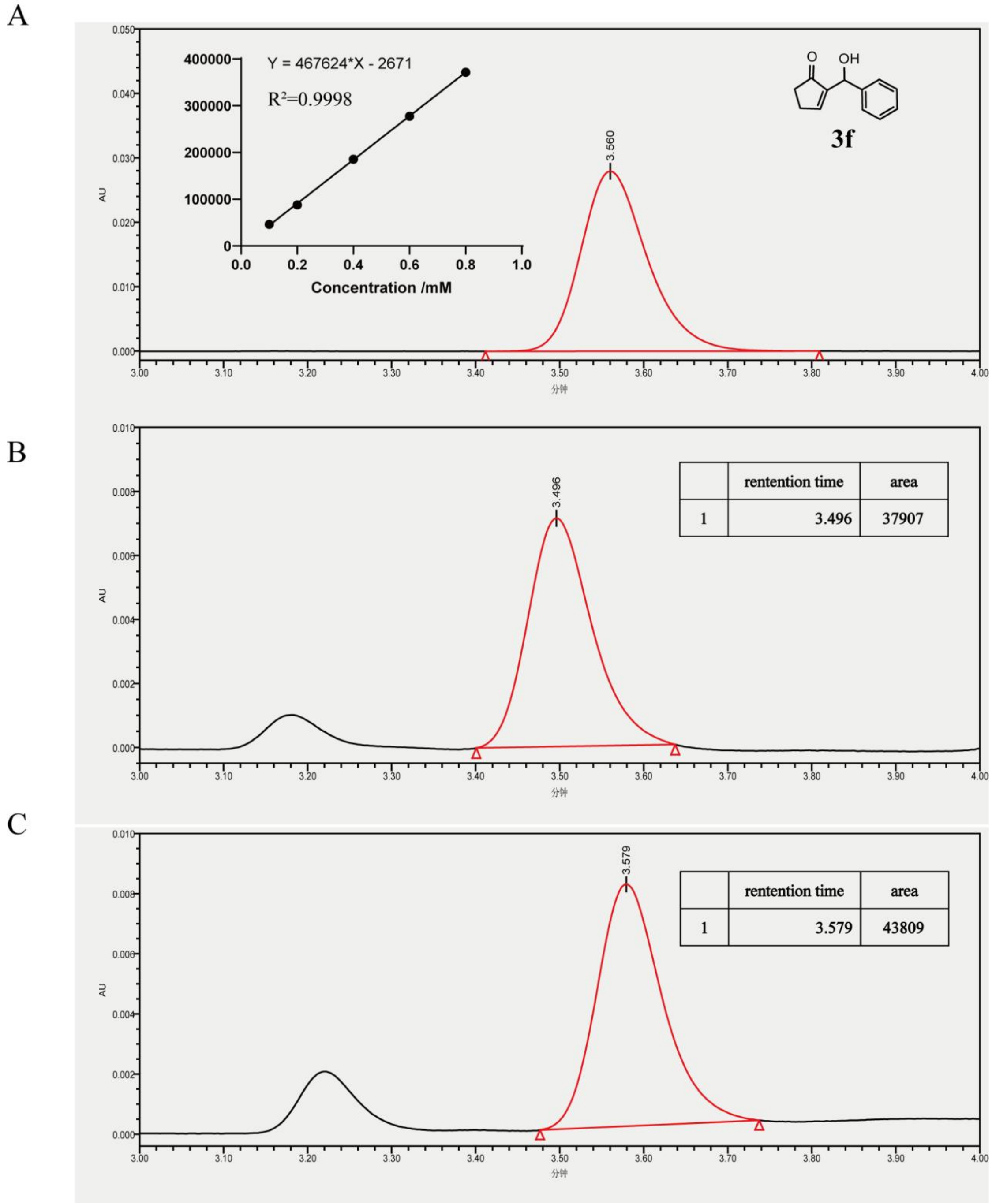

Supplementary Figure 35. HPLC chromatograms of adducts **3f**. (A) The standard of **3f**. (B) Sample from biotransformation by *Gk*OYE.11. (C) Sample from biotransformation by *Gk*OYE.13.

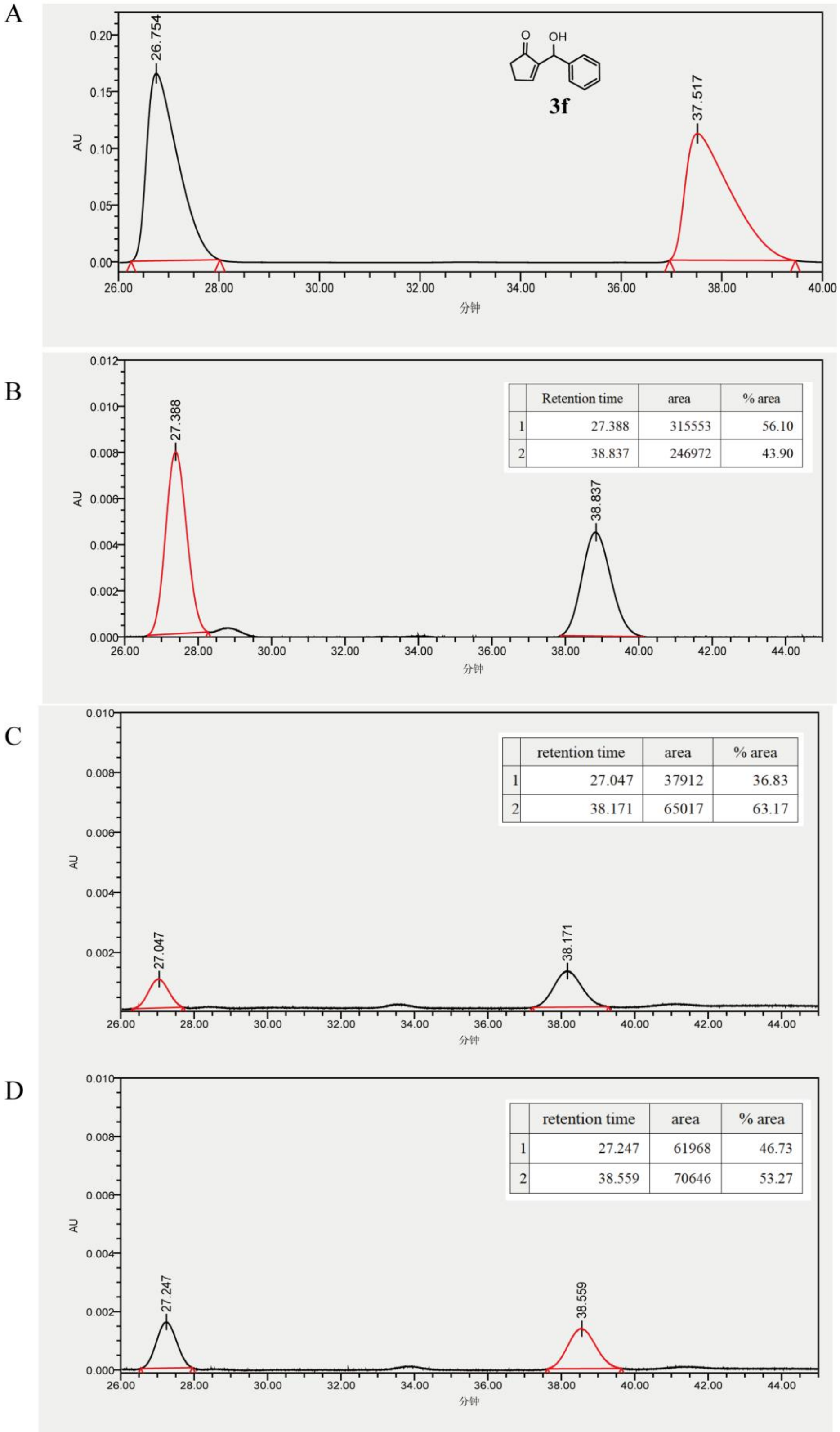

Supplementary Figure 36. Chiral HPLC chromatograms of adducts **3f**. (A) The standard of **3f**. (B) Sample from biotransformation by BH32.14. (C) Sample from biotransformation by *Gk*OYE.11. (D) Sample from biotransformation by *Gk*OYE.13.

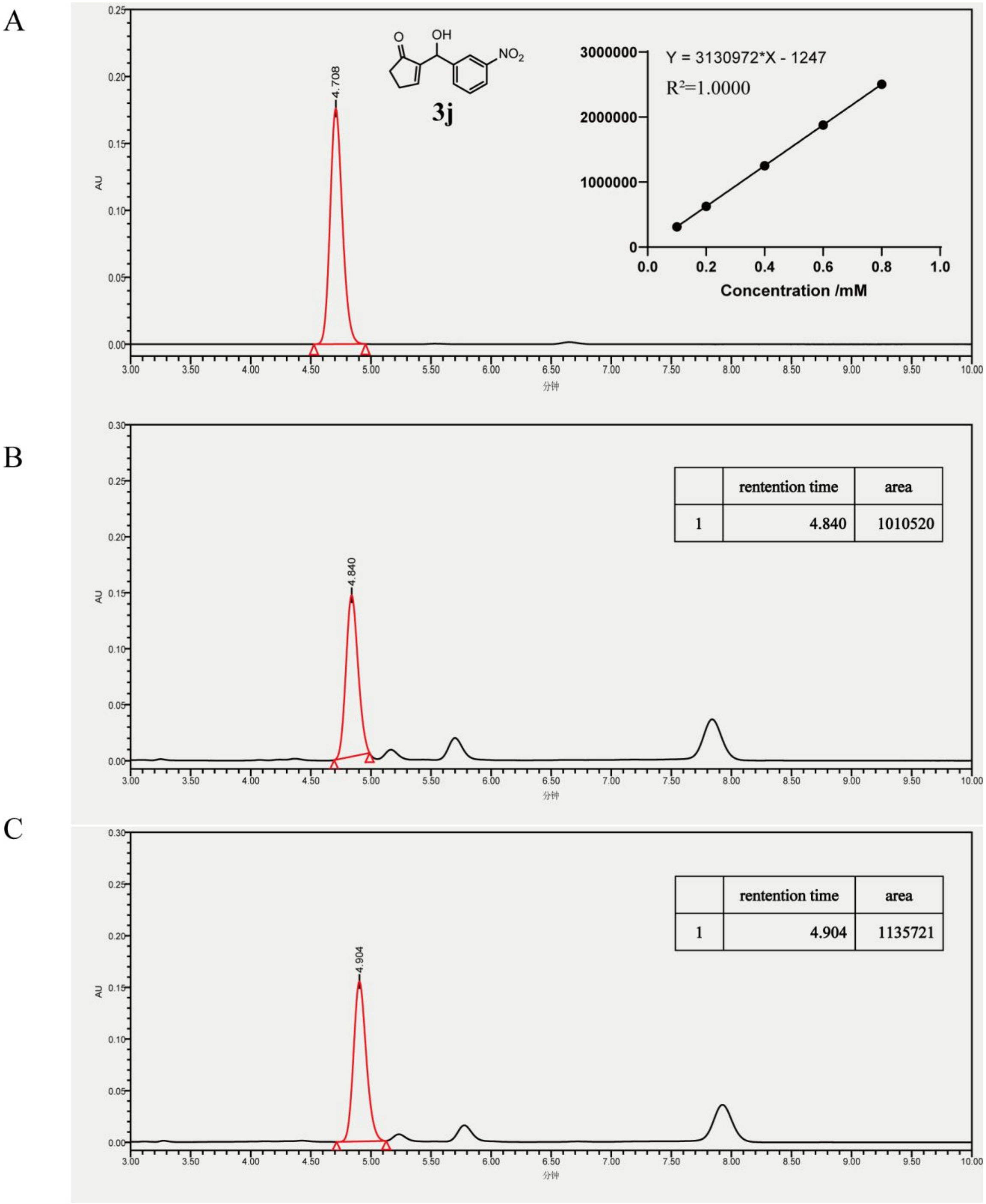

Supplementary Figure 37. HPLC chromatograms of adducts **3j**. (A) The standard of **3j**. (B) Sample from biotransformation by *Gk*OYE.11. (C) Sample from biotransformation by *Gk*OYE.13.

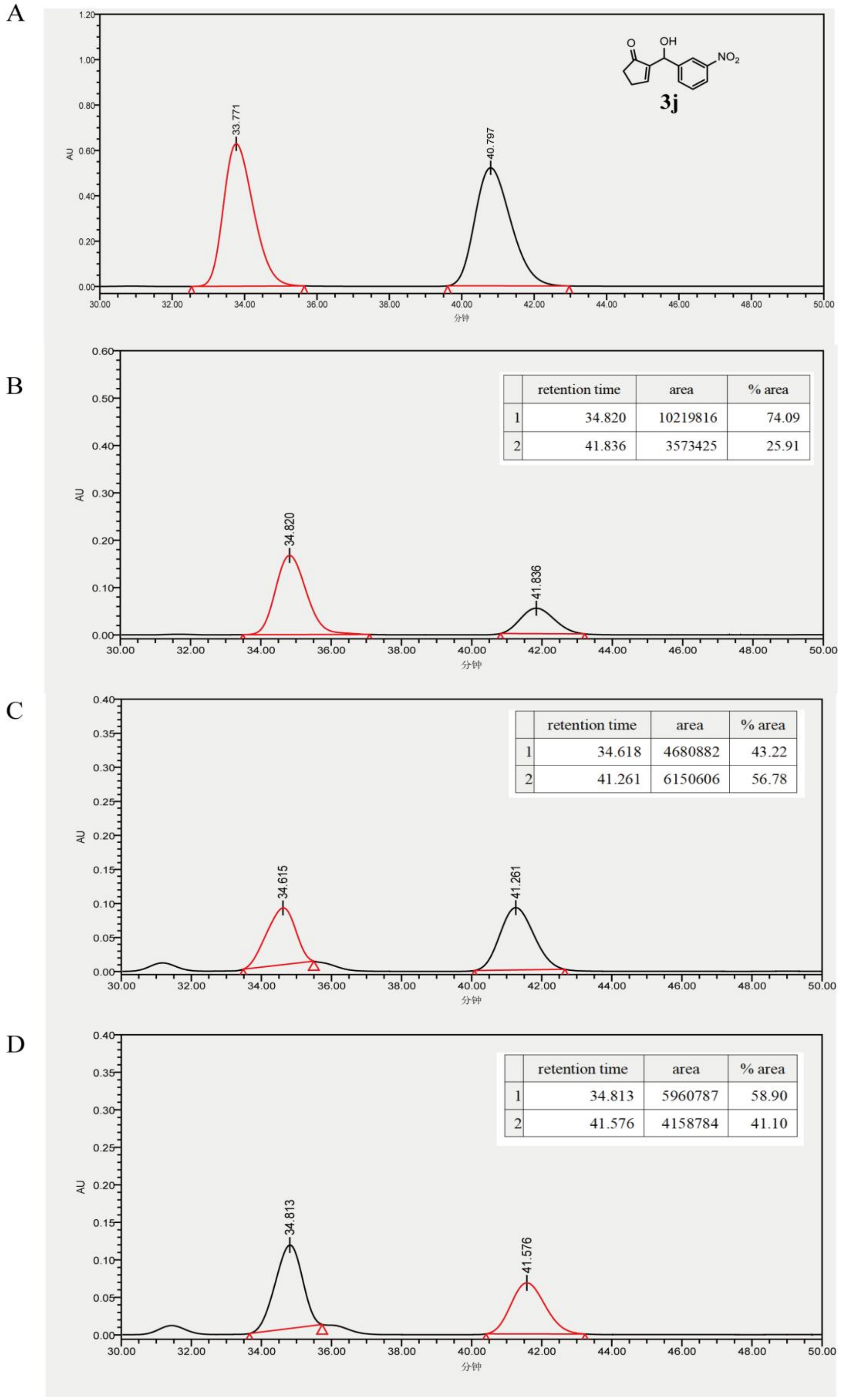

(B)

	retention time	area	% area
1	34.820	10219816	74.09
2	41.836	3573425	25.91

(C)

	retention time	area	% area
1	34.618	4680882	43.22
2	41.261	6150606	56.78

(D)

	retention time	area	% area
1	34.813	5960787	58.90
2	41.576	4158784	41.10

Supplementary Figure 38. Chiral HPLC chromatograms of adducts **3j**. (A) The standard of **3j**. (B) Sample from biotransformation by BH32.14. (C) Sample from biotransformation by *Gk*OYE.11. (D) Sample from biotransformation by *Gk*OYE.13.

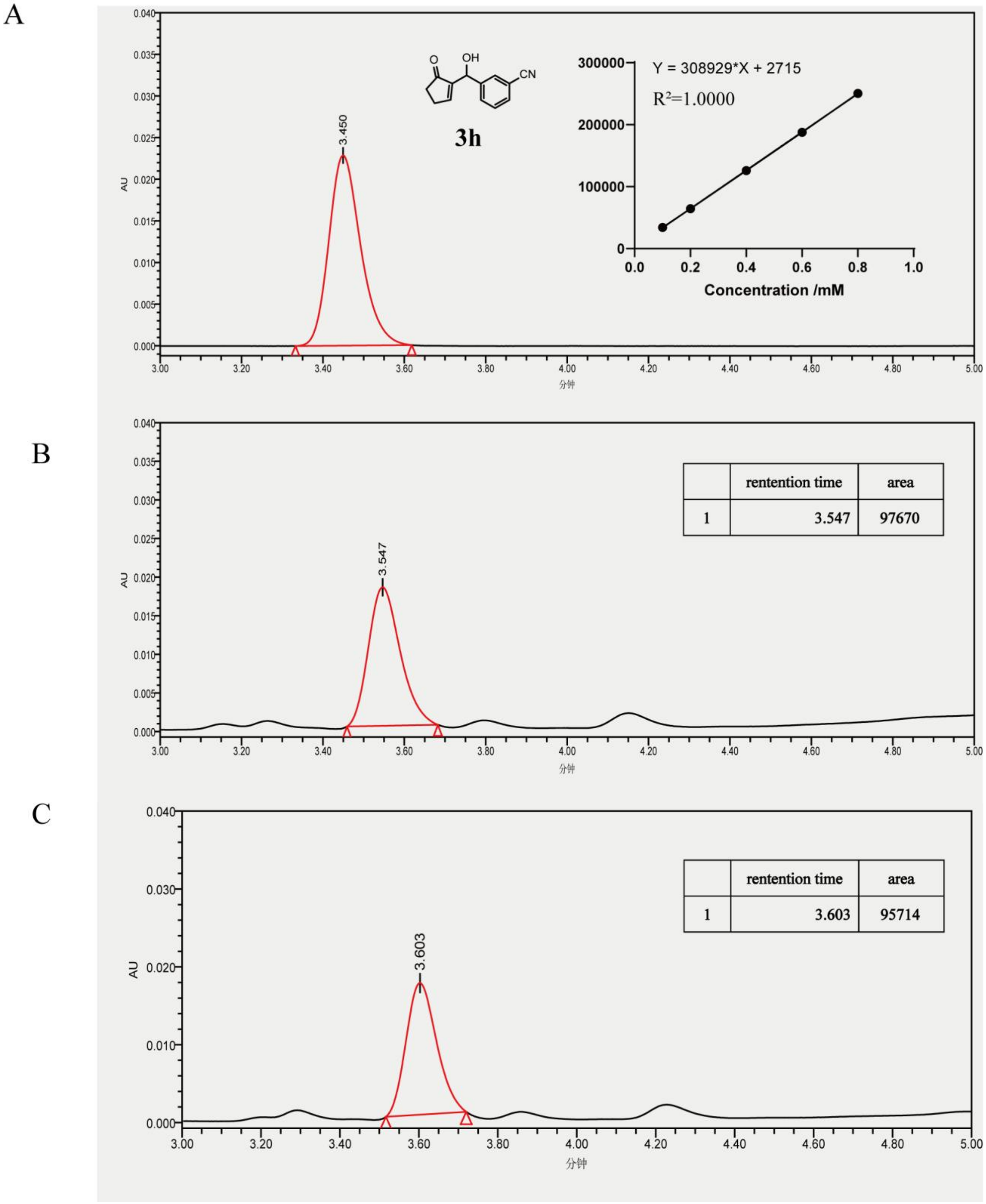

Supplementary Figure 39. HPLC chromatograms of adducts **3h**. (A) The standard of **3h**. (B) Sample from biotransformation by *Gk*OYE.11. (C) Sample from biotransformation by *Gk*OYE.13.

A

B

C

D

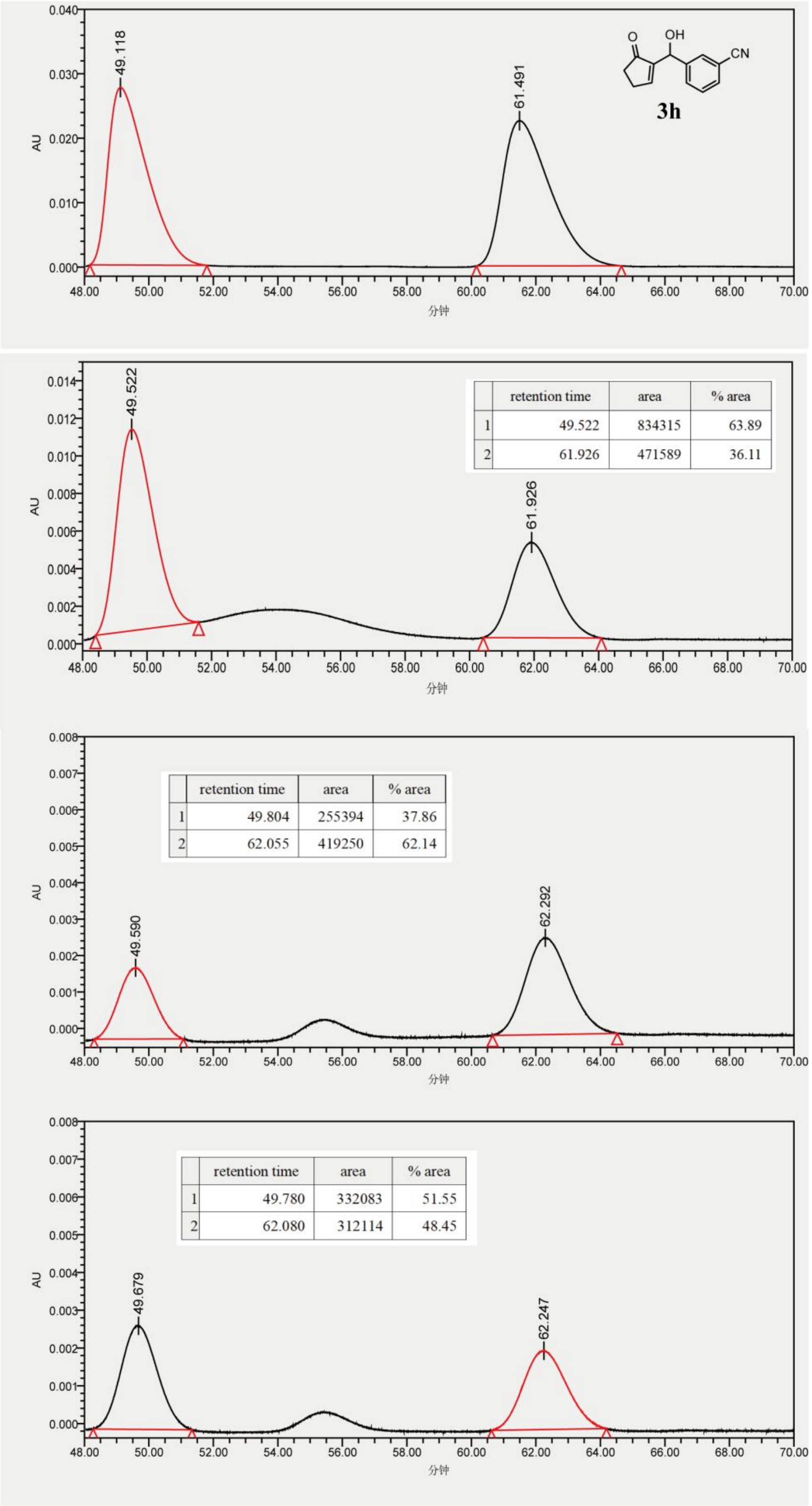

Supplementary Figure 40. Chiral HPLC chromatograms of adducts **3h**. (A) The standard of **3h**. (B) Sample from biotransformation by BH32.14. (C) Sample from biotransformation by *Gk*OYE.11. (D) Sample from biotransformation by *Gk*OYE.13.

A

B

C

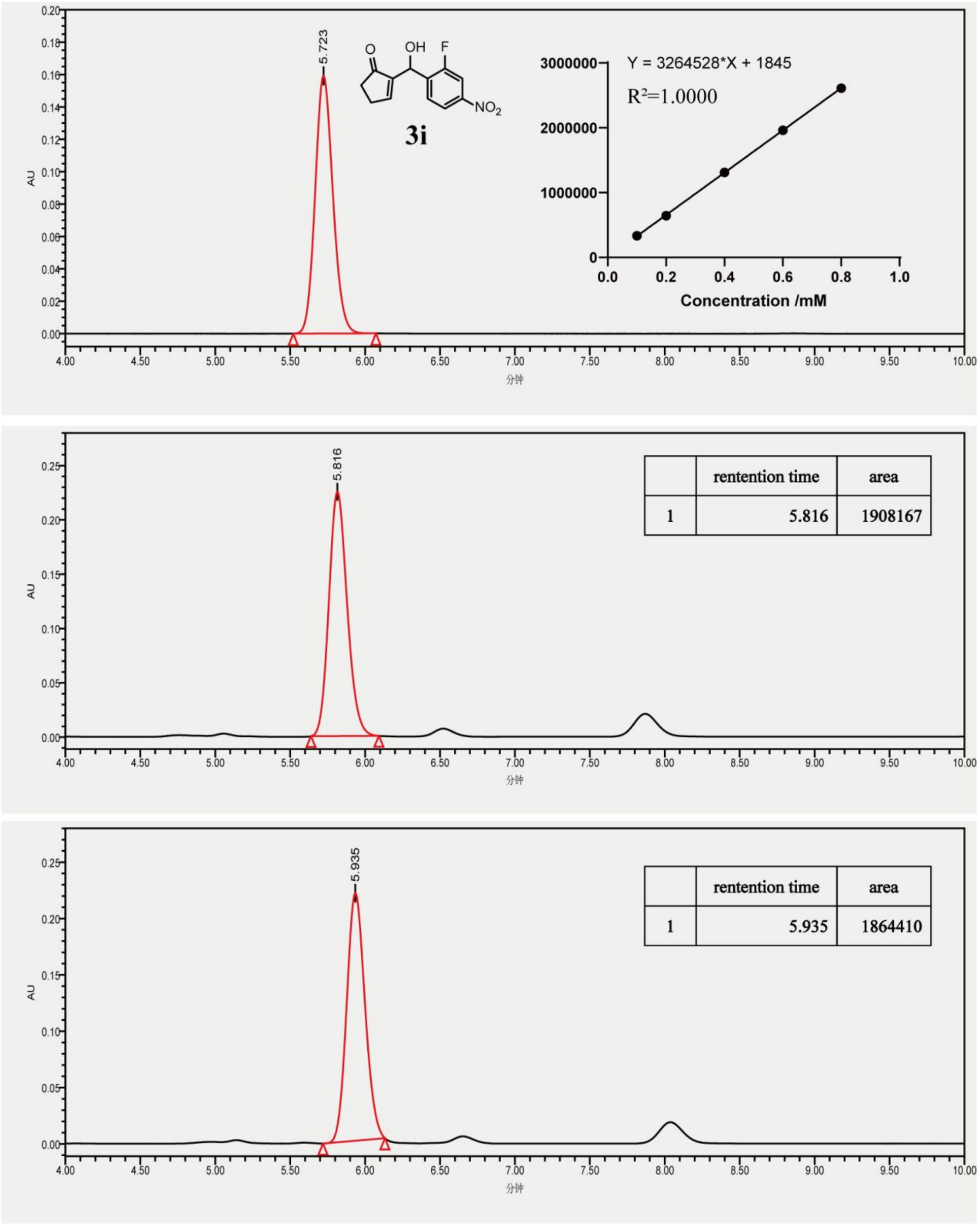

Supplementary Figure 41. HPLC chromatograms of adducts **3i**. (A) The standard of **3i**. (B) Sample from biotransformation by *Gk*OYE.11. (C) Sample from biotransformation by *Gk*OYE.13.

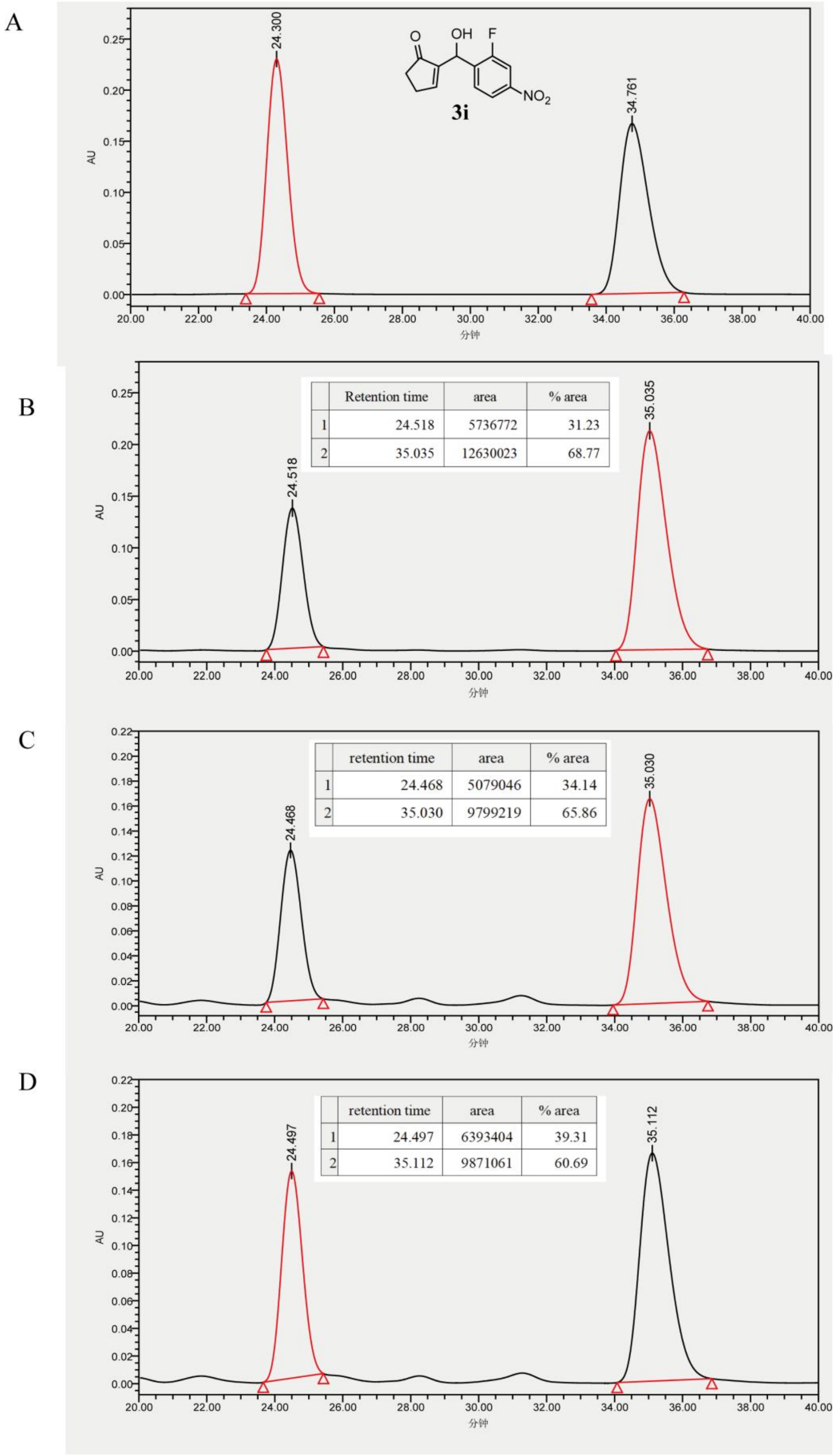

Supplementary Figure 42. Chiral HPLC chromatograms of adducts **3i**. (A) The standard of **3i**. (B) Sample from biotransformation by BH32.14. (C) Sample from biotransformation by *Gk*OYE.11. (D) Sample from biotransformation by *Gk*OYE.13.

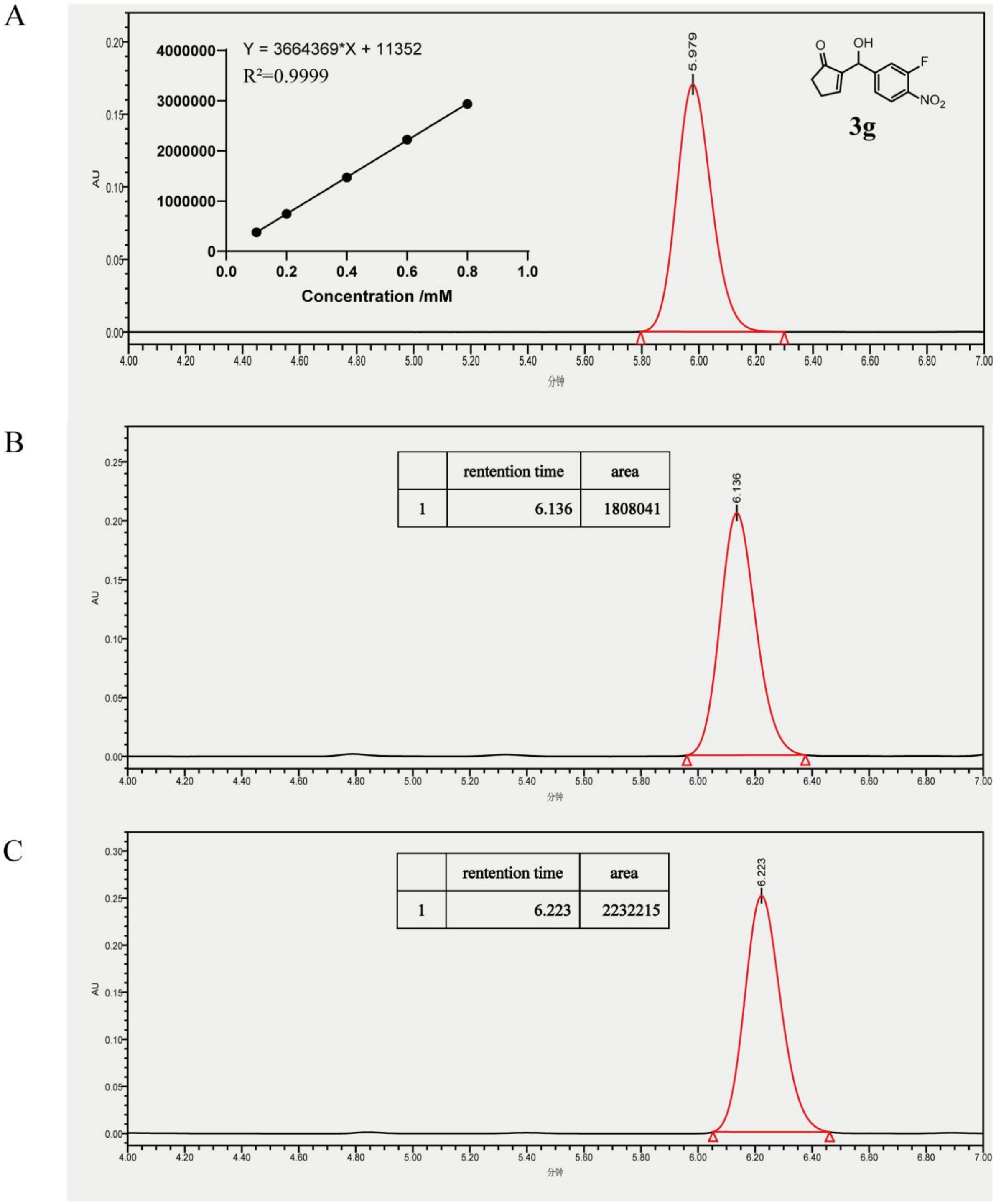

Supplementary Figure 43. HPLC chromatograms of adducts **3g**. (A) The standard of **3g**. (B) Sample from biotransformation by *Gk*OYE.11. (C) Sample from biotransformation by *Gk*OYE.13.

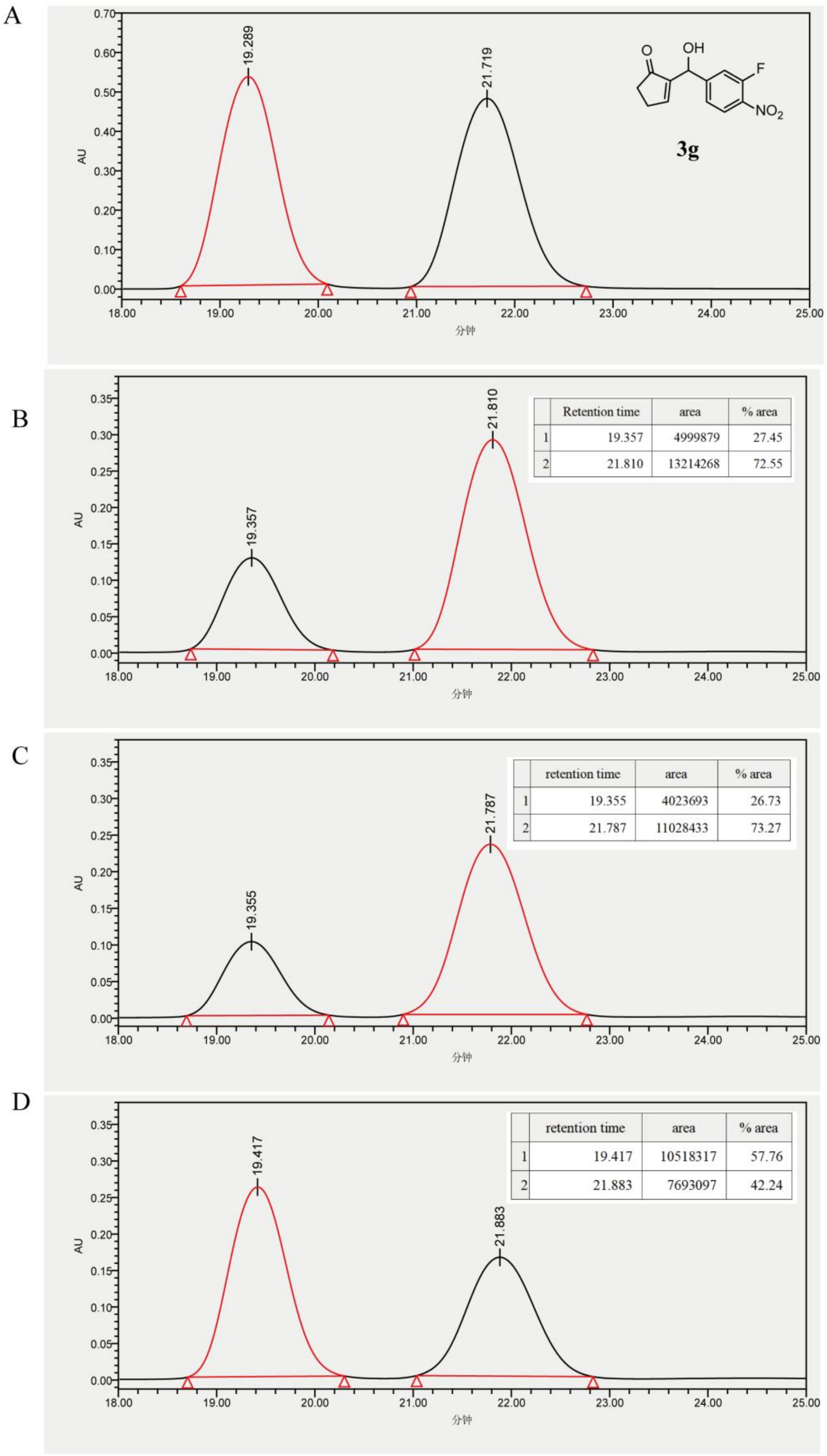

Supplementary Figure 44. Chiral HPLC chromatograms of adducts **3g**. (A) The standard of **3g**. (B) Sample from biotransformation by BH32.14. (C) Sample from biotransformation by *Gk*OYE.11. (D) Sample from biotransformation by *Gk*OYE.13.

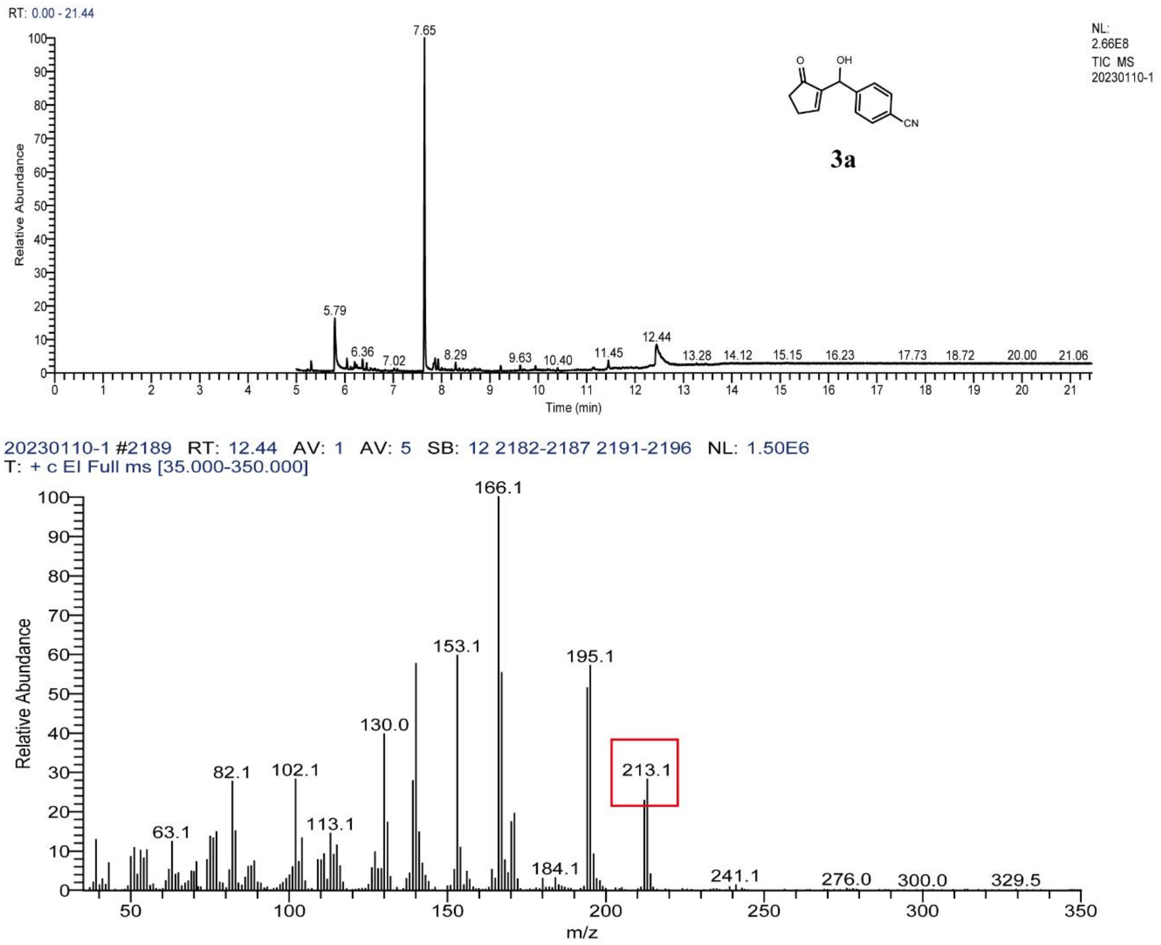

Supplementary Figure 45. GC-MS analysis of **3a.**

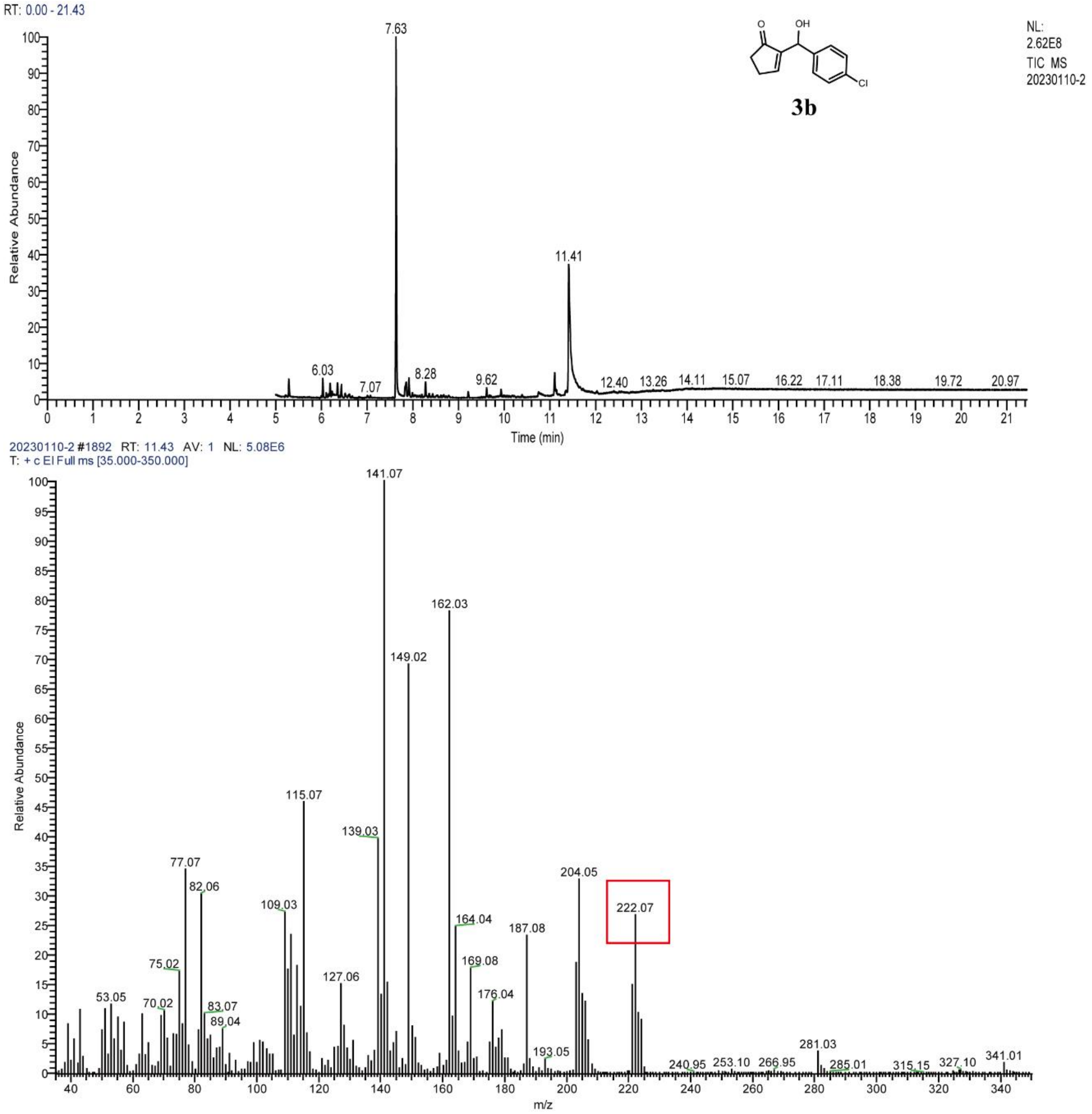

Supplementary Figure 46. GC-MS analysis of **3b.**

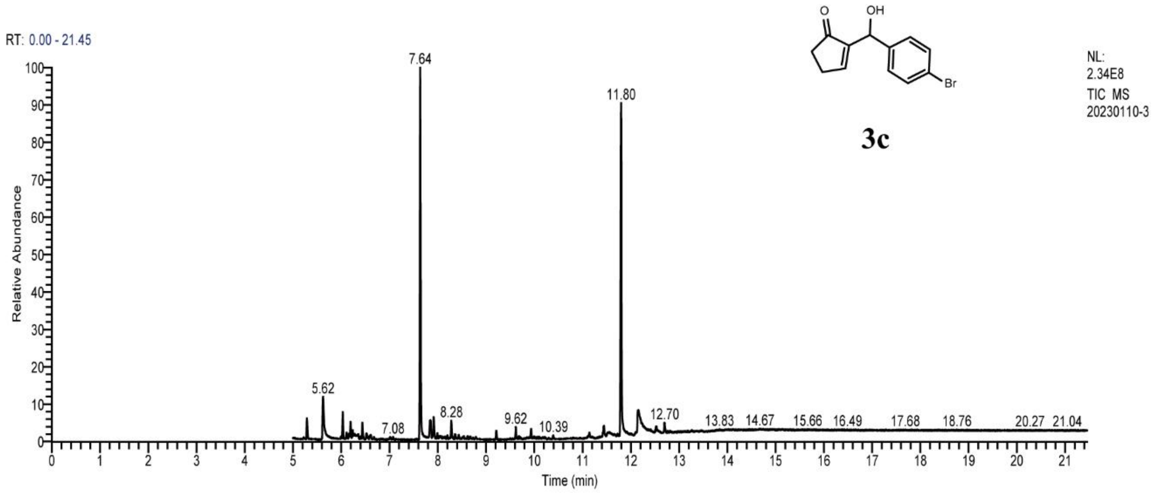

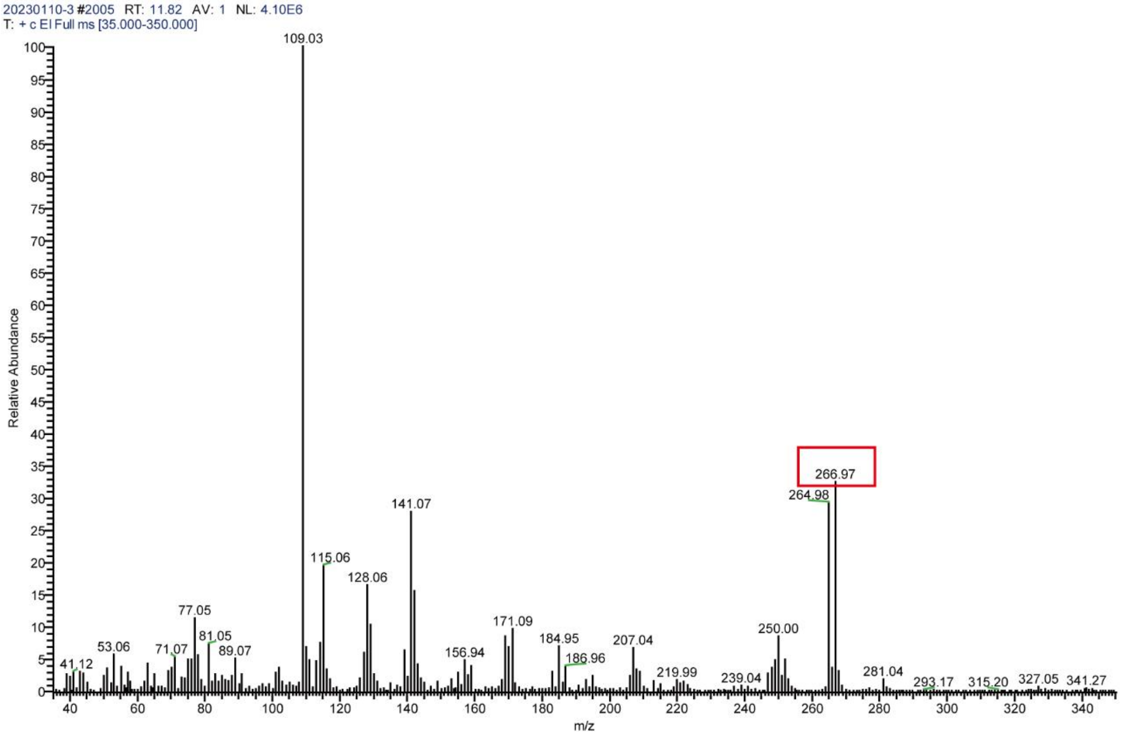

Supplementary Figure 47. GC-MS analysis of **3c.**

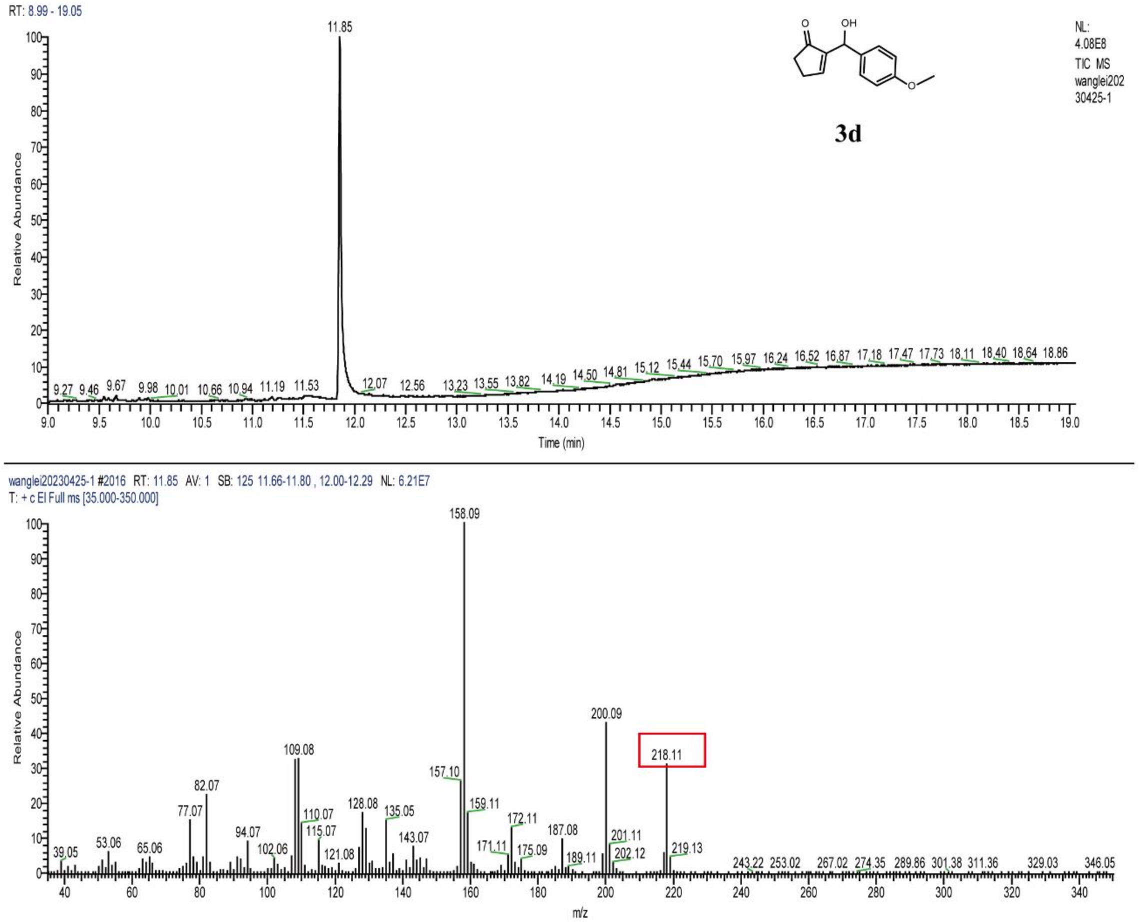

Supplementary Figure 48. GC-MS analysis of **3d.**

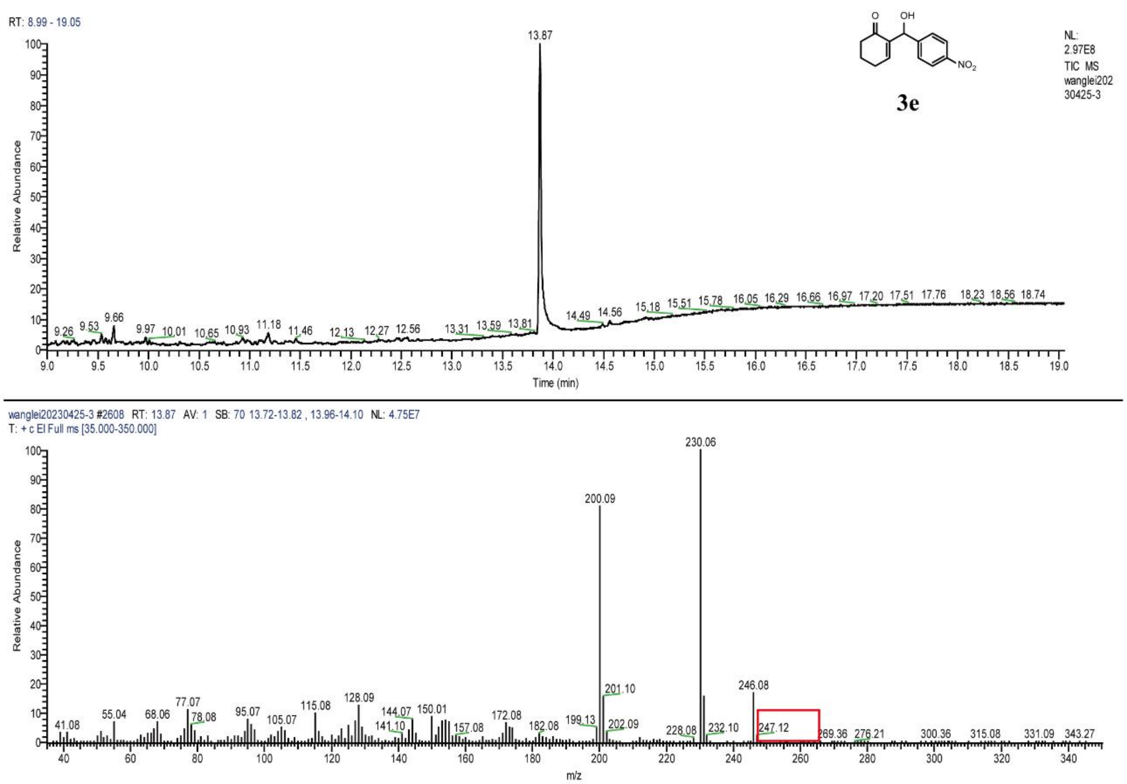

Supplementary Figure 49. GC-MS analysis of **3e.**

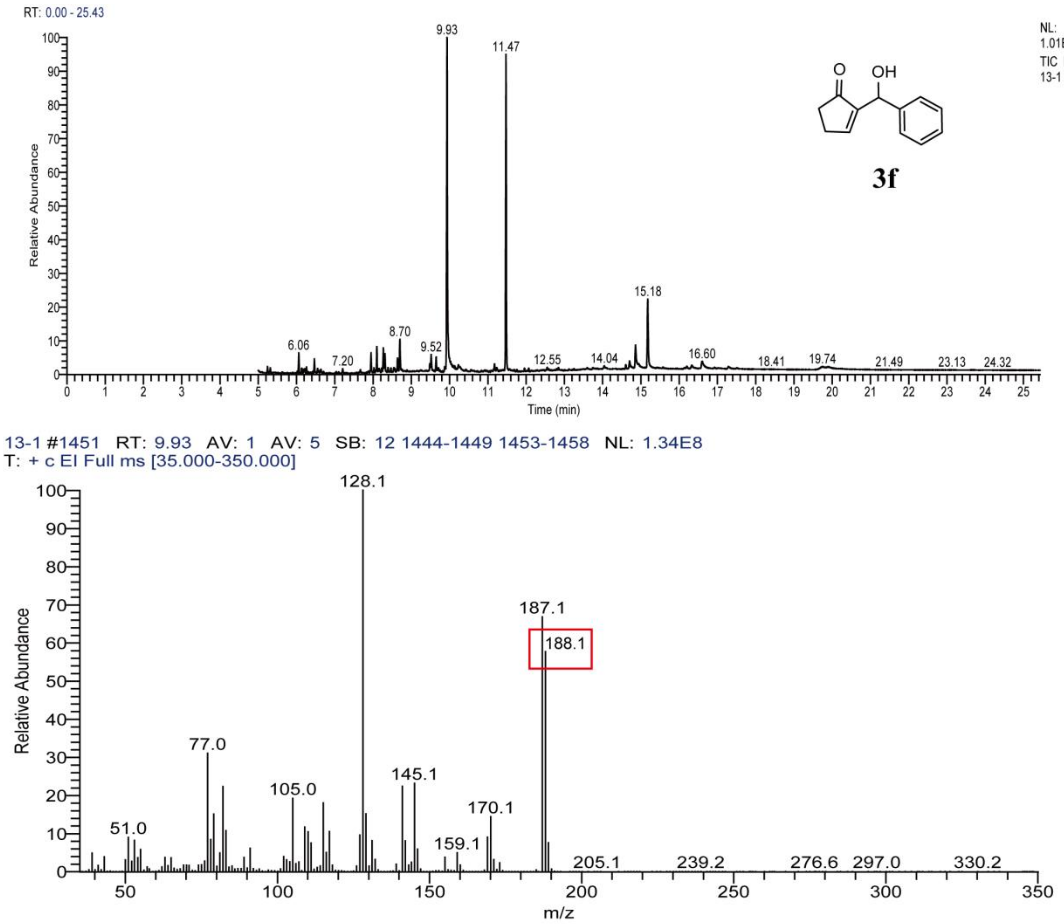

Supplementary Figure 50. GC-MS analysis of **3f.**

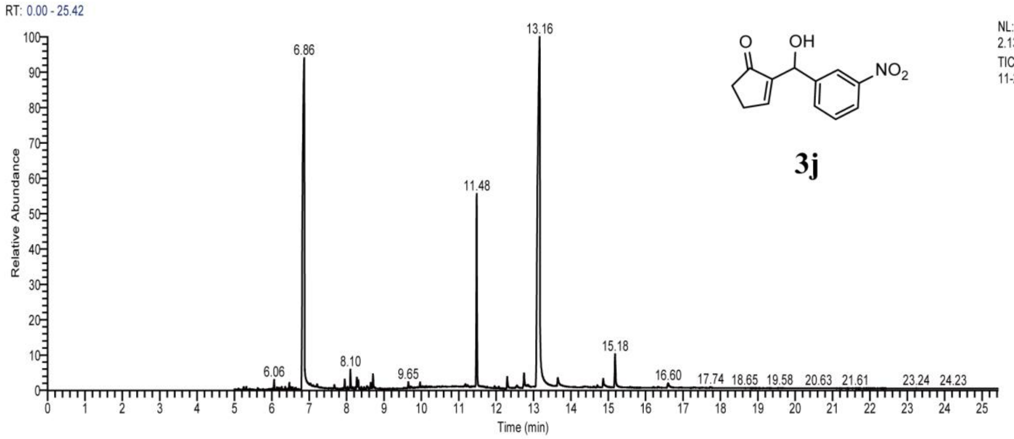

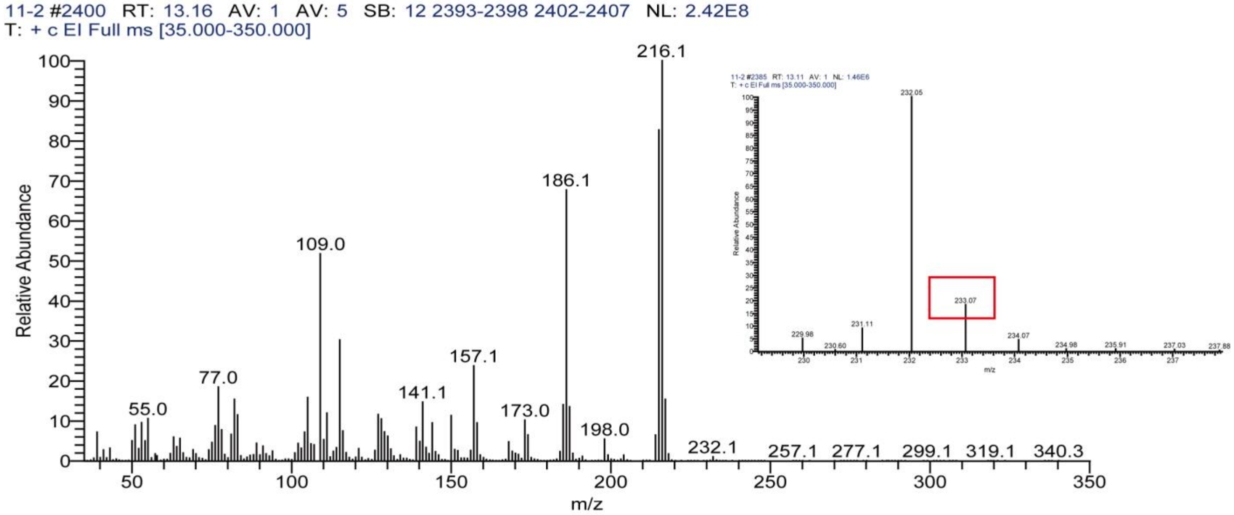

Supplementary Figure 51. GC-MS analysis of **3j.**

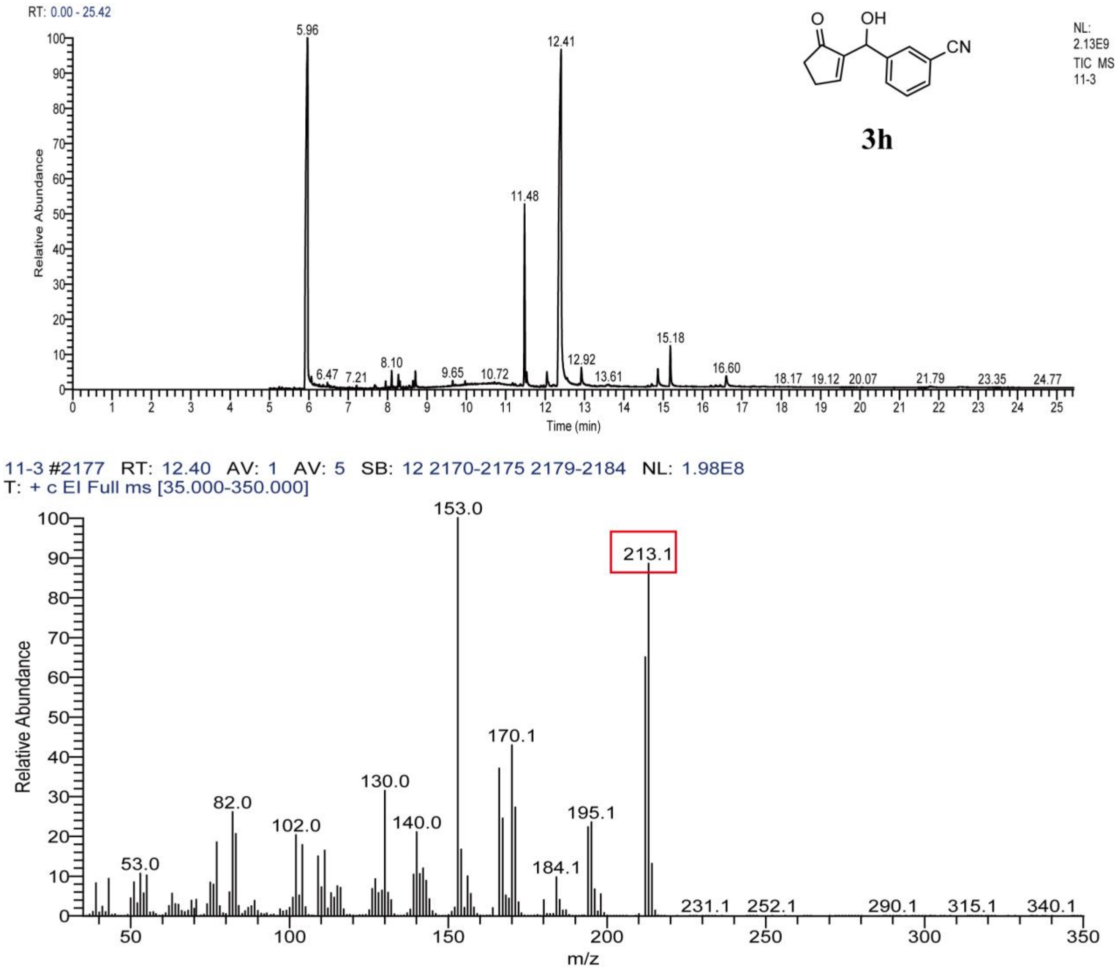

Supplementary Figure 52. GC-MS analysis of **3h.**

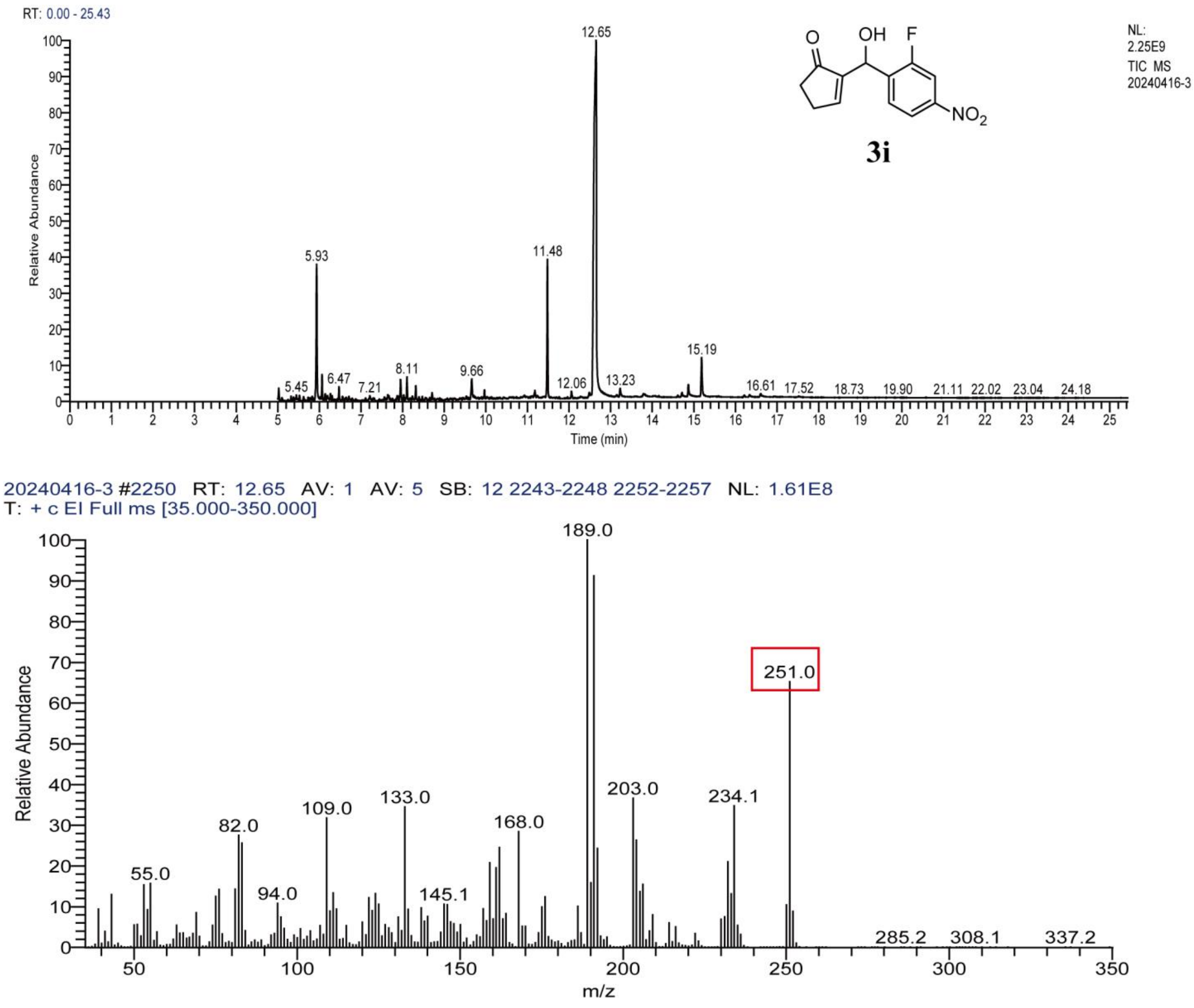

Supplementary Figure 53. GC-MS analysis of **3i.**

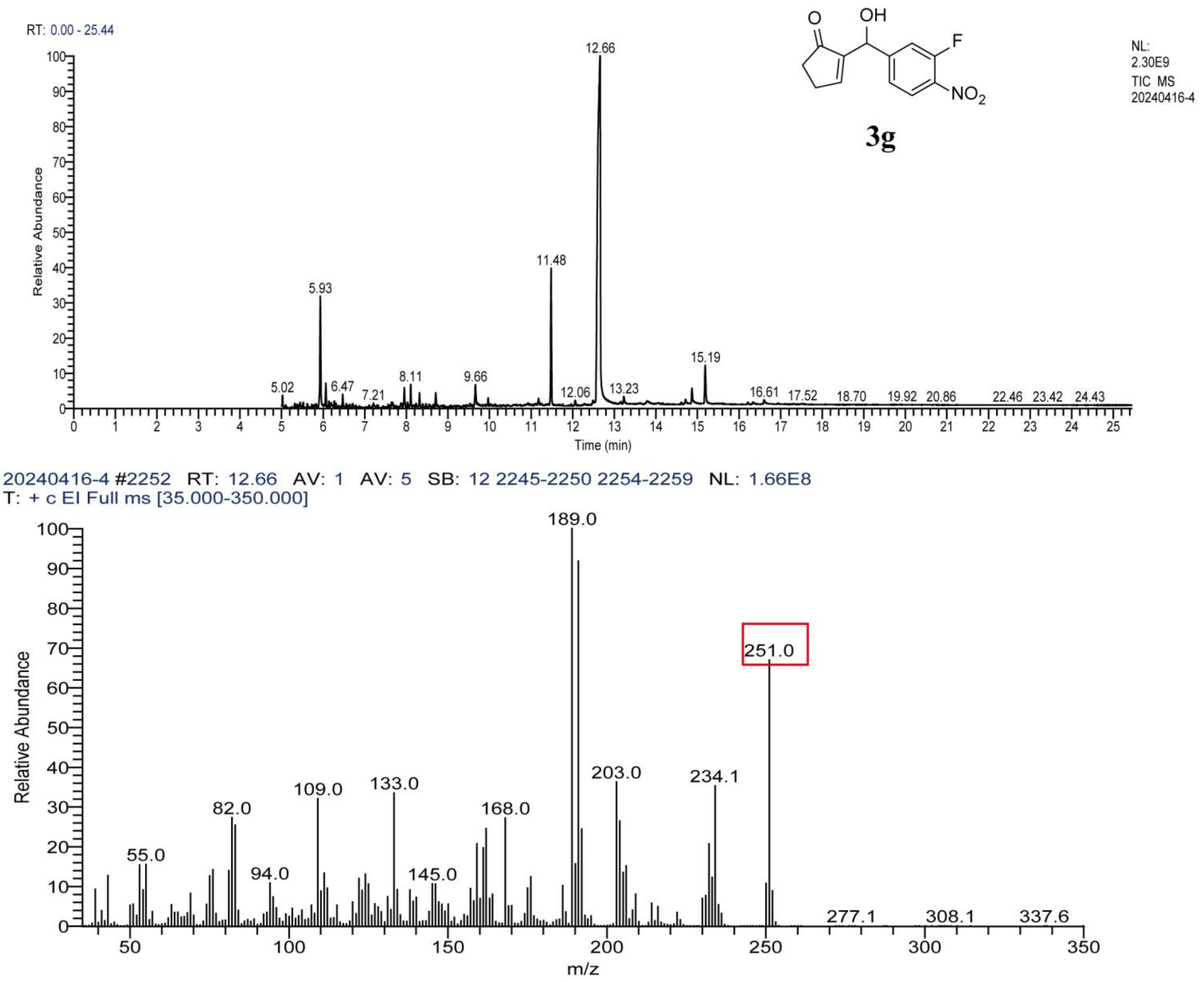

Supplementary Figure 54. GC-MS analysis of **3g.**

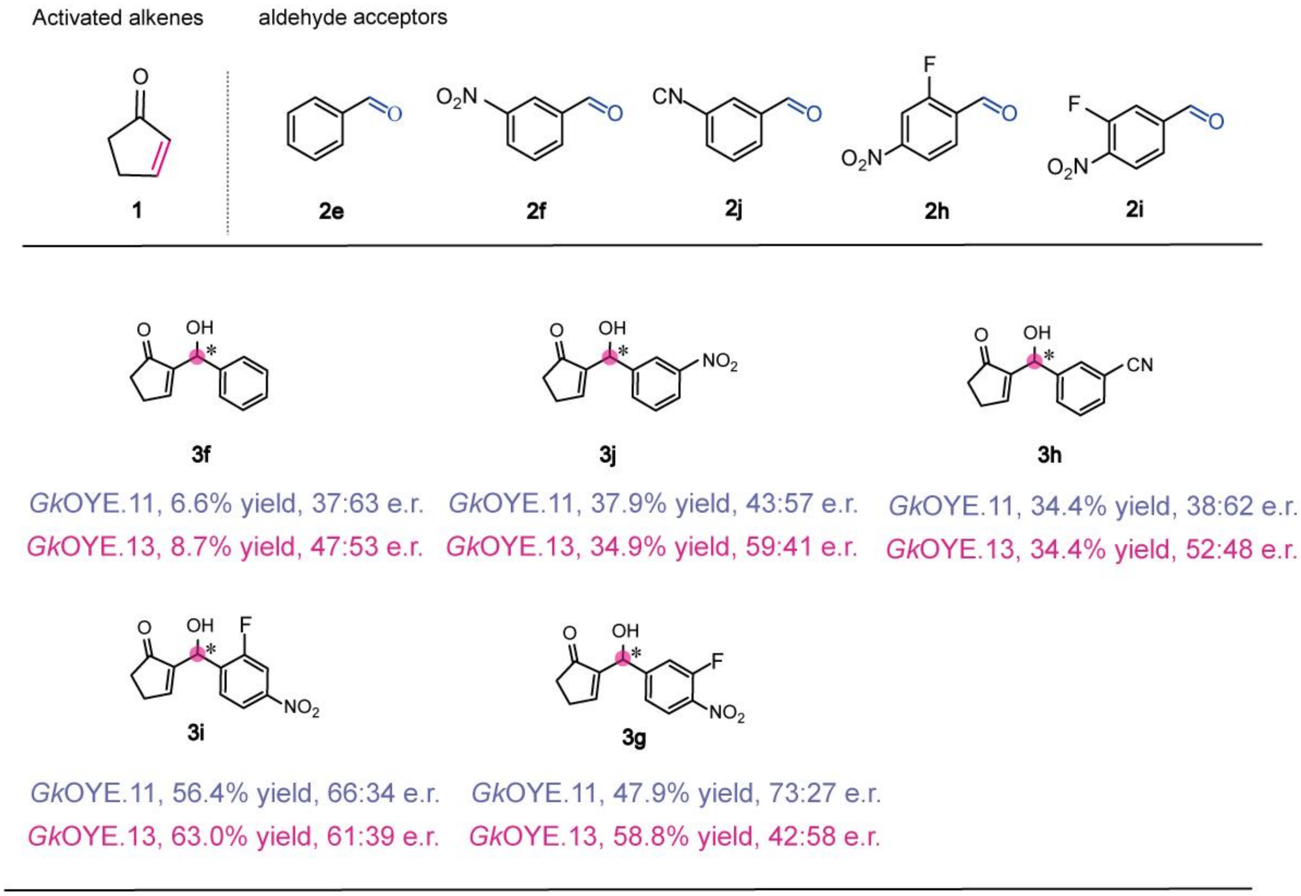

Supplementary Figure 55. The supplement of substrate scope of *Gk*OYE.11 and *Gk*OYE.13. Biotransformations were performed using aldehyde (1 mM), activated alkene (5 mM) and catalyst (100 μM). The detailed information of results and determination methods for the yield and enantiomeric ratio of the MBH adducts were shown in Supplementary materials.

Supplementary Tables

Supplementary Table 1. Comparison the pockets volume of different mutants.

Strains	Volume of pockets (Å^3)
*Gk*OYE.4	566.85
*Gk*OYE.5	553.98
*Gk*OYE.6	501.82
*Gk*OYE.7	597.95

Supplementary Table 2. Data collection and refinement statistics of *apo-Gk*OYE.8 crystal structure.

	Apo-*Gk*OYE.8
PDB code	8X0J
Data collection	
Space group	I 21 21 21
Cell dimenesion	
a, b, c (Å)	86.06, 143.57, 155.47
α, β, γ (Å)	90.00, 90.00, 90.00
Resolution (Å)	45.74 - 3.11 Å
R_{merge}	0.17 (0.49)
R_{pim}	0.146 (0.406)
CC1/2	0.967(0.726)
I/σ(I)	5.8 (2.1)
% Data completeness(in resolution range)	98.5(45.74-3.11)
Refinement	
No. reflections	17753
Rwork/Rfree	0.2151/0.2648
Total number of atoms	5314
Wilson B-factor (Å^2)	39.8
R.m.s.deviations	
bond lengths (Å)	0.0063
bond angles (°)	1.4525
Ramachandran outliers (%)	0.00
Ramachandran favored (%)	96.15

Supplementary Note Genetic constructions and optimized sequences

The *Gk*OYE gene (from *Geobacillus kaustophilus*) was synthesized by GenScript (Piscataway, NJ) and codon optimized for *E. coli*. The sequence was then incorporated into the pET-28a plasmid using the *Nde*I and *Xho*I restriction sites, following standard molecular cloning procedures. The gene and protein sequences are shown in Supplementary Data 1.

The XenA gene (from *Pseudomonas putida*) was synthesized by GenScript (Piscataway, NJ) and codon optimized for *E. coli*. The sequence was then incorporated into the pET-28a plasmid using the *Nde*I and *Xho*I restriction sites, following standard molecular cloning procedures. The gene and protein sequences are shown in Supplementary Data 1.

The NemA gene (from *Escherichia coli str. K-12*) was synthesized by GenScript (Piscataway, NJ) and codon optimized for *E. coli*. The sequence was then incorporated into the pET-28a plasmid using the *Nde*I and *Xho*I restriction sites, following standard molecular cloning procedures. The gene and protein sequences are shown in Supplementary Data 1.

The GluER gene (from *Gluconobacter oxydans*) was synthesized by GenScript (Piscataway, NJ) and codon optimized for *E. coli*. The sequence was then incorporated into the pET-28a plasmid using the *Nde*I and *Xho*I restriction sites, following standard molecular cloning procedures. The gene and protein sequences are shown in Supplementary Data 1.

The MR gene (from *Pseudomonas putida*) was synthesized by GenScript (Piscataway, NJ) and codon optimized for *E. coli*. The sequence was then incorporated into the pET-28a plasmid using the *Nde*I and *Xho*I restriction sites, following standard molecular cloning procedures. The gene and protein sequences are shown in Supplementary Data 1.

The BH32.14 gene (from *Pyrococcus horikoshii*) was synthesized by GenScript (Piscataway, NJ) and codon optimized for *E. coli*. The sequence was then incorporated into the pET-28a plasmid using the *Nde*I and *Xho*I restriction sites,

following standard molecular cloning procedures.The gene and protein sequences are shown in Supplementary Data 1.

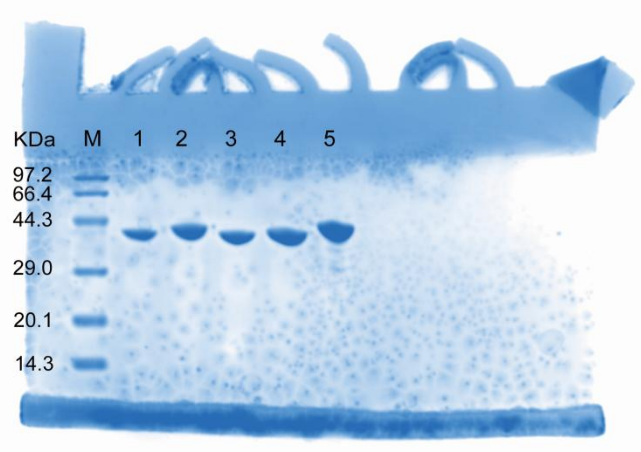

Supplementary Figure 2. SDS-PAGE analysis of different old yellow enzymes.

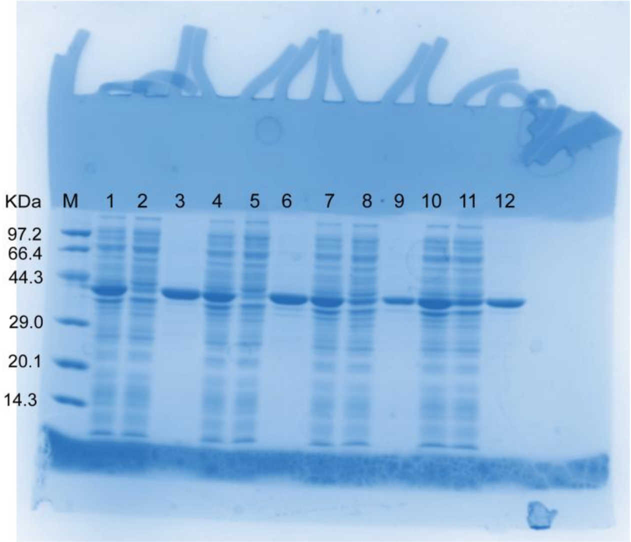

Supplementary Figure 9. SDS-PAGE analysis of the expression of representative mutants of E59 residues.
