# Supplementary material for: Unlocking the function promiscuity of old yellow enzyme to catalyze asymmetric Morita-Baylis-Hillman reaction
Source: Nat Commun. 2024 Jul 9;15:5737. doi: 10.1038/s41467-024-50141-2 (PMC11233575; doi:10.1038/s41467-024-50141-2)
Supplement: Supplementary file 3 — Description of Additional Supplementary Files [file 41467_2024_50141_MOESM3_ESM.pdf]

Description of Additional Supplementary Files

File Name: Supplementary Data 1

Description: Primers, amino acid of key enzymes and the DNA sequences of their encoding genes used.

File Name: Supplementary Data 2

Description: Atomic coordinates in the DFT calculations.
