# Supplementary material for: Unlocking the function promiscuity of old yellow enzyme to catalyze asymmetric Morita-Baylis-Hillman reaction
Source: Nat Commun. 2024 Jul 9;15:5737. doi: 10.1038/s41467-024-50141-2 (PMC11233575; doi:10.1038/s41467-024-50141-2)
Supplement: Supplementary file 5 — Supplementary Data 2 [file 41467_2024_50141_MOESM5_ESM.docx]

**Supplementary Data 2. Atomic coordinates in the DFT calculations.**

Cartesian coordinates

**RC**

*G*(Water) = -1462.95667 Hartree

...........................

O -0.518028 2.301969 2.981756

O 4.091132 -3.049440 -0.604630

O 4.376547 -2.287754 1.413183

N 3.999377 -2.160150 0.245473

C -1.052222 1.926975 0.652906

C 1.656905 2.826166 -1.285449

C -0.275779 0.064136 1.993850

C -0.505329 -0.390469 0.538294

C -0.609702 1.556523 2.006417

C -0.977246 0.853331 -0.162393

C 2.277206 1.531152 -0.905760

C 2.395629 0.493281 -1.844363

C 2.732567 1.352158 0.408497

C 2.962699 -0.720507 -1.474351

C 3.302452 0.141630 0.795505

C 3.405335 -0.873859 -0.156161

H 0.752182 -0.084790 2.340543

H -0.933161 -0.451094 2.704327

H -1.274585 -1.170366 0.447988

H 2.033412 0.645549 -2.855664

H 2.633924 2.155182 1.133757

H 3.058410 -1.535389 -2.180985

H 3.653727 -0.016691 1.807401

H -1.368863 2.929797 0.386294

H 0.406888 -0.775372 0.064677

H -1.255086 0.844381 -1.211292

P -4.236014 -1.026942 -0.470566

O -4.267145 0.277468 0.647521

O -5.731992 -1.395997 -0.572141

O -3.616682 -0.423522 -1.754560

O -3.352105 -2.115050 0.186708

H -3.401735 0.719603 0.628164

H 1.588736 3.574856 -0.474109

O 1.240225 3.083128 -2.404446

**[TS0]**

*G*(Water) = -1462.936952 Hartree

...........................

O -1.051377 1.608533 2.583222

O 4.790003 -2.265497 -1.064666

O 4.691474 -2.161473 1.106269

N 4.404502 -1.736569 -0.017277

C -1.369951 1.278553 0.214106

C 1.092649 2.910285 -0.377232

C -0.619090 -0.574059 1.536027

C -0.459946 -0.919116 0.044272

C -1.040805 0.899627 1.547007

C -1.158877 0.215076 -0.687205

C 1.953569 1.701854 -0.295896

C 2.426339 1.083178 -1.464776

C 2.298786 1.188821 0.962997

C 3.231086 -0.046539 -1.381358

C 3.108127 0.060952 1.064564

C 3.559198 -0.539341 -0.113232

H 0.295426 -0.717604 2.122030

H -1.420201 -1.167164 1.991289

H -0.879429 -1.889349 -0.226174

H 2.148504 1.490010 -2.431468

H 1.917609 1.661463 1.863616

H 3.597688 -0.542711 -2.271355

H 3.377797 -0.352323 2.028334

H -1.698224 2.275183 -0.064957

H 0.597977 -0.915499 -0.249129

H -0.916685 0.398172 -1.728137

P -3.981573 -0.966196 -0.398205

O -4.371480 0.500171 0.346233

O -5.237323 -1.332928 -1.194533

O -2.781361 -0.575627 -1.363806

O -3.556539 -1.938751 0.706292

H -3.531677 0.934374 0.597029

H 0.712928 3.285190 0.589775

O 0.837085 3.494752 -1.421821

**Int1**

*G*(Water) = -1462.942494 Hartree

...........................

O -1.081875 1.853435 2.457507

O 4.766490 -2.180151 -0.989198

O 4.861731 -1.807207 1.153511

N 4.434514 -1.546284 0.021203

C -1.400990 1.172419 0.165062

C 0.746083 2.780971 -0.557411

C -0.566510 -0.435309 1.718902

C -0.438212 -1.025923 0.302688

C -1.049704 1.005586 1.509834

C -1.205528 -0.062287 -0.642447

C 1.689736 1.644222 -0.411873

C 2.109179 0.918508 -1.540985

C 2.197472 1.319381 0.857169

C 3.007355 -0.132336 -1.406058

C 3.101523 0.275417 1.009092

C 3.492567 -0.440511 -0.128835

H 0.380957 -0.439481 2.272083

H -1.309571 -0.972973 2.319581

H -0.838453 -2.039023 0.223823

H 1.717180 1.184104 -2.517199

H 1.862190 1.875001 1.727989

H 3.330866 -0.707981 -2.264529

H 3.493846 0.009834 1.982796

H -1.799430 2.094377 -0.248427

H 0.612130 -1.053069 -0.005532

H -0.646716 0.112286 -1.570445

P -3.780391 -1.036995 -0.316830

O -4.309859 0.459243 0.183593

O -4.822167 -1.553709 -1.289310

O -2.448887 -0.663392 -1.208257

O -3.406001 -1.864253 0.898752

H -3.536211 0.935123 0.557109

H 0.416078 3.243418 0.388068

O 0.426016 3.258092 -1.645613

**[TS1]**

*G*(Water) = -1462.933584 Hartree

...........................

O -1.649715 -1.862012 1.862685

O -3.603271 -2.367118 -1.381475

O 4.918471 0.179194 0.706387

O 4.904322 -0.883835 -1.193648

N 4.341979 -0.498759 -0.157329

C -2.049737 -0.643017 -0.718410

C -1.159580 -1.938416 0.691594

C -4.322304 -0.566977 0.115824

C -3.496196 0.549297 0.779845

C -3.320124 -1.314596 -0.768408

C -2.296380 0.746096 -0.164447

C 0.270988 -1.527553 0.450304

C 0.966493 -0.772151 1.408417

C 0.954791 -1.958694 -0.702383

C 2.298676 -0.428026 1.218487

C 2.286994 -1.628196 -0.914400

C 2.949353 -0.856062 0.052210

H -5.114316 -0.166342 -0.533129

H -4.802288 -1.250188 0.827536

H -3.102270 0.199183 1.738288

H 0.436699 -0.445714 2.295412

H 0.429791 -2.554677 -1.444730

H 2.831900 0.168739 1.948263

H 2.812503 -1.955095 -1.803375

H -1.446936 -2.789140 0.043995

H -4.053191 1.477831 0.943590

H -2.554877 1.446654 -0.971414

P -0.224365 2.451664 -0.146883

O 0.571610 1.563145 -1.306625

O -1.042516 3.459938 -0.926061

O -1.191882 1.331807 0.567685

O 0.751879 2.890553 0.923174

H 1.136901 0.890700 -0.890141

H -1.371322 -0.769218 -1.558515

**Int2**

*G*(Water) = -1462.936439 Hartree

...........................

O -1.387371 -1.692026 1.953596

O -3.836367 -2.690265 0.072523

O 5.087448 0.385878 0.562788

O 5.031167 -0.579115 -1.387328

N 4.498032 -0.267083 -0.311680

C -1.884743 -1.317532 -0.456842

C -0.962068 -1.918922 0.695276

C -4.149649 -0.311978 -0.382209

C -3.240711 0.531007 -1.290572

C -3.358039 -1.595360 -0.189489

C -1.812551 0.183104 -0.816866

C 0.482630 -1.468422 0.399147

C 1.191581 -0.727913 1.358651

C 1.139784 -1.816724 -0.795389

C 2.507221 -0.328795 1.139344

C 2.451347 -1.428263 -1.042477

C 3.127362 -0.681495 -0.067546

H -5.154464 -0.508193 -0.767142

H -4.249018 0.161754 0.605747

H -3.419744 1.606297 -1.230850

H 0.676075 -0.470707 2.277076

H 0.617940 -2.404485 -1.546747

H 3.049365 0.248251 1.879563

H 2.950660 -1.693200 -1.966839

H -0.951088 -3.011643 0.435599

H -3.361325 0.219847 -2.336462

H -1.063970 0.364390 -1.594198

P -0.724866 2.424043 0.175012

O 0.694081 2.006274 -0.580914

O -1.454284 3.304238 -0.816194

O -1.469347 0.964904 0.344030

O -0.443232 2.890983 1.587099

H 1.221822 1.423564 -0.005872

H -1.659248 -1.866153 -1.384258

**Int3**

*G*(Water) = -1463.426674 Hartree

...........................

O -1.262856 -1.816363 1.836055

O -3.779428 -2.374696 0.544353

O 5.074867 0.468584 0.627986

O 5.062462 -0.357492 -1.384680

N 4.519753 -0.137543 -0.295680

C -1.844050 -1.349833 -0.535617

C -0.872786 -2.011002 0.480186

C -4.091300 -0.326721 -0.760036

C -3.051476 0.462301 -1.576240

C -3.317157 -1.486613 -0.158605

C -1.700901 0.137553 -0.903508

C 0.548279 -1.517915 0.298513

C 1.218423 -0.827729 1.319099

C 1.211258 -1.756596 -0.915681

C 2.523831 -0.375988 1.134313

C 2.511383 -1.307304 -1.122243

C 3.153245 -0.617287 -0.090251

H -4.948761 -0.690443 -1.335727

H -4.489701 0.263497 0.075521

H -3.225629 1.539218 -1.586878

H 0.711952 -0.638909 2.257227

H 0.709852 -2.298224 -1.712770

H 3.044259 0.157874 1.920356

H 3.021453 -1.486721 -2.060602

H -0.879593 -3.089457 0.263437

H -3.025826 0.106974 -2.613440

H -0.848305 0.313944 -1.565429

P -0.839605 2.388725 0.281865

O 0.640378 2.074285 -0.398646

O -1.552309 3.278701 -0.709224

O -1.526171 0.880533 0.313631

O -0.676434 2.771471 1.734675

H 1.193517 1.554379 0.210256

H -2.133370 -2.236908 1.942621

H -1.750617 -1.900685 -1.485568

**Int4**

*G*(Water) = -1462.953546 Hartree

...........................

O -1.001789 3.033517 1.443154

O -2.469219 3.132916 -0.799956

O 4.672382 0.499698 -1.728110

O 5.103482 -0.452935 0.180414

N 4.348714 0.172611 -0.577645

C -1.991483 1.086787 0.373935

C -0.919604 1.601514 1.297011

C -3.604684 1.004726 -1.376040

C -3.658878 -0.322075 -0.590471

C -2.628821 1.864729 -0.572249

C -2.364956 -0.344539 0.251214

C 0.481880 1.230927 0.814386

C 0.892953 1.577599 -0.484438

C 1.377811 0.546047 1.648507

C 2.157553 1.244119 -0.947580

C 2.650373 0.189107 1.202336

C 3.024156 0.540546 -0.097018

H -4.583612 1.492477 -1.468408

H -3.218327 0.868825 -2.397021

H -3.732148 -1.212310 -1.222563

H 0.197112 2.099295 -1.134870

H 1.080314 0.292480 2.663450

H 2.475819 1.506708 -1.949302

H 3.340832 -0.344547 1.844493

H -1.041061 1.171212 2.302795

H -4.521541 -0.321985 0.087651

H -2.488552 -0.867515 1.206705

P -0.869835 -2.597981 -0.056119

O -0.222450 -2.360097 1.463085

O -2.073377 -3.489950 0.172874

O -1.301738 -1.083261 -0.492795

O 0.234270 -3.008229 -1.012903

H 0.543715 -1.762575 1.403920

H -1.476311 3.349194 0.624786

**[TS2]**

*G*(Water) = -1462.944276 Hartree

...........................

O 0.898221 -3.070511 1.514554

O 2.162660 -3.178026 -0.923630

O -4.694054 -0.421487 -1.723142

O -5.110580 0.528308 0.189240

N -4.368205 -0.105764 -0.571610

C 1.971322 -1.185547 0.406718

C 0.862874 -1.641387 1.323051

C 3.556694 -1.198320 -1.395555

C 3.761013 0.067634 -0.538335

C 2.493884 -1.989355 -0.638961

C 2.555631 0.085691 0.378551

C -0.520103 -1.235397 0.820583

C -0.936600 -1.575779 -0.477983

C -1.402804 -0.534616 1.655142

C -2.195540 -1.216324 -0.940743

C -2.668857 -0.155383 1.209281

C -3.047691 -0.499637 -0.089765

H 4.465927 -1.794088 -1.534466

H 3.170660 -0.954614 -2.394262

H 3.843823 0.988777 -1.118404

H -0.259311 -2.118723 -1.130066

H -1.099927 -0.281080 2.667959

H -2.517589 -1.471729 -1.943028

H -3.347913 0.392634 1.851196

H 0.998803 -1.197498 2.316524

H 4.667502 -0.021885 0.076630

H 2.527611 0.756063 1.230391

P 0.989199 2.677617 -0.086618

O 0.226871 2.320914 1.385051

O 2.199150 3.510800 0.347667

O 1.357137 1.257195 -0.668197

O -0.047338 3.371727 -0.982597

H -0.450671 1.640647 1.232723

H 1.225048 -3.443246 0.661857

**PC**

*G*(Water) = -1462.960509 Hartree

...........................

O 1.716285 -3.459951 -0.512006

O 2.624134 -1.189595 -1.924861

O -4.382615 -0.087946 -1.248307

O -4.731131 -0.384094 0.878328

N -4.013947 -0.453557 -0.126477

C 2.291581 -1.301535 0.459437

C 1.352491 -2.488350 0.474201

C 3.517005 0.574662 -0.463394

C 3.619694 0.577129 1.075738

C 2.793727 -0.709363 -0.797232

C 2.767477 -0.587485 1.499255

C -0.082969 -1.995013 0.298165

C -0.551668 -1.581588 -0.960335

C -0.932713 -1.902441 1.410163

C -1.840496 -1.079663 -1.108646

C -2.225734 -1.400537 1.281901

C -2.661407 -0.992876 0.019476

H 4.475072 0.642919 -0.987910

H 2.859202 1.388292 -0.811624

H 3.250633 1.506147 1.525326

H 0.097613 -1.640399 -1.827259

H -0.580817 -2.218564 2.388139

H -2.203056 -0.750436 -2.074571

H -2.883753 -1.325423 2.138849

H 1.425068 -2.995804 1.440599

H 4.651391 0.441745 1.427484

H 2.559656 -0.805623 2.543823

P 0.033552 2.826704 -0.086323

O 0.216159 1.560615 1.054946

O 0.475035 4.060197 0.731059

O 0.977850 2.438827 -1.250890

O -1.464558 2.826153 -0.476600

H 0.221980 0.711130 0.585979

H 1.990158 -2.970942 -1.314388

**RC^a^**

*G*(Water) = -1463.431056 Hartree

...........................

O -4.023018 -0.180690 -2.126348

O -3.920230 -1.555451 1.156053

O 2.120192 2.671130 -0.400883

O 1.409216 2.835862 1.651181

N 1.252644 2.539633 0.462909

C -1.667547 -1.097243 1.904921

C -3.845705 0.578573 -1.184178

C -1.997034 -2.503731 -0.040681

C -0.490511 -2.333865 0.243307

C -2.700673 -1.692422 1.047852

C -0.444808 -1.442451 1.456805

C -2.516452 1.082650 -0.764070

C -1.355337 0.687381 -1.448261

C -2.427699 1.944136 0.340182

C -0.109589 1.157483 -1.049872

C -1.186384 2.413129 0.763851

C -0.055911 2.017591 0.048974

H -2.331237 -3.546007 0.009764

H -2.296646 -2.118597 -1.021854

H 0.058991 -1.871462 -0.585985

H -1.438840 0.002838 -2.286180

H -3.327184 2.240987 0.872527

H 0.801965 0.820535 -1.530571

H -1.092823 3.075436 1.616277

H -4.701519 0.933324 -0.580235

H 0.003522 -3.292549 0.452825

H 0.499052 -1.111172 1.876095

P 3.217479 -0.906928 -0.443657

O 3.333618 -2.466215 0.119725

O 4.635528 -0.390268 -0.513226

O 2.273682 -0.882949 -1.623996

O 2.415805 -0.141430 0.789995

H 3.032499 0.092464 1.503581

H 2.486884 -2.930072 0.006315

H -1.897780 -0.456524 2.748955

**[TS0^a^]**

*G*(Water) = -1463.386898 Hartree

...........................

O -0.951334 1.559840 2.628894

O 4.780122 -2.206789 -1.094590

O 4.748883 -2.061981 1.075995

N 4.416416 -1.666203 -0.045513

C -1.415204 1.207228 0.288187

C 0.987218 2.896071 -0.385204

C -0.572524 -0.628311 1.575131

C -0.446603 -0.978569 0.081646

C -1.000571 0.847371 1.590489

C -1.204153 0.130411 -0.661880

C 1.878868 1.710102 -0.309042

C 2.330729 1.081674 -1.481128

C 2.274144 1.228349 0.947727

C 3.163940 -0.027809 -1.402641

C 3.111783 0.121023 1.043853

C 3.540648 -0.490218 -0.136710

H 0.365057 -0.757586 2.126291

H -1.339780 -1.229643 2.077304

H -0.828479 -1.969136 -0.175387

H 2.015734 1.465504 -2.445872

H 1.907020 1.707279 1.850676

H 3.515777 -0.531107 -2.294592

H 3.420007 -0.268672 2.005891

H -1.698616 2.215642 0.001157

H 0.601662 -0.938540 -0.234098

H -0.804198 0.391258 -1.640899

P -3.900242 -0.831348 -0.477417

O -4.208589 0.458354 0.450774

O -5.104699 -1.211288 -1.276310

O -2.592695 -0.529557 -1.291475

O -3.425264 -2.003251 0.557536

H -4.163151 -2.341047 1.094145

H -3.337253 0.884678 0.684699

H 0.633490 3.283598 0.586478

O 0.680052 3.449359 -1.433137

**Int1^a^**

*G*(Water) = -1463.394278 Hartree

...........................

O -0.973975 1.828032 2.458730

O 4.716911 -2.274350 -1.040972

O 4.734243 -2.045871 1.122610

N 4.380255 -1.690291 -0.005983

C -1.422896 1.213431 0.170125

C 0.979158 2.884633 -0.449169

C -0.598276 -0.467314 1.661294

C -0.446295 -0.982536 0.218794

C -1.019190 1.002717 1.504492

C -1.195561 0.032008 -0.664964

C 1.861279 1.693115 -0.345927

C 2.290811 1.021467 -1.502437

C 2.273480 1.251730 0.920403

C 3.116423 -0.091472 -1.398646

C 3.107179 0.143962 1.041532

C 3.511930 -0.511935 -0.123656

H 0.328779 -0.536628 2.240527

H -1.376469 -1.007179 2.214531

H -0.821798 -1.997443 0.069212

H 1.963753 1.374415 -2.474949

H 1.923354 1.764556 1.811388

H 3.449136 -0.628750 -2.278036

H 3.429248 -0.213645 2.011492

H -1.690845 2.185539 -0.233676

H 0.607324 -0.974584 -0.079652

H -0.730189 0.205856 -1.636220

P -3.859696 -0.883400 -0.445676

O -4.189764 0.481120 0.351683

O -5.026641 -1.335357 -1.259506

O -2.521918 -0.652077 -1.247715

O -3.410362 -1.960758 0.694474

H -4.157227 -2.251987 1.245965

H -3.318339 0.929448 0.561955

H 0.603339 3.277804 0.511842

O 0.705923 3.436362 -1.507855

**[TS1^a^]**

*G*(Water) = -1463.370124 Hartree

...........................

O -1.629026 -1.574901 1.932116

O -3.728501 -2.686370 -0.872286

O 4.953498 0.246781 0.566846

O 4.955301 -1.056518 -1.176455

N 4.386372 -0.536564 -0.206973

C -2.067083 -0.939703 -0.762058

C -1.137596 -1.847376 0.784626

C -4.199707 -0.581256 0.293606

C -3.642908 0.807408 -0.061191

C -3.369631 -1.541668 -0.546352

C -2.223206 0.555339 -0.634327

C 0.301713 -1.467404 0.491031

C 0.994938 -0.604888 1.355714

C 0.991317 -2.038519 -0.592779

C 2.331013 -0.289419 1.134640

C 2.327686 -1.741004 -0.834584

C 2.986327 -0.860276 0.033655

H -5.272935 -0.700672 0.119713

H -3.979936 -0.835620 1.341862

H -3.591410 1.485514 0.795195

H 0.459855 -0.173315 2.193107

H 0.472809 -2.723954 -1.258410

H 2.862947 0.387044 1.792759

H 2.857216 -2.176732 -1.673062

H -1.368837 -2.835775 0.336141

H -4.260025 1.289017 -0.827757

H -2.091044 1.058977 -1.596431

P -0.343568 2.389789 -0.176729

O 0.851348 1.822157 -1.112687

O -1.024369 3.507579 -0.886228

O -1.219056 1.151722 0.289190

O 0.322624 2.728838 1.254535

H 0.879582 3.526872 1.229329

H 1.348986 1.098665 -0.686626

H -1.480026 -1.312614 -1.598211

**Int2^a^**

*G*(Water) = -1463.375694 Hartree

...........................

O 1.345457 -1.461046 -2.031172

O 3.725253 -2.807276 -0.208385

O -5.097852 0.557245 -0.437489

O -5.114510 -0.721547 1.323563

N -4.548470 -0.246813 0.328789

C 1.820395 -1.407983 0.406340

C 0.902587 -1.847734 -0.822365

C 4.130370 -0.500998 0.485370

C 3.227206 0.306232 1.431331

C 3.290125 -1.724111 0.151110

C 1.806449 0.048738 0.897740

C -0.540172 -1.415470 -0.483056

C -1.210340 -0.513285 -1.326299

C -1.231725 -1.933563 0.626420

C -2.523710 -0.121869 -1.071455

C -2.541747 -1.558411 0.904758

C -3.178046 -0.649502 0.049574

H 5.106142 -0.777832 0.893216

H 4.295991 0.049203 -0.452502

H 3.457832 1.373656 1.465478

H -0.671401 -0.133125 -2.187491

H -0.740708 -2.647637 1.283225

H -3.037023 0.576873 -1.721959

H -3.068777 -1.957139 1.763299

H 0.879361 -2.962931 -0.700047

H 3.295479 -0.089176 2.452024

H 1.029403 0.217917 1.647287

P 0.851376 2.361389 -0.009908

O -0.609589 2.054561 0.606793

O 1.543005 3.381586 0.823709

O 1.552033 0.948614 -0.232629

O 0.642360 2.737103 -1.564697

H 0.489572 3.689696 -1.695432

H -1.103376 1.383744 0.091513

H 1.538544 -2.022584 1.274132

**Int3^a^**

*G*(Water) = -1463.860459 Hartree

...........................

O 1.250240 -1.589538 -1.950631

O 3.723197 -2.428759 -0.611142

O -5.015638 0.740287 -0.452225

O -5.153612 -0.570886 1.278719

N -4.541165 -0.108134 0.310566

C 1.765625 -1.457922 0.472949

C 0.824503 -1.975580 -0.648696

C 4.022735 -0.500212 0.868386

C 2.967581 0.231942 1.717798

C 3.253841 -1.592366 0.143885

C 1.638205 -0.018232 0.984419

C -0.595382 -1.494252 -0.429130

C -1.183504 -0.546202 -1.280574

C -1.328968 -1.986042 0.660586

C -2.480010 -0.089277 -1.046895

C -2.622374 -1.537698 0.913318

C -3.181489 -0.589101 0.054374

H 4.853223 -0.924280 1.441199

H 4.457279 0.154902 0.102291

H 3.164184 1.299474 1.835295

H -0.622878 -0.164656 -2.125018

H -0.889681 -2.727477 1.322027

H -2.940333 0.638955 -1.704103

H -3.188922 -1.914626 1.755799

H 0.827531 -3.072279 -0.579720

H 2.896801 -0.211564 2.717266

H 0.758525 0.130786 1.614157

P 0.949826 2.332956 -0.092218

O -0.510980 2.203177 0.581950

O 1.753094 3.353764 0.628671

O 1.530230 0.844345 -0.188581

O 0.731155 2.561844 -1.670592

H 0.674962 3.504725 -1.907642

H -1.106198 1.589357 0.108441

H 2.101114 -2.028323 -2.119000

H 1.609096 -2.096598 1.356449

**Int4^a^**

*G*(Water) = -1463.398675 Hartree

...........................

O 1.188519 -3.117565 1.310031

O 2.645735 -2.949719 -0.955937

O -4.567512 -0.590139 -1.736215

O -5.040579 0.242483 0.217639

N -4.264909 -0.318742 -0.567081

C 2.084100 -1.048075 0.400129

C 1.034437 -1.687070 1.272757

C 3.670608 -0.734433 -1.357557

C 3.653018 0.525807 -0.469048

C 2.749212 -1.711403 -0.626881

C 2.391807 0.373529 0.387446

C -0.378043 -1.342772 0.801412

C -0.768901 -1.629588 -0.517443

C -1.299470 -0.730374 1.664724

C -2.039812 -1.306979 -0.972831

C -2.580521 -0.388084 1.226676

C -2.931931 -0.676930 -0.093600

H 4.673746 -1.156806 -1.490026

H 3.267396 -0.538016 -2.360795

H 3.651946 1.467059 -1.026042

H -0.055534 -2.095400 -1.190813

H -1.018027 -0.525452 2.695057

H -2.341845 -1.521383 -1.990867

H -3.291713 0.087873 1.891185

H 1.130262 -1.330445 2.308514

H 4.522521 0.535893 0.199396

H 2.417026 0.890508 1.349977

P 0.699381 2.556327 0.063621

O -0.267258 2.347490 1.343818

O 1.717581 3.608147 0.355918

O 1.214587 1.135576 -0.391971

O -0.390491 2.942130 -1.078369

H 0.016184 3.098756 -1.948933

H -0.782065 1.518102 1.295502

H 1.662064 -3.353388 0.469097

**[TS2^a^]**

*G*(Water) = -1463.394881 Hartree

...........................

O 1.172472 -3.125802 1.328956

O 2.586487 -2.962820 -0.976211

O -4.571831 -0.589230 -1.731944

O -5.036149 0.266939 0.213740

N -4.265731 -0.307802 -0.566198

C 2.079173 -1.065962 0.406418

C 1.025265 -1.694786 1.282039

C 3.654314 -0.767965 -1.367479

C 3.665810 0.484754 -0.468437

C 2.720619 -1.734239 -0.639104

C 2.419963 0.335072 0.403372

C -0.384331 -1.344615 0.807694

C -0.778940 -1.642970 -0.507519

C -1.300003 -0.716852 1.665843

C -2.048149 -1.315599 -0.964582

C -2.579088 -0.370197 1.225971

C -2.934321 -0.670240 -0.090797

H 4.647563 -1.208764 -1.512222

H 3.245591 -0.555031 -2.364823

H 3.666770 1.430436 -1.017142

H -0.071006 -2.122721 -1.176844

H -1.015538 -0.502514 2.693360

H -2.353119 -1.538597 -1.979885

H -3.286019 0.117547 1.886456

H 1.124311 -1.333580 2.315216

H 4.545155 0.480334 0.187574

H 2.427931 0.870215 1.354133

P 0.721507 2.567997 0.057360

O -0.257809 2.366782 1.333138

O 1.749228 3.607735 0.368278

O 1.216269 1.150835 -0.407300

O -0.362561 2.998044 -1.080249

H 0.049655 3.152180 -1.948466

H -0.774789 1.539694 1.278709

H 1.620281 -3.372818 0.479147

**PC^a^**

*G*(Water) = -1463.43474 Hartree

...........................

O 1.538749 -2.459495 -1.724490

O 4.164901 -2.049782 -0.742066

O -4.790399 -0.001116 -0.664194

O -4.865744 -0.964591 1.284849

N -4.271137 -0.655641 0.246401

C 2.139684 -1.251979 0.292716

C 1.220537 -2.281448 -0.338259

C 4.240056 -0.140079 0.807656

C 3.075950 0.542856 1.553862

C 3.588030 -1.265387 0.018329

C 1.863074 -0.246179 1.147801

C -0.237424 -1.891625 -0.214321

C -0.872293 -1.160654 -1.229322

C -0.949213 -2.216709 0.949027

C -2.197991 -0.756678 -1.089065

C -2.272367 -1.813864 1.112537

C -2.879696 -1.085347 0.087216

H 4.996711 -0.559850 1.480311

H 4.761404 0.531603 0.116307

H 2.935649 1.594226 1.273138

H -0.322850 -0.903739 -2.126859

H -0.466176 -2.785976 1.738372

H -2.694761 -0.192627 -1.869248

H -2.824637 -2.059234 2.011217

H 1.370524 -3.237258 0.188430

H 3.201388 0.523175 2.644184

H 0.872260 0.013875 1.504629

P 0.296979 2.835415 -0.254517

O -0.641277 1.811411 0.656023

O 1.297856 3.434912 0.706205

O 0.702248 2.139618 -1.532037

O -0.793221 4.004797 -0.709315

H -0.982742 4.601083 0.034629

H -1.125582 1.197002 0.077475

H 2.507110 -2.587498 -1.768517

**RC’**

*G*(Water) = -1602.715784 Hartree

...........................

O -0.934470 -3.969033 1.648521

O 2.164371 -3.653212 -0.492083

O -4.336079 1.978516 -0.861566

O -4.588949 0.437364 -2.376215

N -4.163183 0.833831 -1.287746

C 1.756892 -1.611583 0.737853

C -1.149286 -2.800923 1.935043

C 0.973372 -1.783721 -1.549193

C 0.595581 -0.410849 -0.958358

C 1.705372 -2.514353 -0.421931

C 1.148664 -0.446418 0.443004

C -1.950591 -1.873349 1.097423

C -2.144344 -0.554567 1.531937

C -2.492582 -2.302781 -0.125864

C -2.878880 0.340884 0.757820

C -3.223148 -1.420833 -0.913249

C -3.402968 -0.111420 -0.452971

H 1.636122 -1.706832 -2.418348

H 0.102434 -2.371614 -1.858559

H -0.487991 -0.243421 -0.939976

H -1.709098 -0.214658 2.467289

H -2.328375 -3.324546 -0.451969

H -3.010815 1.365788 1.079367

H -3.641421 -1.729180 -1.863290

H -0.740645 -2.365440 2.866026

H 2.231925 -1.880003 1.675094

C 5.095318 -1.100171 -0.749261

H 6.174834 -0.970808 -0.604679

C 4.430034 0.217187 -1.148055

H 4.837384 0.566579 -2.103845

H 3.357288 0.058328 -1.292292

S 4.658437 1.541816 0.130260

H 4.944655 -1.859215 -1.528970

H 4.670918 -1.484238 0.185442

C -0.553784 2.927982 1.198817

O -1.374568 2.830625 2.124309

O 0.725861 2.749264 1.375670

C -1.012259 3.240569 -0.220576

H -0.461596 4.098680 -0.621624

H -0.805061 2.384115 -0.873176

H -2.083589 3.446132 -0.244532

O 2.306716 3.115657 -0.422599

H 3.321155 2.424275 -0.197857

H 1.469912 2.922142 0.419779

H 1.967858 2.860275 -1.296595

H 1.022438 0.427587 -1.522664

H 1.051550 0.402821 1.112505

**[TS0’]**

*G*(Water) = -1602.700811 Hartree

...........................

O -0.336961 -1.659145 3.835421

O 1.277603 -3.692076 -1.004890

O -4.115521 0.173486 -2.132690

O -3.545463 -1.911088 -2.380510

N -3.569437 -0.859391 -1.734823

C 1.677267 -1.914964 0.583017

C -0.924891 -0.715266 3.326272

C 0.871781 -1.339432 -1.594259

C 0.803009 -0.097191 -0.686873

C 1.306963 -2.476813 -0.658767

C 1.546609 -0.506413 0.588666

C -1.627106 -0.775265 2.023678

C -2.283560 0.370298 1.550003

C -1.612489 -1.952786 1.256157

C -2.933574 0.349497 0.318396

C -2.257190 -1.989638 0.025478

C -2.906451 -0.834016 -0.421225

H 1.608258 -1.226549 -2.400536

H -0.088233 -1.580180 -2.066075

H -0.235577 0.125969 -0.417320

H -2.273417 1.290756 2.125851

H -1.084964 -2.824146 1.625589

H -3.427929 1.233176 -0.063189

H -2.247750 -2.882873 -0.586190

H -0.955580 0.266797 3.836069

H 2.031737 -2.504061 1.423962

C 5.735758 -0.376797 -1.055561

H 6.191942 0.620101 -1.062074

C 4.208663 -0.293245 -0.995403

H 3.823906 0.226670 -1.880865

H 3.778284 -1.303813 -0.993761

S 3.583413 0.540595 0.517540

H 6.051555 -0.899482 -1.968901

H 6.133200 -0.925477 -0.193512

C -1.157258 3.386225 0.134815

O -2.217143 3.496621 0.734840

O -0.012361 3.298469 0.821912

C -1.053327 3.322563 -1.373256

H -0.392565 4.112888 -1.746106

H -0.626926 2.361005 -1.678904

H -2.043760 3.434863 -1.815803

O 2.285951 3.203852 -0.530765

H 2.762714 2.371133 -0.235640

H 0.801023 3.244525 0.235714

H 2.278601 3.174577 -1.501130

H 1.211579 0.802170 -1.151657

H 1.301272 0.026655 1.504234

**Int 1’**

*G*(Water) = -1602.712603 Hartree

...........................

O -0.083509 -4.043956 1.803936

O 2.026510 -3.647680 -0.613710

O -4.351236 1.289089 -0.745607

O -4.477327 -0.342392 -2.184438

N -4.019825 0.162044 -1.147105

C 1.845117 -1.617205 0.674163

C -0.244341 -2.827402 1.923000

C 1.202120 -1.568570 -1.627380

C 0.868440 -0.200205 -1.003198

C 1.744102 -2.419859 -0.471166

C 1.565080 -0.189681 0.387525

C -1.187220 -2.038794 1.095004

C -1.579049 -0.753170 1.513748

C -1.760868 -2.595726 -0.064769

C -2.514159 -0.024899 0.789015

C -2.681251 -1.875917 -0.810313

C -3.052090 -0.594359 -0.372962

H 1.970350 -1.501874 -2.409638

H 0.324734 -2.049640 -2.077950

H -0.210469 -0.113964 -0.844817

H -1.148961 -0.324033 2.414692

H -1.460899 -3.591232 -0.374473

H -2.814800 0.965947 1.106684

H -3.114621 -2.287053 -1.713813

H 0.210283 -2.276445 2.764469

H 2.337687 -1.942213 1.585399

C 5.538248 0.768826 -0.940019

H 5.522934 1.856753 -1.073054

C 4.124889 0.185537 -0.930004

H 3.615159 0.396800 -1.875530

H 4.150313 -0.900182 -0.792365

S 3.134306 0.892245 0.449691

H 6.110159 0.335924 -1.769625

H 6.068066 0.544675 -0.007299

C -1.148011 3.331633 0.941766

O -2.049550 3.201029 1.757649

O 0.062532 3.735129 1.349265

C -1.311028 3.068051 -0.538718

H -1.102291 3.982105 -1.106413

H -0.600123 2.303592 -0.867880

H -2.327702 2.733404 -0.745293

O 1.997731 3.719449 -0.505694

H 2.406402 2.830185 -0.334910

H 0.728003 3.765928 0.601460

H 1.742100 3.718725 -1.442449

H 1.169647 0.645344 -1.629396

H 0.952846 0.322100 1.141508

**[TS1’]**

*G*(Water) = -1602.700811 Hartree

...........................

O -0.173641 -1.835656 -2.783975

O 2.013529 -4.114834 -0.723679

O -5.399762 -0.179275 2.026916

O -5.049673 1.593651 0.814149

N -4.791213 0.423076 1.130276

C 0.821130 -2.092129 -0.175129

C -0.555531 -2.295731 -1.653423

C 2.880006 -1.941519 -1.444955

C 2.289196 -0.527959 -1.276905

C 1.899271 -2.874749 -0.737268

C 1.228430 -0.642562 -0.146875

C -1.680115 -1.599285 -0.924624

C -2.399912 -2.245622 0.096897

C -2.023506 -0.280958 -1.268729

C -3.423404 -1.595746 0.773576

C -3.037293 0.393589 -0.596919

C -3.728202 -0.272347 0.421934

H 3.865583 -2.052249 -0.974913

H 2.988623 -2.238726 -2.494839

H 1.756465 -0.245356 -2.189646

H -2.148494 -3.269225 0.364500

H -1.482272 0.201035 -2.076252

H -3.980683 -2.092061 1.558833

H -3.296187 1.414261 -0.852291

H -0.589700 -3.394107 -1.506660

H 0.282396 -2.512823 0.670371

C 3.864946 -0.752265 3.231748

H 4.123755 0.305656 3.354575

C 3.314143 -1.033643 1.833306

H 4.054523 -0.774895 1.069999

H 3.060451 -2.092449 1.721188

S 1.789128 -0.045489 1.537883

H 4.773652 -1.343490 3.394718

H 3.139615 -1.021579 4.007651

C 0.704747 4.309908 -0.973028

O 0.020588 5.142226 -1.549707

O 1.813582 4.693682 -0.325056

C 0.373678 2.834167 -0.931601

H 1.211594 2.243717 -1.315621

H 0.193547 2.520383 0.102490

H -0.517157 2.637499 -1.528571

O 3.244426 2.767839 0.875890

H 2.790197 1.909513 1.073875

H 2.301836 3.929817 0.099778

H 4.033041 2.531660 0.360510

H 3.046176 0.235149 -1.074619

H 0.387586 0.034602 -0.327457

**Int2’**

*G*(Water) =-1602.699494 Hartree

...........................

O -0.788405 -2.188758 2.419045

O -3.468963 -3.303407 0.300123

O 5.010134 -1.428014 -1.975515

O 5.336922 -0.214295 -0.199650

N 4.639425 -0.980354 -0.880413

C -1.639338 -1.715701 0.142587

C -0.587994 -2.459665 1.118318

C -3.786929 -1.068183 1.220111

C -2.785500 0.089425 1.370255

C -3.023334 -2.183331 0.522674

C -1.637668 -0.192902 0.360844

C 0.810805 -2.091878 0.583357

C 1.281058 -2.586200 -0.647112

C 1.641708 -1.241203 1.326766

C 2.532621 -2.233664 -1.136919

C 2.897755 -0.867720 0.856782

C 3.330828 -1.367229 -0.376218

H -4.648675 -0.807398 0.591859

H -4.187431 -1.431008 2.173249

H -2.346033 0.066086 2.370753

H 0.655266 -3.258316 -1.230292

H 1.278547 -0.882182 2.284300

H 2.892851 -2.613900 -2.085381

H 3.536196 -0.203028 1.426949

H -0.736714 -3.534604 0.842343

H -1.422748 -2.012526 -0.888261

C -3.609024 1.078735 -3.208879

H -3.505864 2.167181 -3.133143

C -3.403565 0.404895 -1.851808

H -4.140405 0.766638 -1.128573

H -3.508257 -0.681372 -1.940523

S -1.716036 0.775438 -1.215035

H -4.617261 0.859056 -3.578328

H -2.887940 0.714049 -3.948756

C 1.441528 3.962438 0.810619

O 2.566290 4.385661 1.032843

O 0.478511 4.809593 0.420308

C 1.055992 2.504677 0.936708

H 0.183357 2.389387 1.587860

H 0.790798 2.105742 -0.048615

H 1.892645 1.933483 1.338273

O -1.960371 3.760987 0.009857

H -1.947203 2.847524 -0.371862

H -0.405771 4.361589 0.280630

H -2.471625 3.692847 0.832920

H -3.246257 1.070710 1.223607

H -0.683507 0.136410 0.779847

**Int3’**

*G*(Water) =-1603.179935 Hartree

...........................

O -1.499227 3.576673 0.899284

O 0.094153 1.918032 2.592831

O -5.275025 -2.155752 -1.535797

O -5.442815 -2.187101 0.632834

N -4.978822 -1.725968 -0.415644

C 0.283157 2.056935 0.177605

C -1.166537 2.591071 -0.074148

C 0.768516 -0.086832 1.369068

C 0.618760 -0.366063 -0.135097

C 0.365366 1.360272 1.536119

C 0.774375 1.007314 -0.846875

C -2.208479 1.482401 -0.152547

C -2.591703 0.973224 -1.402468

C -2.777586 0.937023 1.009439

C -3.497513 -0.080041 -1.501292

C -3.685539 -0.114663 0.932987

C -4.027914 -0.615104 -0.325930

H 1.807887 -0.194425 1.704922

H 0.159181 -0.729703 2.012129

H -0.387936 -0.746335 -0.340527

H 0.174353 1.029779 -1.760320

H -2.184362 1.404010 -2.312622

H -2.514590 1.333666 1.983962

H -3.791007 -0.476166 -2.465608

H -4.120817 -0.541666 1.828117

H -1.144387 3.110383 -1.037297

H -1.125808 3.303534 1.760010

C 4.936885 1.870965 -0.392893

H 5.396740 1.211721 -1.137828

C 3.535224 1.393111 -0.009899

H 3.576111 0.383254 0.403666

H 3.096600 2.067336 0.732782

S 2.459541 1.380604 -1.509766

H 5.575851 1.867954 0.497578

H 4.918410 2.889178 -0.796815

C 3.097891 -2.893708 0.864525

O 3.704069 -1.859668 1.117395

O 2.848445 -3.305299 -0.384594

C 2.523049 -3.820236 1.903584

H 1.429563 -3.741670 1.883247

H 2.783639 -4.858924 1.678904

H 2.885738 -3.544012 2.894488

O 3.579403 -1.606225 -2.347617

H 4.541557 -1.498634 -2.427768

H 3.195665 -2.644209 -1.054413

H 3.230472 -0.698464 -2.182496

H 1.325828 -1.108041 -0.505879

H 0.910662 2.954253 0.199777

**Int4’**

*G*(Water) =-1602.704612 Hartree

...........................

O -1.178215 0.195673 2.939351

O 0.266088 -1.971754 2.328697

O -6.382375 0.460533 -2.177611

O -6.771971 -1.287969 -0.940675

N -6.072213 -0.314652 -1.260006

C -0.078410 -0.153287 0.793285

C -1.133759 0.599428 1.567727

C 1.602146 -1.752854 0.253419

C 1.342395 -0.877701 -0.988670

C 0.516790 -1.320689 1.237970

C 0.509207 0.327415 -0.478226

C -2.471971 0.387164 0.871274

C -2.872132 1.252527 -0.162589

C -3.291197 -0.709244 1.194391

C -4.047771 1.028853 -0.871455

C -4.472294 -0.947441 0.503875

C -4.838980 -0.074800 -0.531318

H 2.591778 -1.569393 0.695068

H 1.540779 -2.827249 0.039601

H 0.727151 -1.431311 -1.709306

H -0.217444 0.666935 -1.226650

H -2.257597 2.114360 -0.409876

H -2.992801 -1.372519 1.999743

H -4.358236 1.697133 -1.665661

H -5.106550 -1.789414 0.754137

H -0.911127 1.677634 1.548630

H -0.720914 -0.688737 2.973645

C 3.331310 2.785260 1.665731

H 3.983347 3.216173 0.896898

C 2.604396 1.541027 1.154955

H 3.324429 0.777074 0.849677

H 1.958791 1.116397 1.930342

S 1.527487 1.950908 -0.281257

H 3.958740 2.518302 2.525133

H 2.624355 3.558788 1.987446

C 5.141322 -1.283832 -0.809909

O 5.264095 -0.485916 0.111077

O 4.790049 -0.920376 -2.049207

C 5.337556 -2.772402 -0.681062

H 4.366619 -3.270092 -0.787968

H 5.988367 -3.143393 -1.478896

H 5.761541 -3.012868 0.294781

O 4.014530 1.638753 -2.320529

H 4.665804 2.283735 -1.998778

H 4.581221 0.061487 -2.086435

H 3.214356 1.771614 -1.748407

H 2.253843 -0.574712 -1.508195

**[TS2’]**

*G*(Water) =-1602.714227 Hartree

...........................

O 0.634519 1.155076 2.113716

O -0.523494 2.574903 0.109718

O 6.935440 -1.131343 -0.745158

O 7.031084 1.039352 -0.630068

N 6.419200 -0.030669 -0.507335

C 0.057919 0.248787 -0.078594

C 0.904609 0.116842 1.167625

C -1.413138 1.178624 -1.723155

C -0.930914 -0.207705 -2.195626

C -0.588046 1.447431 -0.469732

C -0.247458 -0.798764 -0.968029

C 2.377276 0.082955 0.776408

C 3.006715 -1.144255 0.511643

C 3.108087 1.272425 0.620164

C 4.331053 -1.193488 0.086856

C 4.432735 1.245963 0.200314

C 5.031149 0.007726 -0.066144

H -2.477004 1.169499 -1.453779

H -1.270431 1.971371 -2.466371

H -0.177407 -0.102860 -2.989509

H 0.418447 -1.645783 -1.097052

H 2.456080 -2.072028 0.643261

H 2.630580 2.223101 0.834218

H 4.819445 -2.139268 -0.114403

H 4.999351 2.161248 0.078604

H 0.663028 -0.836681 1.657618

H 0.244240 1.904208 1.601038

C -2.264544 -1.506247 2.758448

H -2.736816 -2.470814 2.978891

C -2.512653 -1.083836 1.308202

H -3.585481 -0.966150 1.125402

H -2.042979 -0.106840 1.122863

S -1.821860 -2.243386 0.063202

H -2.678724 -0.758933 3.449673

H -1.191899 -1.600279 2.963857

C -5.550684 0.851347 -0.061949

O -5.948767 -0.112387 0.581773

O -4.883037 0.736054 -1.214105

C -5.733722 2.289045 0.353637

H -4.754111 2.724472 0.582815

H -6.166912 2.869529 -0.467197

H -6.374234 2.348761 1.234635

O -4.222869 -1.756227 -1.899770

H -4.912159 -2.397634 -1.660822

H -4.708712 -0.232227 -1.435769

H -3.441585 -1.978457 -1.309289

H -1.730647 -0.839893 -2.587828

**PC’**

*G*(Water) =-1602.723727 Hartree

...........................

O -0.147558 -0.876522 1.854324

O 0.062063 -2.841138 -0.160355

O -6.662926 1.456492 -0.327311

O -6.830828 -0.705892 -0.162049

N -6.166981 0.337515 -0.149875

C 0.215610 -0.464017 -0.517308

C -0.480602 -0.023664 0.752316

C 1.094623 -1.963789 -2.212538

C 1.357709 -0.496012 -2.608045

C 0.401780 -1.882391 -0.863636

C 0.756501 0.302828 -1.485127

C -1.988323 0.061681 0.531881

C -2.593254 1.310632 0.330654

C -2.780630 -1.097458 0.505284

C -3.963667 1.413319 0.104177

C -4.151207 -1.016321 0.283052

C -4.726415 0.243141 0.084175

H 2.011740 -2.551543 -2.110487

H 0.442049 -2.486950 -2.921283

H 0.899991 -0.223541 -3.567718

H 0.804449 1.388473 -1.464682

H -1.989697 2.213799 0.352976

H -2.323078 -2.070169 0.653224

H -4.435043 2.376728 -0.047007

H -4.767011 -1.907123 0.260190

H -0.120287 0.970655 1.026631

H -0.149097 -1.795850 1.519688

C 1.799377 2.900224 1.742354

H 2.244309 3.846985 2.073213

C 2.782684 2.101685 0.883282

H 3.680325 1.876380 1.471276

H 2.338492 1.135580 0.617819

S 3.281083 2.977610 -0.677589

H 1.505030 2.330706 2.636461

H 0.890366 3.142529 1.177453

C 3.960319 -1.417966 1.098530

O 4.976095 -0.826366 1.447501

O 3.480898 -1.376864 -0.147172

C 3.097067 -2.239871 2.020060

H 2.190794 -1.665579 2.246948

H 2.781796 -3.166780 1.532097

H 3.631709 -2.457501 2.946104

O 4.635434 0.403372 -1.752224

H 5.584713 0.496276 -1.568461

H 4.005028 -0.725352 -0.713704

H 4.222714 1.282670 -1.451175

H 2.427389 -0.271537 -2.695404

**H_2_PO_4_^-^**

*G*(Water) =-643.8806991 Hartree

...........................

P -0.000250 0.094895 0.127899

O -0.003776 1.602514 0.136706

O -1.296228 -0.362291 -0.805765

O 1.300053 -0.356839 -0.802513

O -0.000476 -0.738370 1.393263

H -1.519693 -1.294918 -0.647777

H 1.526867 -1.288614 -0.644239

**HPO_4_^2-^**

*G*(Water) =-643.407067 Hartree

...........................

P 0.140641 0.027857 -0.000355

O -1.498221 -0.494952 0.006548

O 0.900400 -1.315389 0.005517

O 0.293121 0.853068 -1.301157

O 0.299743 0.868812 1.289646

H -2.069957 0.289821 0.000891

**H_2_O**

*G*(Water) =-76.46445659 Hartree

...........................

O 0.000000 0.118625 0.000000

H 0.768383 -0.474555 0.000000

H -0.768383 -0.474445 0.000000

**-OH**

*G*(Water) =-75.96744184 Hartree

...........................

O 0.000000 0.000000 0.107998

H 0.000000 0.000000 -0.863985
