# Supplementary material for: Unlocking the function promiscuity of old yellow enzyme to catalyze asymmetric Morita-Baylis-Hillman reaction
Source: Nat Commun. 2024 Jul 9;15:5737. doi: 10.1038/s41467-024-50141-2 (PMC11233575; doi:10.1038/s41467-024-50141-2)
Supplement: Supplementary file 6 — Reporting Summary [file 41467_2024_50141_MOESM6_ESM.pdf]

nature portfolio

Corresponding author(s): Wei Song

Last updated by author(s): Jun 15, 2024

Reporting Summary

Nature Portfolio wishes to improve the reproducibility of the work that we publish. This form provides structure for consistency and transparency in reporting. For further information on Nature Portfolio policies, see our Editorial Policies and the Editorial Policy Checklist.

Statistics

For all statistical analyses, confirm that the following items are present in the figure legend, table legend, main text, or Methods section.

n/a	Confirmed	
☐	☒	The exact sample size (*n*) for each experimental group/condition, given as a discrete number and unit of measurement
☐	☒	A statement on whether measurements were taken from distinct samples or whether the same sample was measured repeatedly
☒	☐	The statistical test(s) used AND whether they are one- or two-sided<br>*Only common tests should be described solely by name; describe more complex techniques in the Methods section.*
☐	☒	A description of all covariates tested
☐	☒	A description of any assumptions or corrections, such as tests of normality and adjustment for multiple comparisons
☐	☒	A full description of the statistical parameters including central tendency (e.g. means) or other basic estimates (e.g. regression coefficient) AND variation (e.g. standard deviation) or associated estimates of uncertainty (e.g. confidence intervals)
☒	☐	For null hypothesis testing, the test statistic (e.g. *F*, *t*, *r*) with confidence intervals, effect sizes, degrees of freedom and *P* value noted<br>*Give P values as exact values whenever suitable.*
☒	☐	For Bayesian analysis, information on the choice of priors and Markov chain Monte Carlo settings
☒	☐	For hierarchical and complex designs, identification of the appropriate level for tests and full reporting of outcomes
☒	☐	Estimates of effect sizes (e.g. Cohen's *d*, Pearson's *r*), indicating how they were calculated

Our web collection on statistics for biologists contains articles on many of the points above.

Software and code

Policy information about availability of computer code

Data collection	NanoPhotometer N50 spectrophotometer, HPLC(Waters Alliance e2695, Waters 2545 Binary Gradient Module),GC(GC-2030,SHIMADZU), GC-MS(Trace1310-TSQ8000, Thermo Scientific),MS (LTQ-Orbitrap Velos system), NMR, Bruker 8 QUEST diffractometer(Karlsruhe),JASCO-1700 circular dichroic spectrometer
Data analysis	Microsoft Excel 2016,Snapgene 1.1.3, Microsoft Word 2016, DNAMAN 6.0, Primer 7.0, Adobe Illustrate C56,GrapPad Prims, Unidec, online Consurf Server, DositeScorer,XDS, PyMOL v2.3.3, Dichroweb, H++ server, Gaussian 16, Antechamber module,AutoDock Vina, Amber 18,CYL View, MassLynx,Xcalibur 2.2

For manuscripts utilizing custom algorithms or software that are central to the research but not yet described in published literature, software must be made available to editors and reviewers. We strongly encourage code deposition in a community repository (e.g. GitHub). See the Nature Portfolio guidelines for submitting code & software for further information.

Data

Policy information about availability of data

All manuscripts must include a data availability statement. This statement should provide the following information, where applicable:

- Accession codes, unique identifiers, or web links for publicly available datasets
- A description of any restrictions on data availability
- For clinical datasets or third party data, please ensure that the statement adheres to our policy

Data supporting the findings of this work are available within the manuscript and Supplementary files. The datasets generated and analyzed during the current

study are also available from the corresponding author. Crystal structure of Geobacillus kaustophilus apo-GkOYE.8 was uploaded into the PDB database with PDB number 8X0J (https://www.rcsb.org/structure/unreleased/8X0J). Source data are provided with this paper.

Research involving human participants, their data, or biological material

Policy information about studies with human participants or human data. See also policy information about sex, gender (identity/presentation), and sexual orientation and race, ethnicity and racism.

Reporting on sex and gender	This is not applicable in this study.
Reporting on race, ethnicity, or other socially relevant groupings	This is not applicable in this study.
Population characteristics	This is not applicable in this study.
Recruitment	This is not applicable in this study.
Ethics oversight	This is not applicable in this study.

Note that full information on the approval of the study protocol must also be provided in the manuscript.

Field-specific reporting

Please select the one below that is the best fit for your research. If you are not sure, read the appropriate sections before making your selection.

- [x] Life sciences
- [ ] Behavioural & social sciences
- [ ] Ecological, evolutionary & environmental sciences

For a reference copy of the document with all sections, see nature.com/documents/nr-reporting-summary-flat.pdf

Life sciences study design

All studies must disclose on these points even when the disclosure is negative.

Sample size	Nosample-size calculations were performed. As indicated in the text, all experiments were performed from a single colony, which provides some limited information about the distribution of the measurements and is typical of similar experiments and studies in the field.
Data exclusions	No data were excluded form the analyses.
Replication	All the biochemical and biological experiments were performed in three replicated or more. Data were repeatable on different date.
Randomization	The cells and protein are uniformly and randomly distributed in the medium and buffer. Pipet tips were used to scoop few cells for culturing, the scooping locations are all random.
Blinding	Blinding is not relevant to our study because none of our data is based on qualitative scoring metrics nor does it involve animals or human research participants. As described in the above section for randomization, blinding during group allocation is irrelevant because the samples of bacterial cultures that were split into different conditions were random samplings and there is no control over which cells will be selected and thus, no bias during group allocation.

Reporting for specific materials, systems and methods

We require information from authors about some types of materials, experimental systems and methods used in many studies. Here, indicate whether each material, system or method listed is relevant to your study. If you are not sure if a list item applies to your research, read the appropriate section before selecting a response.

Materials & experimental systems

n/a	Involved in the study	
x		Antibodies
x		Eukaryotic cell lines
x		Palaeontology and archaeology
x		Animals and other organisms
x		Clinical data
x		Dual use research of concern
x		Plants

Methods

n/a	Involved in the study	
x		ChIP-seq
x		Flow cytometry
x		MRI-based neuroimaging

Plants

Seed stocks	This is not applicable in this study.
Novel plant genotypes	This is not applicable in this study.
Authentication	This is not applicable in this study.
